# Safety versus performance: How multi-objective learning reduces barriers to market entry

**DOI:** 10.1073/pnas.2510004122

**Published:** 2025-10-15

**Authors:** Meena Jagadeesan, Michael I. Jordan, Jacob Steinhardt

**Affiliations:** ^a^Department of Electrical Engineering and Computer Science, University of California, Berkeley, CA 94720; ^b^Department of Statistics, University of California, Berkeley, CA 94720; ^c^Inria Paris, Paris 75013, France

**Keywords:** market design, large language models, multi-objective learning, barriers to entry

## Abstract

Emerging marketplaces for large language models and other large-scale machine learning models appear to exhibit market concentration, which has raised concerns about whether there are insurmountable barriers to entry in such markets. In this work, we study this issue from both an economic and an algorithmic point of view, focusing on a phenomenon that reduces barriers to entry. Specifically, an incumbent company risks reputational damage unless its model is sufficiently aligned with safety objectives, whereas a new company can more easily avoid reputational damage. To study this issue formally, we define a multi-objective high-dimensional regression framework that captures reputational damage, and we characterize the number of data points that a new company needs to enter the market. Our results demonstrate how multi-objective considerations can fundamentally reduce barriers to entry—the required number of data points can be significantly smaller than the incumbent company’s dataset size. En route to proving these results, we develop scaling laws for high-dimensional linear regression in multi-objective environments, showing that the scaling rate becomes slower when the dataset size is large, which could be of independent interest.

## Significance

The development of large language models has given rise to emerging markets where companies offer models as a service and compete for user usage. A concern is that the accumulation of data and compute by incumbents creates insurmountable barriers to entry for new companies. We develop a multi-objective high-dimensional regression framework to study market entry, focusing on a phenomenon which challenges this intuition. Our framework captures the reputational damage that companies face due to models’ safety violations. We show how the incumbents face greater threat of reputational damage than new companies, which reduces the amount of data the new company needs to enter the market. We quantify this reduction as a function of the incumbent’s data size.

Large language models and other large-scale machine learning (ML) models have led to an important shift in the information technology landscape, one which has significant economic consequences. Whereas earlier generations of ML models provided the underpinnings for platforms and services, new models—such as language models—are themselves the service. This has led to new markets where companies offer language models as their service and compete for user usage. As in other markets, it is important to reason about market competitiveness: in particular, to what extent there are barriers to entry for new companies.

A widespread concern about these markets is that new companies face insurmountable barriers to entry that drive market concentration (1). The typical argument is that incumbent companies with high market share can purchase or capture significant amounts of data and compute, and then invest these resources into the training of models that achieve even higher performance (2). This suggests that the company’s market share would further increase and that the scale and scope of this phenomenon would place incumbent companies beyond the reach of new companies trying to enter the market. The scale is in fact massive—language assistants such as ChatGPT and Gemini each have hundreds of millions of users (3). In light of the concerns raised by policymakers (1) and regulators (4, 5) regarding market concentration, it is important to investigate the underlying economic and algorithmic mechanisms at play.

While standard arguments assume that market share is determined by model performance, the reality is that the incumbent company risks reputational damage if their model violates safety-oriented objectives. For example, incumbent companies face public and regulatory scrutiny for their model’s safety violations—such as threatening behavior (6), jailbreaks (7), and releasing dangerous information (4)—even when the model performs well in terms of helpfulness and usefulness to users. In contrast, new companies face less regulatory scrutiny since compliance requirements often prioritize models trained with more resources (4, 8), and new companies also may face less public scrutiny given their smaller user bases.

In this work, we use a multi-objective learning framework to show that the threat of reputational damage faced by the incumbent company can reduce barriers to entry. For the incumbent, the possibility of reputational damage creates pressure to align with safety objectives in addition to optimizing for performance. Safety and performance are not fully aligned, so improving safety can reduce performance as a side effect. Meanwhile, the new company faces less of a risk of reputational damage from safety violations. The new company can thus enter the marketplace with significantly less data than the incumbent company, a phenomenon that our model and results formalize.

## Model and Results.

We analyze a stylized marketplace based on multi-objective linear regression. The performance-optimal output and the safety-optimal output are specified by two different linear functions of the input x. The marketplace consists of two companies: an incumbent company and a new company attempting to enter the market. Each company receives their own unlabeled training dataset, decides what fraction of training data points to label according to the performance-optimal versus safety-optimal outputs, and then runs ridge regression. The new company requires a less stringent level of safety to avoid reputational damage than the incumbent company. We characterize the market-entry threshold $N_{E}^{*}$ (*Definition* 1) which captures how much data the new company needs to outperform the incumbent company.

**Fig. 1.**
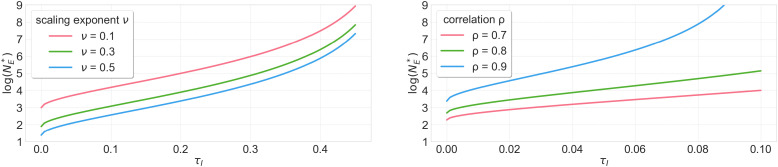
Market-entry threshold $N_{E}^{*}$ as a function of the incumbent’s safety constraint $\tau_{I}$, when the incumbent has infinite data and a safety constraint and the entrant has no safety constraint (*Theorem* 1). The scaling exponent ν captures the data efficiency of learning. The plots show varying values of the scaling exponent ν, where the correlation parameter ρ=0.5 is held fixed (*Left*) and varying values of ρ, where ν=0.34 is held fixed (*Right*). The plots are shown in log space with base e. The market-entry threshold $N_{E}^{*}$ is finite. It is also higher when the constraint $\tau_{I}$ is weaker, when the correlation ρ is stronger, and when the scaling exponent ν is lower.

First, as a warmup, we characterize $N_{E}^{*}$ when the new company faces no safety constraint and the incumbent company has infinitely many data points. Our key finding is that the new company can enter the market with finite data, even when the incumbent company has infinite data (*Theorem* 1; Fig. 1). Specifically, we show that the threshold $N_{E}^{*}$ is finite; moreover, it is increasing in the correlation (i.e., the alignment) between performance and safety, and it is decreasing in a problem-specific scaling law exponent.

**Fig. 2.**
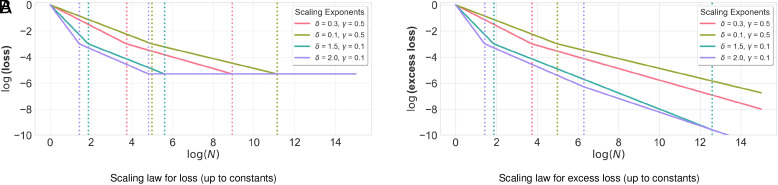
Data scaling laws for multi-objective environments where a fraction α=0.9 of the data is labeled according to the primary objective and a fraction 1−α=0.1 is labeled according to the secondary objective. The plots show, up to constants, the loss and excess loss as a function of the total number of training data points N. The plots are shown in log space with base e. The scaling exponent γ captures how easy it is to represent the data using a small number of directions, and the scaling exponent δ captures how easy it is to capture the ground truth predictors $\beta_{1}$ and $\beta_{2}$ in the eigenbasis of the covariance matrix. (*A*) The loss $\Theta (\underset{\lambda \in (0,1)}{\inf}E\left[L_{1}\left(\hat{\beta }\left(\alpha ,\lambda ,X\right)\right)\right])$ (Theorem 2). (*B*) The excess loss $\Theta (\underset{\lambda \in (0,1)}{\inf}\left(E\left[L_{1}\left(\hat{\beta }\left(\alpha ,\lambda ,X\right)\right)-L_{1}\left(\beta \left(\alpha ,0\right)\right)\right]\right))$ (Theorem 3). The loss and excess loss both take the form $N^{-\nu^{*}}$, but where the scaling exponent $\nu^{*}$ takes on *multiple (two or three) different values* depending on the size of N relative to other parameters. The scaling exponent is smaller when N is larger, thus demonstrating that the scaling rate becomes slower as the dataset size N increases.

**Fig. 3.**
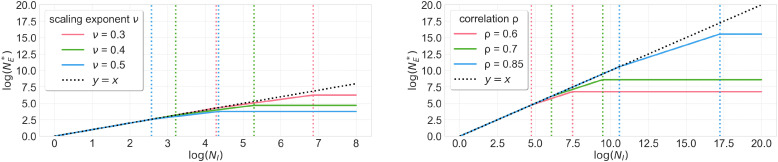
Market-entry threshold $N_{E}^{*}$ as a function of the incumbent dataset size $N_{I}$, when the incumbent has finite data and a safety constraint and the entrant has no safety constraint (*Theorem* 4). The scaling exponent ν captures the data efficiency of learning. The plots show varying values of the scaling exponent ν where the correlation parameter ρ=0.5 is held fixed (*Left*) and varying values of ρ, where ν=0.34 is held fixed (*Right*). The plots are shown in log space with base e. When $N_{I}$ is sufficiently large, the market-entry threshold $N_{E}^{*}$ is asymptotically less than $N_{I}$ (i.e., below the dotted black line). Each curve is the union of three line segments with slope decreasing in $N_{I}$. This demonstrates that the new company can afford to scale up their dataset at a slower rate than the incumbent, when the incumbent’s dataset size is sufficiently large.

Next, we turn to more general environments where the incumbent has finite data $N_{I}<\infty$. We find that the threshold $N_{E}^{*}$ scales sublinearly with the incumbent’s dataset size $N_{I}$, as long as $N_{I}$ is sufficiently large. In fact, the threshold $N_{E}^{*}$ scales at a slower rate as $N_{I}$ increases: that is, $N_{E}^{*}=\Theta \left(N_{I}^{c}\right)$, where the exponent c is decreasing in $N_{I}$ (*Theorem* 4; Figs. 2 and 3). For example, for concrete parameter settings motivated by language models (9), the exponent c decreases from 1 to 0.75 to 0 as $N_{I}$ increases. In general, the exponent c takes on up to three different values depending on $N_{I}$. The exponent c is strictly smaller than 1 as long as $N_{I}$ is sufficiently large.

**Fig. 4.**
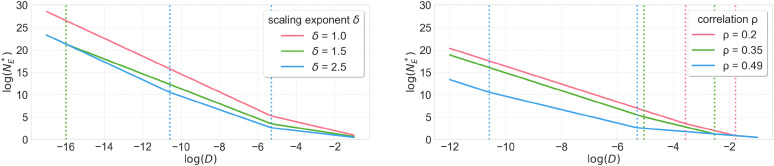
The market-entry threshold $N_{E}^{*}$ when the incumbent has infinite data and companies have a safety constraint, with the incumbent facing a stricter safety constraint than the new company. $N_{E}^{*}$ is shown as a function of the difference D between the infinite-data performance loss of the incumbent and infinite-data performance loss of the new company considering these safety constraints (*Theorem* 5). The scaling exponent δ captures how easy it is to capture the ground truth predictors $\beta_{1}$ and $\beta_{2}$ in the eigenbasis of the covariance matrix. The plots show varying values of the scaling exponent δ where the correlation parameter ρ=0.49 is held fixed (*Left*) and varying values of ρ, where δ=2.5 is held fixed (*Right*). The plots are shown in log space with base e. The market-entry threshold is finite in all cases. Each curve is the union of multiple line segments with slope increasing in magnitude as logD decreases, demonstrating that the new company needs to scale up their dataset at a faster rate as the safety thresholds become closer together.

Finally, we turn to environments where the new company also faces a nontrivial safety constraint, assuming for simplicity that the incumbent company again has infinite data. We find that $N_{E}^{*}$ is finite as long as the new company faces a strictly weaker safety constraint than the incumbent. When the two safety thresholds are closer together, the new company needs more data and in fact needs to scale up their dataset size at a faster rate: that is, $N_{E}^{*}=\Theta \left(D^{-c}\right)$, where D measures the difference between the safety thresholds and where the exponent c increases as D decreases (*Theorem* 5; Fig. 4). For the parameter settings in ref. 9, the exponent c changes from −2.94 to −3.94 to an even larger value as D decreases. In general, the exponent c takes on up to three different values.

## Technical Tool: Scaling Laws.

To prove our results, we derive scaling laws for multi-objective high-dimensional linear regression, which could be of independent interest (Fig. 2). We study optimally regularized ridge regression where some of the training data is labeled according to the primary linear objective (capturing performance) and the rest is labeled according to an alternate linear objective (capturing safety).

We characterize data-scaling laws for both the loss along the primary objective and the excess loss along the primary objective relative to an infinite-data ridgeless regression. Our scaling laws quantify the rate at which the loss (*Theorem* 2; Fig. 2*A*) decays with the dataset size N, and how this rate is affected by the fraction of data labeled according to each objective and other problem-specific quantities. We also perform a similar analysis for the excess loss (*Theorem* 3; Fig. 2*B*), which subtracts out the loss of the infinite-data ridgeless predictor. Our analysis improves upon recent works on scaling in multi-objective environments (e.g., refs. 10 and 11) by allowing for non-identity covariances and problem-specific regularization, which leads to insights about scaling laws as we describe below.

Our results reveal that the scaling rate becomes slower as the dataset size increases, illustrating that multi-objective scaling laws behave qualitatively differently from classical single-objective environments. While a typical scaling exponent in a single-objective environment takes on a single value across all settings of N, the scaling exponent for multi-objective environments decreases as N increases. In particular, the scaling exponent takes on three different values depending on the size of N relative to problem-specific parameters. The intuition is that the regularizer must be carefully tuned to N in order to avoid overfitting to training data labeled according to the alternate objective, which in turn results in the scaling exponent being dependent on N.

## Discussion.

Altogether, our work highlights the importance of looking beyond model performance when evaluating market entry in machine learning marketplaces. Our results highlight a disconnect between market entry in single-objective environments versus more realistic multi-objective environments. More broadly, a company’s susceptibility to reputational damage affects how they train their model to balance between different objectives. As we discuss in the last section, these insights have nuanced implications for regulators who wish to promote both market competitiveness and safety compliance, and could potentially generalize beyond language models to online platforms.

## Related Work

Our work connects to research threads on competition between companies as well as scaling laws and high-dimensional linear regression.

### Competition Between Model Providers.

Our work contributes to an emerging line of work that studies how competing companies (i.e., model providers) strategically design their machine learning pipelines to attract users. Company actions range from choosing a function from a model class (12–14), to selecting a regularization parameter (15), to choosing an error distribution over user losses (16), to making data purchase decisions (17, 18), to deciding whether to share data (19), to selecting a bandit algorithm (20, 21). While these works assume that companies win users solely by maximizing (individual-level or population-level) accuracy, our framework incorporates the role of safety violations in impacting user retention implicitly via reputational damage. Moreover, our focus is on quantifying the barriers to market entry, rather than analyzing user welfare or the equilibrium decisions of companies.

Other related work includes the study of competition between algorithms (22, 23), retraining dynamics under user participation decisions (24–28), the bargaining game between a foundation model company and a specialist (29), and the market power of an algorithmic platform to shape user populations (30–32).

Our work also relates to platform competition (33, 34), the emerging area of competition policy and regulation of digital marketplaces (1, 35–37), the study of how antitrust policy impacts innovation in classical markets (38, 39), and industrial organization more broadly (40). For example, recent work examines how increased public scrutiny from inclusion in the S&P 500 can harm firm performance (41), how privacy regulation impacts firm competition (42, 43), how regulatory inspections affect incentives to comply with safety constraints (44, 45), and how data-driven network effects can reduce innovation (46).

### Scaling Laws and High-Dimensional Linear Regression.

Our work also contributes to an emerging line of work on scaling laws which study how model performance changes with training resources. Empirical studies have demonstrated that increases to scale often reliably improve model performance (e.g., refs. 2, 9, and 47), but have also identified settings where scaling behavior is more nuanced (e.g., refs. 48 and 49). We build on a recent mathematical characterization of scaling laws based on high-dimensional linear regression (e.g., refs. 50–61). However, while these works focus on single-objective environments where all of the training data is labeled with outputs from a single predictor, we consider multi-objective environments where some fraction of the training data is labeled according to an alternate predictor.

We note that a handful of recent works similarly move beyond single-objective environments and study scaling laws where the training data comes from a mixture of different data sources. Some work (10, 11) studies high-dimensional ridge regression in a similar multi-objective environment to our setup. However, these results assume an identity covariance and focus on fixed regularization or no regularization. In contrast, we allow for covariance matrices that satisfy natural power law decay assumptions (which are not satisfied by the identity matrix), and we also analyze optimally tuned regularization. Our analysis of these problem settings yields new insights about scaling behavior: For example, the scaling rate becomes slower with dataset size (*Theorems* 2–3). Other related works study scaling laws under mixtures of covariate distributions (62), under data-quality heterogeneity (63), under data addition (64), under mixtures of AI-generated data and real data (65, 66), and with respect to the contribution of individual data points (67). These works focus on different technical setups than our work. Specifically, the theoretical analyses of linear regression in refs. 62 and 67 assume that the data sources are labeled according to the same objective, the theoretical analyses in refs. 65 and 66 assume that new data are generated by the current model and replaces or are added to the training dataset over rounds of interactions, and the analyses of scaling trends in refs. 63 and 64 are empirical.

More broadly, our work relates to collaborative learning (68–71), federated learning (see ref. 72 for a survey), optimizing data mixtures (e.g., refs. 73 and 74), and adversarial robustness (e.g., ref. 75). Finally, our work relates to nonmonotone scaling laws in strategic environments (21, 76), where increases to scale can worsen equilibrium social welfare.

## Model

We define our linear-regression-based marketplace, justify the design choices of our model, and then delineate our statistical assumptions.

### Linear Regression-Based Marketplace.

We consider a marketplace where two companies fit linear regression models in a multi-objective environment capturing both performance and safety. A company faces reputational damage if its loss along safety exceeds a company-specific threshold. Each company curates its training data to maximize performance while avoiding reputational damage (i.e., while satisfying its safety constraint). The company then performs (unconstrained) ridge regression on its training data. Our focus is on the market entry threshold which captures how much training data the new company needs to achieve better performance than the incumbent, subject to these safety requirements.

#### Linear regression model.

To formalize each company’s machine learning pipeline, we consider the multi-objective, high-dimensional linear regression model described below. This multi-objective environment aims to capture how ML models are often trained to balance multiple objectives which are in tension with each other, and we consider linear regression since it has often accurately predicted scaling trends of large-scale machine learning models.

More concretely, given an input $x\in R^{P}$, let $\left\langle \beta_{1},x\right\rangle$ be the output that targets performance maximization, and let $\left\langle \beta_{2},x\right\rangle$ be the output that targets safety maximization. Given a linear predictor β, the performance loss is evaluated via a population loss, $L_{1}\left(\beta \right)=E_{x∼D_{F}}\left[{\left(\left\langle \beta_{1},x\right\rangle -\left\langle \beta ,x\right\rangle \right)}^{2}\right]$, and the safety violation is captured by a loss $L_{2}\left(\beta \right)=E_{x∼D_{F}}\left[{\left(\left\langle \beta_{2},x\right\rangle -\left\langle \beta ,x\right\rangle \right)}^{2}\right]$, where $D_{F}$ is the input distribution.

The company implicitly determines how to balance $\beta_{1}$ and $\beta_{2}$ when determining how to label their training dataset. In particular, each company is given an unlabeled training dataset $X\in R^{N\times P}$ with N inputs drawn from $D_{F}$. To generate labels, they select the fraction α∈[0,1] of training data to label according to each objective. They then sample a fraction α of the training data uniformly from X and label it as $Y_{i}=\left\langle \beta_{1},X_{i}\right\rangle$; the remaining 1−α fraction is labeled as $Y_{i}=\left\langle \beta_{2},X_{i}\right\rangle$. The company fits a ridge regression on the labeled training dataset with least-squares loss $\ell \left(y,y^{\prime }\right)={\left(y-y^{\prime }\right)}^{2}$, and thus solves: $\hat{\beta }\left(\alpha ,\lambda ,X\right)={\mathrm{argmin}\;}_{\beta }\left(\frac{1}{N}\sum_{i=1}^{N}{\left(Y_{i}-\left\langle \beta ,X_{i}\right\rangle \right)}^{2}+{\lambda ||\beta ||}_{2}^{2}\right)$.

#### Marketplace.

The marketplace contains two companies, an incumbent company I already in the market and a new (entrant) company E trying to enter the market. At a high level, each company $C\in \left\{I,E\right\}$ faces reputational damage if their safety violation exceeds their safety constraint $\tau_{C}$. Each company C is given $N_{C}$ unlabeled data points sampled from $D_{F}$, and selects a mixture parameter $\alpha_{C}$ and regularizer $\lambda_{C}$ to maximize their performance given their safety constraint $\tau_{C}$. We assume that the incumbent company I faces a stricter safety constraint, $\tau_{I}<\tau_{E}$, due to increased public or regulatory scrutiny.

When formalizing how the companies choose hyperparameters, we make the following simplifications. First, rather than work directly with the performance and safety losses of the ridge regression estimator, we assume for analytic tractability that they approximate these losses by $L_{1}^{*}:=\;L_{1}^{*}\left(\beta_{1},\beta_{2},D_{F},\lambda ,N,\alpha \right)$ and $L_{2}^{*}:=\;L_{2}^{*}\left(\beta_{1},\beta_{2},D_{F},\alpha \right)$ defined as follows.

- Performance: We define $L_{1}^{*}$ to be a deterministic equivalent $L_{1}^{\text{det}}\left(\beta_{1},\beta_{2},\Sigma ,\lambda ,N,\alpha \right)$ which we derive in *SI Appendix*. The deterministic equivalent (cf. 77) is a tool from random matrix theory that is closely linked to the Marčenko–Pastur law (78). Under standard random matrix assumptions (*Assumption* 1), the deterministic equivalent asymptotically approximates the loss $L_{1}\left(\hat{\beta }\left(\alpha ,\lambda ,X\right)\right)$ when X is constructed from N i.i.d. samples from $D_{F}$.
- Safety: For analytic simplicity, in the main body of the paper, we define $L_{2}^{*}$ to be the safety violation of the infinite-data ridgeless regression estimator with mixture parameter α. For this specification, the dataset size N and the regularization parameter λ only affect $L_{1}^{*}$ and not $L_{2}^{*}$, which simplifies our analysis and enables us to obtain tight characterizations. In *SI Appendix*, we instead define $L_{2}^{*}$ analogously to $L_{1}^{*}$—i.e., as a deterministic equivalent $L_{2}^{\text{det}}\left(\beta_{1},\beta_{2},D_{F},\lambda ,N,\alpha \right)$—and extend our model and results to this more complex setting.

Second, we assume that $\left(\beta_{1},\beta_{2}\right)∼D_{W}$ for some joint distribution $D_{W}$ and that the companies take expectations when choosing hyperparameters, since it will be easier to specify our statistical assumptions over distributions of predictors.

Within this setup, a company C faces reputational damage if the safety violation exceeds a certain threshold:

$$\begin{array}{c} E_{\left(\beta_{1},\beta_{2}\right)∼D_{W}}\left[L_{2}^{*}\left(\beta_{1},\beta_{2},D_{F},\alpha_{C}\right)\right]>\tau_{C}. \end{array}$$

We assume that the safety thresholds for the two companies satisfy the following inequalities:

[1] $$\begin{array}{c} \tau_{E}{>}_{\left(A\right)}\tau_{I}\geq_{\left(B\right)}E_{\left(\beta_{1},\beta_{2}\right)∼D_{W}}\left[L_{2}^{*}\left(\beta_{1},\beta_{2},D_{F},0.5\right)\right]. \end{array}$$

Here, inequality (A) captures the notion that the incumbent needs to achieve higher safety to avoid reputational damage. Inequality (B) guarantees that both companies, $C\in \left\{I,E\right\}$, can set the mixture parameter $\alpha_{C}\geq 0.5$ without facing reputational damage, and thus ensures that the safety constraint does not dominate the company’s optimization task.^*^

The company selects $\alpha_{C}\in \left[0.5,1\right]$ and $\lambda_{C}\in \left(0,1\right)$ to maximize their performance subject to their safety constraint, as formalized by the following optimization program:^†^

$$\begin{array}{cc} {\mathrm{argmin}\;}_{\alpha \in [0.5,1],\lambda \in (0,1)} & E_{D_{W}}\left[L_{1}^{*}\left(\beta_{1},\beta_{2},D_{F},\lambda ,N_{C},\alpha \right)\right]\\ & \text{s.t.}E_{D_{W}}\left[L_{2}^{*}\left(\beta_{1},\beta_{2},D_{F},\alpha \right)\right]\leq \tau_{C}. \end{array}$$

#### Market-entry threshold.

We define the market-entry threshold to capture the minimum number of data points $N_{E}$ that the new company needs to collect to achieve better performance than the incumbent company while avoiding reputational damage.

Definition 1 The market-entry threshold $N_{E}^{*}\left(N_{I},\tau_{I},\tau_{E},D_{W},D_{F}\right)$ is the minimum value of $N_{E}\in Z_{\geq 1}$ such that $E_{D_{W}}\left[L_{1}^{*}\left(\beta_{1},\beta_{2},D_{F},\lambda_{E},N_{E},\alpha_{E}\right)\right]\leq E_{D_{W}}\left[L_{1}^{*}\left(\beta_{1},\beta_{2},D_{F},\lambda_{I},N_{I},\alpha_{I}\right)\right]$.

The market entry threshold formalizes how much data the new company needs to attract users and thus enter the market. This definition implicitly makes the following simplifying assumptions about the underlying market. First, we assume that users are homogeneous and are interested in the same distribution $D_{F}$ of queries. Second, we assume that as long as the safety threshold is met, users only care about performance and choose the model that maximizes performance. Finally, we assume that companies cannot compete on prices and only compete on model performance. We revisit these assumptions at the end of the paper (p. 9–10).

The goal of our work is to analyze the function $N_{E}^{*}\left(N_{I},\tau_{I},\tau_{E},D_{W},D_{F}\right)$.

### Model Discussion.

Now that we have formalized our statistical model, we discuss and justify our design choices in greater detail.

#### Presence of competing objectives.

Our multi-objective formulation is motivated by how language models are often trained to balance multiple objectives which are in tension with each other. In some cases, the pretraining objective is in tension with the fine-tuning objective (7). For example, the fine-tuning of a language model to be more aligned with user preferences can degrade performance—e.g., because the model hedges too much—which creates an “alignment tax” (79). In other cases, fine-tuning approaches themselves balance multiple objectives such as helpfulness (which can be mapped to performance in our model) and harmlessness (which can be mapped to safety in our model) (80). These objectives can be in tension with one another, for example if the user asks for dangerous information.

Our formulation takes a data-centric perspective on multi-objective learning. Specifically, we model the company as implicitly determining the weight on each objective by changing the mixture α of training data labeled according to each objective. This design choice is motivated by how data curation—in particular, deciding how to mix data coming from different sources—is a central component of language model training (74). Moreover, this design choice is further motivated by how data annotation is often costly (81), which suggests that companies may have to distribute a limited budget for data annotation between the two objectives.

#### High-dimensional linear regression as a statistical model.

We focus on high-dimensional linear regression due to its ability to capture scaling trends observed in large-scale machine learning models such as language models, while still retaining analytic tractability. In particular, in single-objective environments, scaling trends for overparameterized high-dimensional linear regression (even without label noise) recover the empirically observed power-law scaling of the loss with respect to the dataset size (2, 53, 54). Moreover, overparameterized high-dimensional regression can further capture the scaling rate of empirically observed scaling trends (2, 53, 54). Specifically, this statistical model can exhibit scaling trends of the form $N^{-\nu }$ for ν<0.5—a phenomenon which is not captured by classical underparameterized statistical models. Moreover, from an analytic perspective, the structural properties of high-dimensional linear regression make it possible to characterize the loss using random matrix machinery.

While single-objective high-dimensional regression captures key aspects of empirical scaling trends, this still leaves open the question of whether multi-objective high-dimensional regression faithfully capture multi-objective scaling trends for language models. We defer the empirical comparison of these multi-objective scaling trends to future work.

#### Impact of market position on company constraint *τ*.

Our assumption that $\tau_{E}>\tau_{I}$ (inequality (A) in Eq. **1**) is motivated by how large companies face greater reputational damage from safety violations than smaller companies. One driver of this unevenness in reputational damage is regulation: For example, recent regulation and policy (4, 8) places stricter requirements on companies that use significant amounts of compute during training. In particular, these companies face more stringent compliance requirements in terms of safety assessments and postdeployment monitoring. Another driver of uneven reputational damage is public perception: We expect that the public is more likely to uncover safety violations for large companies, due to the large volume of user queries to the model. In contrast, for small companies, safety violations may be undetected or subject to less public scrutiny.

### Statistical Assumptions on Linear Regression Problem.

To simplify our characterization of scaling trends, we follow prior work on high-dimensional linear regression (see, e.g., refs. 53 and 54) and make the following empirically motivated power-law assumptions. Let $\Sigma =E_{x∼D_{F}}\left[xx^{T}\right]$ be the covariance matrix, and let $\lambda_{i}$ and $v_{i}$ be the eigenvalues and eigenvectors, respectively.

We require the eigenvalues to decay with scaling exponent γ>0 according to $\lambda_{i}=i^{-1-\gamma }$ for 1≤i≤P. The eigenvalues capture the variance along each eigendirection. A high value of γ thus means that a small number of directions capture the data’s variability, whereas a low value of γ means that many directions are needed to accurately capture the data.

For the alignment coefficients $\left\langle \beta_{j},v_{i}\right\rangle$, it is cleaner to enforce power scaling assumptions in expectation over a joint distribution $\left(\beta_{1},\beta_{2}\right)∼D_{W}$, since this enables us to more easily define a correlation parameter ρ which captures the extent to which the $\beta_{1}$ and $\beta_{2}$ are aligned with each other. We require that for some δ>0, the alignment coefficients satisfy $E_{D_{W}}\left[{\left\langle \beta_{j},v_{i}\right\rangle }^{2}\right]=i^{-\delta }$, where $v_{i}$ is the ith eigenvector of Σ, for $j\in \left\{1,2\right\}$ and 1≤i≤P. We also introduce a similar condition on the joint alignment coefficients, requiring that for some ρ∈[0,1), it holds that $E_{D_{W}}\left[\left\langle \beta_{1},v_{i}\right\rangle \left\langle \beta_{2},v_{i}\right\rangle \right]=\rho \cdot i^{-\delta }$. The scaling exponent δ captures how easy it is to capture the ground truth predictors $\beta_{1}$ and $\beta_{2}$ in the eigenbasis of the covariance matrix. A higher value of δ means that a smaller number of eigendimensions are needed to capture the predictors.

Below, we provide an example which satisfies these assumptions.

Example 1 Suppose that the covariance Σ is a diagonal matrix with diagonal given by $\lambda_{i}=i^{-1-\gamma }$. Let the joint distribution $D_{W}$ over $\beta_{1}$ and $\beta_{2}$ be a multivariate Gaussian such that $E_{D_{W}}\left[{\left(\beta_{j_{1}}\right)}_{i_{1}}{\left(\beta_{j_{2}}\right)}_{i_{2}}\right]$ equals

$$\left\{\begin{array}{cc} 0 & \text{if}\;1\leq j_{1},j_{2}\leq 2,1\leq i_{1}\neq i_{2}\leq P\\ i_{1}^{-\delta } & \text{if}\;1\leq j_{1}=j_{2}\leq 2,1\leq i_{1}=i_{2}\leq P\\ \rho \cdot i_{1}^{-\delta } & \text{if}\;1\leq j_{1}\neq j_{2}\leq 2,1\leq i_{1}=i_{2}\leq P. \end{array}\right.$$

This implies $E_{D_{W}}\left[{\left\langle \beta_{j},v_{i}\right\rangle }^{2}\right]=i^{-\delta }$ and $E_{D_{W}}\left[\left\langle \beta_{1},v_{i}\right\rangle \left\langle \beta_{2},v_{i}\right\rangle \right]=\rho \cdot i^{-\delta }$. The correlation ρ captures the extent to which $\beta_{1}$ and $\beta_{2}$ are aligned with each other as opposed to in tension.

The scaling exponents δ and γ both emerge in scaling law characterizations (*Theorems* 2 and 3): Increasing δ and γ improves the data efficiency of learning.

We adopt standard random matrix theory assumptions (55) on the covariance matrix and linear predictors, which guarantee that the Marčenko–Pastur law holds (78). That is, the covariance ${\left(\hat{\Sigma }+\lambda I\right)}^{-1}$ of the samples can be approximated by a deterministic quantity. We leverage this Marenko–Pastur law to derive a deterministic equivalent $L_{1}^{\text{det}}$ for the performance loss $L_{1}\left(\hat{\beta }\left(\alpha ,\lambda ,X\right)\right)$ of the ridge regression estimator, as we formalize in *SI Appendix*.

More specifically, we require the following structural assumptions on number of data points N≥1, the number of parameters P≥1, the distribution $D_{F}$, and the vectors $\beta_{1}$ and $\beta_{2}$. We adopt assumptions from ref. 55 which guarantee that a Marčenko–Pastur law holds for Σ, and we further introduce a boundedness assumption for technical reasons.

Assumption 1 *We assume that (1)* $X∼D_{F}$ *takes the form* $X=Z\Sigma^{1/2}$, *where* Z *has bounded sub-Gaussian i.i.d components with mean zero and unit variance, (2)* N *and* P *approach* ∞ *with* $\frac{P}{N}$ *tending to* Γ∈(0,∞), *(3) the spectral measure* $\frac{1}{P}\sum_{i=1}^{P}\Delta_{\lambda_{i}}$ *of* Σ *converges to a probability measure with compact support*,^‡^ *and* Σ *is invertible and bounded in operator norm, and (4) for* $j\in \left\{1,2\right\}$, *the measure* $\sum_{i=1}^{P}{\left\langle v_{i},\beta_{j}\right\rangle }^{2}$ *converges to a measure with bounded mass, and* $\beta_{j}$ *has bounded* $\ell_{2}$ *norm*.

In our scaling law characterizations (*Theorems* 2 and 3), we assume an overparameterized limit where the number of parameters P→∞ approaches infinity.^§^

## Warm Up: Infinite-Data Incumbent and Unconstrained Entrant

As a warmup, we analyze the market entry $N_{E}^{*}$ threshold in a simplified environment where the incumbent has infinite data and the new company faces no safety constraint. In this result, we place power-law scaling assumptions on the covariance and alignment coefficients, and we characterize the threshold $N_{E}^{*}$ up to constants (*Theorem* 1; Fig. 1).

Theorem 1 *Suppose that the power-law scaling assumptions hold with exponents* γ,δ>0 *and correlation coefficient* ρ∈[0,1), *and suppose that* P=∞. *Suppose that the incumbent company has infinite data (i.e.*, $N_{I}=\infty$), *and that the entrant faces no constraint on their safety (i.e.*, $\tau_{E}=\infty$). *Suppose that the safety constraint* $\tau_{I}$ *satisfies Eq.* **1**. *Then, it holds that the market entry threshold* $N_{E}^{*}=N_{E}^{*}\left(\infty ,\tau_{I},\infty ,D_{W},D_{F}\right)$ *satisfies*

$$\begin{array}{c} N_{E}^{*}=\Theta \left({\left(\sqrt{L^{*}\left(\rho \right)}-\sqrt{\min(\tau_{I},L^{*}\left(\rho \right))}\right)}^{-2/\nu }\right), \end{array}$$

*where* $L^{*}\left(\rho \right)=E_{D_{W}}\left[{\left(\beta_{1}-\beta_{2}\right)}^{T}\Sigma \left(\beta_{1}-\beta_{2}\right)\right]=\Theta \left(1-\rho \right)$, *and where* ν:=min(2(1+γ),δ+γ).

The intuition is as follows. The safety constraint $\tau_{I}$ forces the incumbent company to partially align their predictor with the safety objective $\beta_{2}$. Since $\beta_{1}$ and $\beta_{2}$ point in different directions, this reduces the performance of the incumbent along $\beta_{1}$ as a side effect, resulting in strictly positive loss with respect to performance. On the other hand, since the new company faces no safety constraint, the new company can optimize entirely for performance along $\beta_{1}$. This means that the new company can enter the market as long as their finite-data error is bounded by the incumbent’s performance loss. We formalize this intuition in the following proof sketch.

**Proof** The incumbent chooses the infinite-data ridgeless estimator $\beta (\alpha_{I},0)$ with mixture parameter $\alpha_{I}\in \left[0,1\right]$ tuned so the safety violation is $\tau_{I}$ and with regularizer $\lambda_{I}$ set to be 0. The resulting performance loss is $\sqrt{L^{*}\left(\rho \right)}-\sqrt{\min(\tau_{I},L^{*}\left(\rho \right))}$. Since the new company has no safety constraint, they choose the single-objective ridge regression estimator where $\alpha_{E}=1$ and where $\lambda_{E}$ is chosen optimally.^¶^*Theorem* 2 (or alternatively, existing analyses of high-dimensional linear regression (e.g., refs. 53 and 54) demonstrate the loss follows a scaling law of the form $\underset{\lambda >0}{\inf}L_{1}\left(\hat{\beta }\left(1,\lambda ,X\right)\right)=\Theta \left(N^{-\nu }\right)$, where ν:=min(2(1+γ),δ+γ).

*Theorem* 1 reveals that the market-entry threshold is finite as long as 1) the safety constraint $\tau_{I}$ places nontrivial restrictions on the incumbent company and 2) the safety and performance objectives are not perfectly correlated. This result captures the notion that the new company can enter the market even after the incumbent company has accumulated an infinite amount of data.

*Theorem* 1 further illustrates how the market-entry threshold changes with other parameters (Fig. 1). When safety and performance objectives are more correlated (i.e., when ρ is higher), the market-entry threshold increases, which increases barriers to entry. When the safety constraint for the incumbent is weaker (i.e., when $\tau_{I}$ is higher), the market-entry threshold also increases. Finally, when the power scaling parameters of the covariance and alignment coefficients increase, which increases the scaling law exponent ν, the market-entry threshold decreases.

## Generalized Analysis of the Market-Entry Threshold

To obtain a more fine-grained characterization of the market-entry threshold, we now consider more general environments. Our key technical tool is multi-objective scaling laws, which capture the performance of ridge regression in high-dimensional, multi-objective environments with finite data. Using these scaling laws, we characterize the market-entry threshold when the incumbent has finite data and when the new company has a safety constraint.

Our results in this section uncover the following conceptual insights about market entry. First, our main finding from the previous section—that the new company can enter the market with significantly less data than the incumbent—applies in many cases to these generalized environments. Moreover, our characterizations of $N_{E}^{*}$ exhibit a power-law-like dependence with respect to the incumbent’s dataset size (*Theorem* 4) and the difference in safety requirements for the two companies (*Theorem* 5). Interestingly, the scaling exponent is not a constant across the full regime and instead takes on up to three different values. As a consequence, the new company can afford to scale up their dataset at a slower rate as the incumbent’s dataset size increases, but needs to scale up their dataset at a faster rate as the two safety constraints become closer together.

### Technical Tool: Scaling Laws in Multi-objective Environments.

In this section, we give an overview of multi-objective scaling laws (see the next section for a more formal treatment and derivations). Our scaling laws capture how the ridge regression loss $L_{1}\left(\hat{\beta }\left(\alpha ,\lambda ,X\right)\right)$ along the primary objective $\beta_{1}$ scales with the dataset size N, when the regularizer λ is optimally tuned to both N and problem-specified parameters. We show scaling laws for both the loss $\underset{\lambda \in (0,1)}{\inf}E\left[L_{1}\left(\hat{\beta }\left(\alpha ,\lambda ,X\right)\right)\right]$ and the excess loss $\underset{\lambda \in (0,1)}{\inf}\left(E\left[L_{1}\left(\hat{\beta }\left(\alpha ,\lambda ,X\right)\right)-L_{1}\left(\beta \left(\alpha ,0\right)\right)\right]\right)$, where β(α,0) is the infinite-data ridgeless regression estimator.

#### Scaling law for the loss.

We first describe the scaling law for $\underset{\lambda \in (0,1)}{\inf}E\left[L_{1}\left(\hat{\beta }\left(\alpha ,\lambda ,X\right)\right)\right]$ (*Theorem* 2; Fig. 2*A*). We present an informal version here, deferring the formal version to *SI Appendix*.

Theorem 2 *Suppose that the power-law scaling assumptions hold with exponents* γ,δ>0 *and correlation coefficient* ρ∈[0,1). *Suppose also that* P=∞ *and* α≥0.5. *Then, a deterministic equivalent for the expected loss under optimal regularization* $\underset{\lambda \in (0,1)}{\inf}E\left[L_{1}\left(\hat{\beta }\left(\alpha ,\lambda ,X\right)\right)\right]$ *scales according to* $N^{-\nu^{*}}$, *where the scaling exponent* $\nu^{*}$ *is defined to be*

$$\left\{\begin{array}{cc} \nu & \text{if}\;\;N\leq {\left(1-\alpha \right)}^{-\frac{1}{\nu }}{\left(1-\rho \right)}^{-\frac{1}{\nu }}\\ \frac{\nu }{\nu +1} & \text{if}\;\;{\left(1-\alpha \right)}^{-\frac{1}{\nu }}{\left(1-\rho \right)}^{-\frac{1}{\nu }}\leq N\leq {\left(1-\alpha \right)}^{-\frac{2+\nu }{\nu }}{\left(1-\rho \right)}^{-\frac{1}{\nu }}\\ 0 & \text{if}\;\;N\geq {\left(1-\alpha \right)}^{-\frac{2+\nu }{\nu }}{\left(1-\rho \right)}^{-\frac{1}{\nu }}, \end{array}\right.$$

*for* ν:=min(2(1+γ),δ+γ).

*Theorem* 2 (Fig. 2*A*) illustrates that the scaling rate becomes slower as the dataset size N increases. In particular, while the scaling exponent in single-objective environments is captured by a single value, *Theorem* 2 illustrates that the scaling exponent $\nu^{*}$ in multi-objective environments takes on three different values, depending on the size of N relative to other parameters. When N is small (the first regime), the scaling exponent $\nu^{*}=\nu$ is identical to that of the single-objective environment given by $\beta_{1}$. When N is a bit larger (the second regime), the scaling exponent reduces to $\nu^{*}=\nu /\left(\nu +1\right)<\nu$. To make this concrete, if we take ν=0.34 to be an empirically estimated scaling law exponent for language models (9), this would mean that $\nu^{*}\approx 0.34$ in the first regime and $\nu^{*}\approx 0.25$ in the second regime. Finally, when N is sufficiently large (the third regime), the scaling exponent reduces all the way to $\nu^{*}=0$ and the only benefit of additional data is to improve constants on the loss.

This decrease in the scaling exponent ν comes from how the optimal regulation level changes with the dataset size N. Specifically, when N is sufficiently large (i.e., in the second and third regimes in *Theorem* 2), the optimal loss is not necessarily achieved by taking λ→0. In fact, if the regularizer decays too quickly as a function of N (i.e., if $\lambda =o(N^{-1-\gamma })$), then the loss would converge to a factor of ${\left(1-\alpha \right)}^{-1}$ higher than the loss of the infinite-data ridgeless predictor β(α,0). The fact that λ→0 is suboptimal reveals a sharp disconnect between the multi-objective setting and the single-objective setting where no explicit regularization is necessary to achieve the optimal loss (see, e.g., refs. 53 and 54). As we show in *SI Appendix*, as N gets larger, the increasing need for careful regularization decreases the data efficiency of learning.

#### Scaling law for the excess loss.

We next turn to the excess loss, $\underset{\lambda \in (0,1)}{\inf}\left(E\left[L_{1}\left(\hat{\beta }\left(\alpha ,\lambda ,X\right)\right)-L_{1}\left(\beta \left(\alpha ,0\right)\right)\right]\right)$, which is normalized by the loss of the infinite-data ridgeless predictor β(α,0). We show that the excess loss exhibits the same scaling behavior as the loss when N is sufficiently small, but exhibits different behavior when N is sufficiently large (*Theorem* 3; Fig. 2*B*). We present an informal version here, deferring the formal version to *SI Appendix*.

Theorem 3 *Suppose that the power-law scaling assumptions hold with exponents* γ,δ>0 *and correlation coefficient* ρ∈[0,1). *Suppose also that* P=∞ *and* α≥0.75. *Then, a deterministic equivalent for the expected loss under optimal regularization* $\underset{\lambda \in (0,1)}{\inf}\left(E\left[L_{1}\left(\hat{\beta }\left(\alpha ,\lambda ,X\right)\right)-L_{1}\left(\beta \left(\alpha ,0\right)\right)\right]\right)$ *scales according to* $N^{-\nu^{*}}$, *where the scaling exponent* $\nu^{*}$ *is defined to be*

$$\left\{\begin{array}{cc} \nu & \text{if}\;\;N\leq {\left(1-\alpha \right)}^{-\frac{1}{\nu }}{\left(1-\rho \right)}^{-\frac{1}{\nu }}\\ \frac{\nu }{\nu +1} & \text{if}\;\;{\left(1-\alpha \right)}^{-\frac{1}{\nu }}{\left(1-\rho \right)}^{-\frac{1}{\nu }}\leq N\leq {\left(1-\alpha \right)}^{-\frac{\nu^{\prime }+1}{\nu -\nu^{\prime }}}{\left(1-\rho \right)}^{-\frac{\nu^{\prime }+1}{\nu -\nu^{\prime }}}\\ \frac{\nu^{\prime }}{\nu^{\prime }+1} & \text{if}\;\;N\geq {\left(1-\alpha \right)}^{-\frac{\nu^{\prime }+1}{\nu -\nu^{\prime }}}{\left(1-\rho \right)}^{-\frac{\nu^{\prime }+1}{\nu -\nu^{\prime }}}, \end{array}\right.$$

*for* ν:=min(2(1+γ),δ+γ) *and* $\nu^{\prime }:=\;\min\left(1+\gamma ,\delta +\gamma \right)$.

*Theorem* 3 (Fig. 2*B*) again shows that the scaling rate can become slower as the dataset size N increases, and again reveals three regimes of scaling behavior. While the first two regimes of *Theorem* 3 resemble the first two regimes of *Theorem* 2, the third regime of *Theorem* 3 (where $N\geq {\left(1-\alpha \right)}^{-\frac{\nu^{\prime }+1}{\nu -\nu^{\prime }}}{\left(1-\rho \right)}^{-\frac{\nu^{\prime }+1}{\nu -\nu^{\prime }}}$) behaves differently. In this regime, the scaling exponent for the excess loss is $\frac{\nu^{\prime }}{\nu^{\prime }+1}$, rather than zero—this captures the fact that additional data can nontrivially improve the excess loss even in this regime, even though it only improves the loss up to constants. In terms of the magnitude of the scaling exponent $\frac{\nu^{\prime }}{\nu^{\prime }+1}$, it is strictly smaller than the scaling exponent $\frac{\nu }{\nu +1}$ when δ>1 and equal to the scaling exponent $\frac{\nu }{\nu +1}$ when δ≤1.

As in *Theorem* 3, the fact that the scaling exponent ν is decreasing in N comes from how the optimal regulation level changes with the dataset size N.

### Finite Data for the Incumbent.

We compute $N_{E}^{*}$ when the incumbent has finite data and the new company has no safety constraint (*Theorem* 4; Fig. 3). The market-entry threshold $N_{E}^{*}$ depends on the incumbent’s dataset size $N_{I}$, the incumbent’s performance loss $G_{I}$ if they were to have infinite data but face the same safety constraint, the scaling exponents γ,δ, and the correlation coefficient ρ.

Theorem 4 *Suppose that the power-law scaling assumptions hold with exponents* γ,δ>0 *and correlation coefficient* ρ∈[0,1), *and suppose that* P=∞. *Assume that* $\tau_{E}=\infty$. *Suppose that the safety constraint* $\tau_{I}$ *satisfies Eq. **1**. Then we have that the market entry threshold* $N_{E}^{*}=N_{E}^{*}\left(N_{I},\tau_{I},\infty ,D_{W},D_{F}\right)$ *equals*:

$$\left\{\begin{array}{cc} \Theta (N_{I}) & \text{if}\;N_{I}\leq {\left(G_{I}\left(1-\rho \right)\right)}^{-\frac{1}{2\nu }}\\ \Theta (N_{I}^{\frac{1}{\nu +1}}\cdot {\left(G_{I}\left(1-\rho \right)\right)}^{-\frac{1}{2(\nu +1)}}) & \text{if}\;N_{I}\geq {\left(G_{I}\left(1-\rho \right)\right)}^{-\frac{1}{2\nu }},\\ & \text{and}\;\;N_{I}\leq G_{I}^{-\frac{1}{2}-\frac{1}{\nu }}{\left(1-\rho \right)}^{\frac{1}{2}}\\ \Theta (G_{I}^{-\frac{1}{\nu }}) & \text{if}\;N_{I}\geq G_{I}^{-\frac{1}{2}-\frac{1}{\nu }}{\left(1-\rho \right)}^{\frac{1}{2}}, \end{array}\right.$$

*where* $L^{*}\left(\rho \right)=E_{D_{W}}\left[{\left(\beta_{1}-\beta_{2}\right)}^{T}\Sigma \left(\beta_{1}-\beta_{2}\right)\right]=\Theta \left(1-\rho \right)$, *where* $G_{I}:=\;{\left(\sqrt{L^{*}\left(\rho \right)}-\sqrt{\min(\tau_{I},L^{*}\left(\rho \right))}\right)}^{2}$, *and where* ν=min(2(1+γ),δ+γ).

The market-entry threshold in *Theorem* 4 exhibits three regimes of behavior depending on $N_{I}$. In particular, the market-entry threshold takes the form $N_{E}^{*}=\Theta \left(N_{I}^{c}\right)$, where c decreases from 1 (in the first regime) to $\frac{1}{\nu +1}$ (in the second regime) to 0 (in the third regime) as $N_{I}$ increases. To connect this to large-language-model marketplaces, we directly set ν=0.34 to be the empirically estimated scaling law exponent for language models (9); in this case, the scaling exponent ranges from 1 to 0.75 to 0. The fact that there are three regimes comes from the scaling law derived in *Theorem* 2, as the following proof sketch illustrates.

**Proof sketch:** The key technical tool is the scaling law for the loss $\underset{\lambda \in (0,1)}{\inf}E\left[L_{1}\left(\hat{\beta }\left(\alpha ,\lambda ,X\right)\right)\right]$ (*Theorem* 2), which has three regimes of scaling behavior for different values of N. We apply the scaling law to analyze the performance of the incumbent, who faces a safety constraint and has finite data. Analyzing the performance of the new company—who faces no safety constraint—is more straightforward, given that the new company can set $\alpha_{E}=1$. We compute $N_{E}^{*}$ as the number of data points needed to match the incumbent’s performance level.

*Theorem* 4 reveals that the new company can enter the market with $N_{E}^{*}=o\left(N_{I}\right)$ data, as long as the incumbent’s dataset is sufficiently large (i.e., $N_{I}\geq G_{I}^{-\frac{1}{2\nu }}{\left(1-\rho \right)}^{-\frac{1}{2\nu }}$). The intuition is that when there are sufficient data, the multi-objective scaling exponent is worse than the single-objective scaling exponent (*Theorem* 2). The incumbent thus faces a worse scaling exponent than the new company, so the new company can enter the market with asymptotically less data.

The three regimes in *Theorem* 4 further reveal that the market-entry threshold $N_{E}^{*}$ scales at a slower rate as the incumbent’s dataset size $N_{I}$ increases (Fig. 3). The intuition is that the multi-objective scaling exponent $\nu^{*}$ faced by the incumbent decreases as dataset size increases, while the single-objective scaling exponent faced by the new company is constant in dataset size (*Theorem* 2). The incumbent thus becomes less efficient at using additional data to improve performance, while the new company’s efficiency in using additional data remains unchanged.

*Theorem* 4 also offers finer-grained insight into the market-entry threshold in each regime. In the first regime, where the incumbent’s dataset is small, the threshold $N_{E}^{*}$ matches the incumbent dataset size. Perhaps counterintuitively, this means that the new company does need as much data as the incumbent to enter the market, even though the new company faces a less stringent safety constraint. In the second (intermediate) regime, the new company can enter with a dataset size proportional to $N_{I}^{1/(\nu +1)}$. This polynomial speedup illustrates that the new company can more efficiently use additional data to improve performance than the incumbent company. A caveat is that this regime is somewhat restricted in that the ratio of the upper and lower boundaries is bounded. In the third regime, where the incumbent’s dataset size is large, the market-entry threshold $N_{E}^{*}$ matches the market-entry threshold from *Theorem* 1 where the incumbent has infinite data.

### Safety Constraint for the New Company.

We compute $N_{E}^{*}$ when the new company has a nontrivial safety constraint and the incumbent has infinite data. For this result, we strengthen the conditions on $\tau_{E}$ and $\tau_{I}$ from Eq. **1**, instead requiring:

[2] $$\begin{array}{c} \tau_{E}{>}_{\left(A\right)}\tau_{I}\geq_{\left(B\right)}E_{\left(\beta_{1},\beta_{2}\right)∼D_{W}}\left[L_{2}^{*}\left(\beta_{1},\beta_{2},D_{F},0.75\right)\right], \end{array}$$

where Eq. **2** replaces the value of 0.5 with a value of 0.75 in the right-most quantity. Inequality (B) in Eq. **2** requires that the safety constraint still allows both companies to label 75% of their training data according to performance-optimal outputs. We make this modification, since our analysis of multi-objective scaling laws for the *excess* loss assumes α≥0.75.

We state the result in the following (*Theorem* 5; Fig. 4). The market-entry threshold $N_{E}^{*}$ depends on the incumbent’s safety constraint $\tau_{I}$, the performance loss $G_{I}$ (resp. $G_{E}$) if the incumbent (resp., the new company) had infinite data and faced the same safety constraint, the difference $D=G_{I}-G_{E}$ in infinite-data performance loss achievable by the incumbent and the new company, the scaling exponents γ,δ, and the correlation coefficient ρ.

Theorem 5 *Suppose that the power-law scaling assumptions hold with exponents* γ,δ>0 *and correlation coefficient* ρ∈[0,1), *and suppose that* P=∞. *Suppose that the safety constraints* $\tau_{I}$ *and* $\tau_{E}$ *satisfy Eq. **2**. Then it holds that the market entry threshold* $N_{E}^{*}=N_{E}^{*}\left(\infty ,\tau_{I},\tau_{E},D_{W},D_{F}\right)$ *equals*

$$\left\{\begin{array}{cc} \Theta (D^{-\frac{1}{\nu }}) & \text{if}\;\;D\geq G_{E}^{\frac{1}{2}}{\left(1-\rho \right)}^{\frac{1}{2}}\\ \Theta (D^{-\frac{\nu +1}{\nu }}G_{E}^{\frac{1}{2}}{\left(1-\rho \right)}^{\frac{1}{2}}) & \text{if}\;\;D\geq G_{E}^{\frac{\nu }{2(\nu -\nu^{\prime })}}{\left(1-\rho \right)}^{\frac{\nu }{2(\nu -\nu^{\prime })}}\\ & \text{and}\;\;D\leq G_{E}^{\frac{1}{2}}{\left(1-\rho \right)}^{\frac{1}{2}}\\ \Theta ((D\cdot G_{E}^{-\frac{1}{2}}{\left(1-\rho \right)}^{-\frac{1}{2}}{)}^{-\frac{\nu^{\prime }+1}{\nu^{\prime }}}) & \text{if}\;\;D\leq G_{E}^{\frac{\nu }{2(\nu -\nu^{\prime })}}{\left(1-\rho \right)}^{\frac{\nu }{2(\nu -\nu^{\prime })}}, \end{array}\right.$$

*where* $L^{*}\left(\rho \right)=E_{D_{W}}\left[{\left(\beta_{1}-\beta_{2}\right)}^{T}\Sigma \left(\beta_{1}-\beta \right)\right]=\Theta \left(1-\rho \right)$, *where* ν=min(2(1+γ),δ+γ) *and* $\nu^{\prime }=\;\min\left(1+\gamma ,\delta +\gamma \right)$, *where* $G_{I}:=\;{\left(\sqrt{L^{*}\left(\rho \right)}-\sqrt{\min(\tau_{I},L^{*}\left(\rho \right))}\right)}^{2}$ *and* $G_{E}:=\;{\left(\sqrt{L^{*}\left(\rho \right)}-\sqrt{\min(\tau_{E},L^{*}\left(\rho \right))}\right)}^{2}$, *and where* $D:=\;G_{I}-G_{E}$.

The market-entry threshold in *Theorem* 5 also exhibits three regimes of behavior depending on the difference D in the infinite-data performance loss achievable by the incumbent and the new company. In particular, the market-entry threshold takes the form $N_{E}^{*}=\Theta \left(D^{-c}\right)$, where c increases from $\frac{1}{\nu }$ to $\frac{\nu +1}{\nu }$ to $\frac{\nu^{\prime }+1}{\nu^{\prime }}$ as D decreases. (The third regime only exists when δ>1.) To connect this to large-language-model marketplaces, if we take ν=0.34 to be the empirically estimated scaling law exponent for language models (9), then c would range from 2.94 to 3.94 to potentially even larger values. The fact that there are three regimes comes from the scaling law derived in *Theorem* 3, as the following proof sketch illustrates.

**Proof sketch:** The key technical tool is the scaling law for the *excess loss*$\underset{\lambda \in (0,1)}{\inf}\left(E\left[L_{1}\left(\hat{\beta }\left(\alpha ,\lambda ,X\right)\right)-L_{1}\left(\beta \left(\alpha ,0\right)\right)\right]\right)$ (*Theorem* 3), which has three regimes of scaling behavior for different values of N. We apply the scaling law to analyze the performance of the new company, who faces a safety constraint and has finite data. Analyzing the performance of the incumbent—who has infinite data—is more straightforward, and the incumbent’s performance loss is $G_{I}=D+G_{E}$. We compute the number of data points $N_{E}^{*}$ needed for the new company to achieve an excess loss of D.

*Theorem* 5 illustrates that the new company can enter the market with finite data $N_{E}^{*}$, as long as the safety constraint $\tau_{E}$ placed on the new company is strictly weaker than the constraint $\tau_{I}$ placed on the incumbent company [inequality (A) in Eq. **2**]. This translates to the difference D being strictly positive. The intuition is that when the new company faces a weaker safety constraint, it can train on a greater number of data points labeled with the performance objective $\beta_{1}$, which improves performance.

The three regimes in *Theorem* 5 further reveal that the market-entry threshold $N_{E}^{*}$ scales at a faster rate as the difference D between the two safety constraints decreases (Fig. 4). This means that as the safety constraints become more even, the new company not only needs more data but also must scale up its dataset at a faster rate. The intuition is that since the new company needs to achieve an excess loss of at most D, the new company faces a smaller multi-objective scaling exponent $\nu^{*}$ as D decreases (*Theorem* 3). This means that as D becomes smaller, the new company becomes less efficient at using additional data to improve performance.

## Discussion

We studied market entry in marketplaces for machine learning models, showing that pressure to satisfy safety constraints can reduce barriers to entry for new companies. We modeled the marketplace using a high-dimensional multi-objective linear regression model. Our key finding was that a new company can consistently enter the marketplace with significantly less data than the incumbent. En route to proving these results, we derive scaling laws for multi-objective regression, showing that the scaling rate becomes slower when the dataset size is large.

### Potential Implications for Regulation.

Our results have nuanced design consequences for regulators, who implicitly influence the level of safety that each company needs to achieve to avoid reputational damage. On the one hand, our results suggest that placing greater scrutiny on dominant companies can encourage market entry and create a more competitive marketplace of companies. On the other hand, market entry does come at a cost to safety. Specifically, the smaller companies exploit the fact that they can incur more safety violations while maintaining their reputation. Unfortunately, this may lead to a race to the bottom for safety. Examining the tradeoffs between market competitiveness and safety compliance, and determining how to balance these tradeoffs, is an important direction for future work.

### Barriers to Market Entry for Online Platforms.

While we focused on language models, we expect that our conceptual findings regarding market entry also extend to recommendation and social media platforms.

In particular, our motivation and modeling assumptions capture key aspects of these online platforms. Policymakers have raised concerns about barriers to entry for social media platforms (35), motivated by the fact that social media platforms such as X and Facebook each have more than a half billion users (82, 83). Incumbent companies risk reputational damage if their model violates safety-oriented objectives—many recommendation platforms have faced scrutiny for promoting hate speech (84), divisive content (85), and excessive use by users (86), even when recommendations perform well in terms of generating user engagement. This means that incumbent platforms must balance optimizing engagement with controlling negative societal impact (87). Moreover, new companies face less regulatory scrutiny, given that some regulations explicitly place more stringent requirements on companies with large user bases. For example, the Digital Services Act (84) places a greater responsibility on Very Large Online Platforms (with over 45 million users per month) to identify and remove illegal or harmful content.

Given that incumbent platforms similarly face more pressure to satisfy safety-oriented objectives, our results suggest that multi-objective learning can also reduce barriers to entry for new online platforms.

### Limitations.

Our model for interactions between companies and users makes several simplifying assumptions. For example, we focused entirely on whether the new company can enter the market, which leaves open the question of whether the new company can survive in the long run. Moreover, we assumed that all users choose the model with the highest overall performance. However, different users may care about performance on different queries, or explicitly value safety rather than only caring about performance subject to the safety threshold being met. We expect that this heterogeneity in user choice could create an incentive for new companies to specialize to specific subpopulations of users, which could further reduce barriers to entry and thus market concentration. Furthermore, we assumed that companies cannot compete on price and only compete on model performance. We expect that while new companies may be able to enter the market by offering lower-quality models at a lower price, it is possible that the incumbent company may compete by also offering lower quality models at a lower price. Finally, we focused on direct interactions between companies and users, but in reality, downstream providers sometimes build services on top of a foundation model. Understanding how these market complexities affect market entry as well as long-term concentration is an interesting direction for future work.

Furthermore, our model also made the simplifying assumption that performance and safety trade off according to a multi-objective regression problem. However, not all safety objectives fit the mold of linear coefficients within linear regression. For some safety objectives such as guaranteeing privacy or ensuring safety on worst-case queries, we still expect that placing greater scrutiny on dominant companies could similarly reduce barriers to entry. Nonetheless, with respect to other safety or societal considerations, we do expect that the implications for market entry might be fundamentally different. For example, if the safety objective is a multigroup performance criteria, and there is a single predictor that achieves zero accuracy on all distributions, then a dominant company with infinite data would be able to retain all users even if the company faces greater scrutiny. Extending our model to capture a broader scope of safety objectives is a natural direction for future work.

Finally, we formulated the company’s learning problem within a multi-objective high-dimensional regression framework. As we discussed earlier, this is motivated by how single-objective high-dimensional regression captures key aspects of scaling trends for language models that have been empirically observed. A natural direction for future work would be to extend this comparison to multi-objective scaling trends and to empirically evaluate how well multi-objective high-dimensional regression captures multi-objective scaling trends for language models. Relatedly, it would also be interesting to consider the robustness of our analysis of the market entry threshold to variations in the multi-objective regression framework: For example, the company could instead choose α and λ to minimize an (unconstrained) weighted sum of the performance loss and safety loss, rather than to minimize the performance loss subject to a constraint on the safety loss. Finally, it would also be interesting to consider the statistical and computational tractability of the company’s optimization of α and λ.

## Supplementary Material

Appendix 01 (PDF)

## Data Availability

There are no data underlying this work.

## Footnotes

^*^More specifically, inequality (B) ensures that the safety constraint still allows both companies to label 50% of their training data according to the performance-optimal outputs.

^†^Technically, the optimum might be achieved at λ=0 or λ=1, and the min should be replaced by an inf.

^‡^We use $\Delta_{x}$ to denote the Dirac measure with parameter x.

^§^To formalize this, we take a limit as Γ→∞. This enables us to study the loss and excess loss as a function of the dataset size N in the limit as P→∞.

^¶^We formally rule out the possibility that $\alpha_{E}\neq 1$ using our multi-objective scaling law in *Theorem* 2.
