# Supplementary material for: Safety versus performance: How multi-objective learning reduces barriers to market entry
Source: Proc Natl Acad Sci U S A. 2025 Oct 15;122(42):e2510004122. doi: 10.1073/pnas.2510004122 (PMC12557473; doi:10.1073/pnas.2510004122)
Supplement: Supplementary file 1 — Appendix 01 (PDF) [file pnas.2510004122.sapp.pdf]

Supporting Information for

Safety vs. Performance: How Multi-Objective Learning Reduces Barriers to Market Entry

Meena Jagadeesan, Michael I. Jordan, Jacob Steinhardt

Michael I. Jordan.
E-mail: jordan@cs.berkeley.edu

This PDF file includes:

Supporting text
SI References

Supporting Information Text

1. Deriving Scaling Laws for Multi-Objective Environments

We formalize and derive our multi-objective scaling laws for the loss (Theorem 2) and excess loss (Theorem 3). Recall that the problem setting is high-dimensional ridge regression when a fraction α of the training data is labelled according to β_1 and the rest is labelled according to an alternate objective β_2. First, following the style of analysis of single-objective ridge regression (e.g., 1, 2), we first compute a *deterministic equivalent* of the loss. Then we derive the scaling law under the power scaling assumptions on the eigenvalues and alignment coefficients, both for the loss (and for the excess loss.

Deterministic equivalent. We show that the loss of the ridge regression estimator can be approximated as a deterministic quantity. This analysis builds on random matrix tools (3). Note that our derivation of the deterministic equivalent does *not* place the power scaling assumptions on the eigenvalues or alignment coefficients; in fact, it holds for any linear regression setup which satisfies a standard random matrix theory assumption (Assumption 1).

We compute the following deterministic equivalent. As in (3), the asymptotic equivalence notation $u \sim v$ means that u/v tends to 1 as N and P go to ∞.

Lemma 6. *Suppose that $N \geq 1$, $P \geq 1$, $\mathcal{D}_F$, β_1, and β_2 satisfy Assumption 1. Let Σ be the covariance matrix of $\mathcal{D}_F$, and let $\alpha \in [0,1]$ and $\lambda \in (0,1)$ be general parameters. Let $\Sigma_c = (\Sigma + cI)$ for $c \geq 0$, let $B^{sn} = \beta_1 \beta_1^T$, let $B^{df} = (\beta_1 - \beta_2)(\beta_1 - \beta_2)^T$, and let $B^{mx} = (\beta_1 - \beta_2)\beta_1^T$. Let $\kappa = \kappa(\lambda, N, \Sigma)$ defined according to Definition 1 in the supplementary material. Then, it holds that*

$$L_1(\hat{\beta}(\alpha, \lambda, X)) \sim L_1^{det}(\beta_1, \beta_2, \mathcal{D}_F, \lambda, N, \alpha) =: \frac{T_1 + T_2 + T_3 + T_4 + T_5}{Q},$$

where:

$$Q := 1 - \frac{1}{N}\operatorname{Tr}(\Sigma^2 \Sigma_\kappa^{-2}),$$

$$T_1 := \kappa^2 \cdot \operatorname{Tr}(\Sigma \Sigma_\kappa^{-2} B^{sn}), \quad T_2 := (1-\alpha)^2 \left(\operatorname{Tr}\left(\Sigma_\kappa^{-2} \Sigma^3 B^{df}\right)\right)$$

$$T_3 := 2(1-\alpha)\kappa \cdot \operatorname{Tr}\left(\Sigma_\kappa^{-2} \Sigma^2 B^{mx}\right),$$

$$T_4 := -2(1-\alpha)\kappa \frac{1}{N}\operatorname{Tr}(\Sigma^2 \Sigma_\kappa^{-2}) \cdot \operatorname{Tr}\left(\Sigma_\kappa^{-1} \Sigma B^{mx}\right),$$

$$T_5 := (1-\alpha)\frac{1}{N}\operatorname{Tr}(\Sigma^2 \Sigma_\kappa^{-2}) \cdot \left(\operatorname{Tr}\left(\Sigma B^{df}\right) - 2(1-\alpha)\operatorname{Tr}\left(\Sigma_\kappa^{-1} \Sigma^2 B^{df}\right)\right)$$

Lemma 6 shows that the loss can be approximated by a deterministic quantity $L_1^{\mathsf{det}}(\beta_1, \beta_2, \mathcal{D}_F, \lambda, N, \alpha)$ which is sum of five terms, normalized by the standard degrees of freedom correction Q^{-1} (3). The sum $T_1 + T_2 + T_3$ is the loss of infinite-data ridge regression with regularizer κ. Terms T_4 and T_5 capture additional error terms.

In more detail, term T_1/Q captures the standard single-objective environment error for N data points (3): i.e., the population error of the single-objective linear regression problem with regularizer λ where all of the N training data points are labelled with β_i. Term T_2 is similar to the infinite-data ridgeless regression error but is slightly smaller due to regularization. Term T_3 is a cross term that is upper bounded by the geometric mean of term T_1 and term T_2. Term T_4 is another cross term that is subsumed by the other terms. Term T_5 captures an overfitting error that increases with the regularizer κ and decreases with the amount of data N.

From deterministic equivalents to scaling laws. In the following two subsections, using the deterministic equivalent from Lemma 6, we derive *scaling laws*. We make use of the power-law scaling assumptions on the covariance and alignment coefficients, under which the deterministic equivalent takes a cleaner form. We note that strictly speaking, deriving scaling laws requires controlling the error of the deterministic equivalent relative to the actual loss; for simplicity, we do not control errors and instead directly analyze the deterministic equivalent.

Scaling law for the loss. We derive scaling laws for the loss $L_1^{\mathsf{det}} := L_1^{\mathsf{det}}(\beta_1, \beta_2, \mathcal{D}_F, \lambda, N, \alpha)$. We first prove the following scaling law for a general regularizer λ.

Theorem 7. *Suppose that the power-law scaling assumption holds for the eigenvalues and alignment coefficients with scaling exponents $\gamma, \delta > 0$ and correlation coefficient $\rho \in [0, 1)$, suppose that $P = \infty$.* Assume that $\alpha \geq 0.5$ and $\lambda \in (0,1)$. Let $L_1^{det} := L_1^{det}(\beta_1, \beta_2, \mathcal{D}_F, \lambda, N, \alpha)$ be the deterministic equivalent from Lemma 6. Let $\nu := \min(2(1+\gamma), \delta + \gamma)$. Then, the expected loss satisfies $\mathbb{E}_{\mathcal{D}_W}[L_1^{det}] = \Theta(Z)$, where*

$$Z = \underbrace{\max(\lambda^{\frac{\nu}{1+\gamma}}, N^{-\nu})}_{\textit{finite-data error}} + \underbrace{(1-\alpha)^2 \cdot (1-\rho)}_{\textit{mixture error}} + \underbrace{(1-\alpha)\left(\frac{\min(\lambda^{-\frac{1}{1+\gamma}}, N)}{N}\right)(1-\rho)}_{\textit{overfitting error}}.$$

*Strictly speaking, we take a limit as $\Gamma \to \infty$ (where Γ is defined according to Assumption 1).

 Meena Jagadeesan, Michael I. Jordan, Jacob Steinhardt

Theorem 7 illustrates that the loss is the sum of a *finite-data error*, an *overfitting error*, and a *mixture error*. The finite-data error for $L_1^{\mathtt{det}}$ matches the loss in the single-objective environment for N data points labelled with objective β_1. The mixture error equals the loss of the infinite-data ridgeless regression predictor $\beta(\alpha, 0)$. The overfitting error for $L_1^{\mathtt{det}}$ equals the error incurred when the regularizer λ is too small. This term is always at most $(1-\alpha)^{-1}$ times larger than the mixture error, and it is smaller than the mixture error when λ is sufficiently large relative to N.

Due to the overfitting error, the optimal loss is *not* necessarily achieved by taking $\lambda \to 0$ for multi-objective linear regression. In fact, if the regularizer decays too quickly as a function of N (i.e., if $\lambda = o(N^{-1-\gamma})$), then the loss would converge to $\Theta((1-\alpha)(1-\rho))$ in the limit as $N \to \infty$, which is a factor of $(1-\alpha)^{-1}$ higher than the loss $\Theta((1-\alpha)^2(1-\rho))$ of the infinite-data ridgeless predictor $\beta(\alpha, 0)$. The fact that $\lambda \to 0$ is suboptimal reveals a sharp disconnect between the multi-objective setting and the single-objective setting where no explicit regularization is necessary to achieve the optimal loss (see, e.g., 1, 2). Tempered overfitting (4) can similarly occur in single-objective settings with *noisy observations*; in this sense, labelling some of the data with the alternate objective β_2 behaves qualitatively similarly to noisy observations.

In the next result, we compute the optimal regularizer and derive a scaling law under optimal regularization as a corollary of Theorem 7.

Corollary 8 (Formal version of Theorem 2). *Consider the setup of Theorem 7. Then, the loss $\inf_{\lambda \in (0,1)} \mathbb{E}_{\mathcal{D}_W}[L_1^{\mathtt{det}}]$ under optimal regularization can be expressed as:*

$$\begin{cases} \Theta\left(N^{-\nu}\right) & \text{if } N \leq ((1-\alpha)(1-\rho))^{-\frac{1}{\nu}} \\ \Theta\left(\left(\frac{N}{(1-\alpha)(1-\rho)}\right)^{-\frac{\nu}{\nu+1}}\right) & \text{if } N \geq ((1-\alpha)(1-\rho))^{-\frac{1}{\nu}} \\ & \text{and } N \leq (1-\alpha)^{-\frac{2+\nu}{\nu}}(1-\rho)^{-\frac{1}{\nu}} \\ \Theta((1-\alpha)^2(1-\rho)) & \text{if } N \geq (1-\alpha)^{-\frac{2+\nu}{\nu}}(1-\rho)^{-\frac{1}{\nu}}, \end{cases}$$

where $\nu := \min(2(1+\gamma), \delta+\gamma)$.

The scaling law exponent ν^* ranges from ν, to $\nu/(\nu+1)$, to 0 (Figure 2a). To better understand each regime, we provide intuition for when the error term from Theorem 7 dominates, the form of the optimal regularizer, and the behavior of the loss.

- *Regime 1:* $N \leq (1-\alpha)^{-\frac{1}{\nu}}(1-\rho)^{-\frac{1}{\nu}}$. Since N is small, the finite-data error dominates regardless of λ. As a result, as in a single-objective environment, taking $\lambda = O(N^{-1-\gamma})$ recovers the optimal loss up to constants. Note that the loss thus behaves as if all N data points were labelled according to β_i: the learner benefits from *all* of the data, not just the data is labelled according to β_i.

- *Regime 2:* $(1-\alpha)^{-\frac{1}{\nu}}(1-\rho)^{-\frac{1}{\nu}} \leq N \leq (1-\alpha)^{-\frac{2+\nu}{\nu}}(1-\rho)^{-\frac{1}{\nu}}$. In this regime, the finite error term and overfitting error dominate. Taking $\lambda = \Theta\left(\left(\frac{(1-\alpha)(1-\rho)}{N}\right)^{\frac{1+\gamma}{\nu+1}}\right)$, which equalizes the two error terms, recovers the optimal loss up to constants. The loss in this regime improves with N, but at a slower rate than in a single-objective environment.

- *Regime 3:* $N \geq (1-\alpha)^{-\frac{2+\nu}{\nu}}(1-\rho)^{-\frac{1}{\nu}}$. Since N is large, the mixture and the overfitting error terms dominate. Taking $\lambda = \Theta((N(1-\alpha))^{-1-\gamma})$, which equalizes the two error terms, recovers the optimal loss up to constants. The loss behaves (up to the constants) as if there were *infinitely many data points* from the mixture distribution with weight α. This is the minimal possible loss and there is thus no additional benefit for data beyond improving constants.

Scaling law for the excess loss. Now, we turn to scaling laws for the excess loss $\mathbb{E}_{\mathcal{D}_W}[L_1^{\mathtt{det}}(\beta_1, \beta_2, \mathcal{D}_F, \lambda, N, \alpha) - L_1(\beta(\alpha, 0))]$, , which is normalized by the loss of the infinite-data ridgeless predictor $\beta(\alpha, 0)$. We first prove the following scaling law for a general regularizer λ, assuming that $\alpha \geq 0.75$. The assumption that $\alpha \geq 0.75$ simplifies the closed-form expression for the deterministic equivalent of the excess loss; we defer a broader characterization of scaling laws for the excess loss to future work.

Theorem 9. *Suppose that the power-law scaling assumption holds for the eigenvalues and alignment coefficients with scaling exponents $\gamma, \delta > 0$ and correlation coefficient $\rho \in [0, 1)$, suppose that $P = \infty$.*[†] *Assume that $\alpha \geq 0.75$ and $\lambda \in (0, 1)$. Let $L_1^{det} := L_1^{det}(\beta_1, \beta_2, \mathcal{D}_F, \lambda, N, \alpha)$ be the deterministic equivalent from Lemma 6. Let $\nu := \min(2(1+\gamma), \delta+\gamma)$ and let $\nu' = \min(1+\gamma, \delta+\gamma)$ Then, the expected loss $\mathbb{E}_{\mathcal{D}_W}[L_1^{det} - L_1(\beta(\alpha, 0))] = \Theta(Z)$ satisfies:*

$$Z = \underbrace{\max(\lambda^{\frac{\nu}{1+\gamma}}, N^{-\nu})}_{\text{finite-data error}} + \underbrace{(1-\rho)(1-\alpha)\max(\lambda^{\frac{\nu'}{1+\gamma}}, N^{-\nu'})}_{\text{mixture finite-data error}}$$
$$+ \underbrace{(1-\alpha)\left(\frac{\min(\lambda^{-\frac{1}{1+\gamma}}, N)}{N}\right)(1-\rho)}_{\text{overfitting error}}.$$

[†] Strictly speaking, we take a limit as $\Gamma \to \infty$ (where Γ is defined according to Assumption 1).

Theorem 9 illustrates that the loss is the sum of a *finite-data error*, an *overfitting error*, and a *mixture finite-data error*. In comparison with Theorem 7, the difference is that the mixture error is replaced by the mixture finite-data error. Interestingly, the mixture finite-data error exhibits a different asymptotic dependence with respect to λ and N than the finite-data error: the asymptotic rate of decay scales with ν' rather than ν. In fact, the rate is *slower* for the mixture finite-data error than the finite-data error as long as $\delta > 1$ (since this means that $\nu' < \nu$).

Since the optimal excess loss is also not necessarily achieved by taking $\lambda \to 0$, we compute the optimal regularizer for the excess loss and derive a scaling law under optimal regularization as a corollary of Theorem 9.

Corollary 10 (Formal version of Theorem 3)**.** *Consider the setup of Theorem 9. The excess loss* $\inf_{\lambda \in (0,1)}(\mathbb{E}_{\mathcal{D}_W}[L_1^{det} - L_1(\beta(\alpha, 0))])$ *under optimal regularization can be expressed as:*

$$\begin{cases} \Theta\left(N^{-\nu}\right) & \text{if } N \leq (1-\alpha)^{-\frac{1}{\nu}}(1-\rho)^{-\frac{1}{\nu}} \\ \Theta\left(\left(\frac{N}{(1-\alpha)(1-\rho)}\right)^{-\frac{\nu}{\nu+1}}\right) & \text{if } N \geq (1-\alpha)^{-\frac{1}{\nu}}(1-\rho)^{-\frac{1}{\nu}} \\ & \text{and } N \leq ((1-\alpha)(1-\rho))^{-\frac{\nu'+1}{\nu-\nu'}} \\ \Theta\left((1-\alpha)(1-\rho)N^{-\frac{\nu'}{\nu'+1}}\right) & \text{if } N \geq ((1-\alpha)(1-\rho))^{-\frac{\nu'+1}{\nu-\nu'}}, \end{cases}$$

where $\nu := \min(2(1+\gamma), \delta+\gamma)$ *and* $\nu' = \min(1+\gamma, \delta+\gamma)$.

The scaling law exponent ν^* ranges from ν, to $\nu/(\nu+1)$, to $\nu'/(\nu'+1)$ (Figure 2b). The first two regimes behave similarly to Corollary 8, and the key difference arises in the third regime (when N is large). In the third regime ($N \geq (1-\alpha)^{-\frac{\nu'+1}{\nu-\nu'}}(1-\rho)^{-\frac{\nu'+1}{\nu-\nu'}}$), the mixture finite-data error and the overfitting error terms dominate. Taking $\lambda = \Theta\left(N^{-\frac{1+\gamma}{\nu'+1}}\right)$—which equalizes these two error terms—recovers the optimal loss up to constants. The resulting scaling behavior captures the fact that in this regime, additional data meaningfully improves the *excess loss*, even though additional data only improves the loss in terms of constants.

2. Proofs for Warm Up: Infinite-Data Incumbent and Unconstrained Entrant

In this section, we prove Theorem 1. First, we state relevant facts (Appendix 2.A) and prove intermediate lemmas (Appendix 2.B), and then we use these ingredients to prove Theorem 1(Appendix 2.C). Throughout this section, we let

$$L^*(\rho) = \mathbb{E}_{\mathcal{D}_W}[(\beta_1 - \beta_2)\Sigma(\beta_1 - \beta_2)^T].$$

Moreover, let

$$\beta(\alpha, \lambda) = \text{argmin}_\beta \left(\alpha \cdot \mathbb{E}_{X \sim \mathcal{D}_F}[(\langle \beta - \beta_1, X\rangle)^2] + (1-\alpha) \cdot \mathbb{E}_{X \sim \mathcal{D}_F}[(\langle \beta - \beta_2, X\rangle)^2] + \lambda \|\beta\|_2^2\right)$$

be the infinite-data ridge regression predictor.

2.A. Facts. We can explicitly solve for the infinite-data ridge regression predictor

$$\begin{aligned}\beta(\alpha, \lambda) &= \text{argmin}_\beta \left(\alpha \cdot \mathbb{E}_{x \sim \mathcal{D}_F}[\langle \beta - \beta_1, x\rangle^2] + (1-\alpha) \cdot \mathbb{E}_{x \sim \mathcal{D}_F}[\langle \beta - \beta_2, x\rangle^2] + \lambda ||\beta||_2^2\right) \\ &= \Sigma(\Sigma + \lambda I)^{-1}(\alpha\beta_1 + (1-\alpha)\beta_2).\end{aligned}$$

A simple calculation shows that $\mathbb{E}_{\mathcal{D}_W}[L_1(\beta(\alpha, 0))] = (1-\alpha)^2 L^*(\rho)$ and $\mathbb{E}_{\mathcal{D}_W}[L_2(\beta(\alpha, 0))] = \alpha^2 L^*(\rho)$. Thus, it holds that:

$$\alpha \mathbb{E}_{\mathcal{D}_W}[L_1(\beta(\alpha, 0))] + (1-\alpha)\mathbb{E}_{\mathcal{D}_W}[L_2(\beta(\alpha, 0))] = \alpha(1-\alpha)L^*(\rho).$$

Moreover, by the definition of the ridge regression objective, we see that:

$$\alpha \mathbb{E}_{\mathcal{D}_W}[L_1(\beta(\alpha, \lambda))] + (1-\alpha)\mathbb{E}_{\mathcal{D}_W}[L_2(\beta(\alpha, \lambda))] \geq \alpha \mathbb{E}_{\mathcal{D}_W}[L_1(\beta(\alpha, 0))] + (1-\alpha)\mathbb{E}_{\mathcal{D}_W}[L_2(\beta(\alpha, 0))].$$

2.B. Lemmas. The first lemma upper bounds the performance loss when there is regularization.

Lemma 11. *Suppose that the power-law scaling assumptions hold with exponents $\gamma, \delta > 0$ and correlation coefficient $\rho \in [0, 1)$, and suppose that $P = \infty$. Let $L^*(\rho) = (\beta_1 - \beta_2)^T \Sigma(\beta_1 - \beta_2)^T$. Let*

$$\beta(\alpha, \lambda) = \text{argmin}_\beta \left(\alpha \cdot \mathbb{E}_{X \sim \mathcal{D}_F}[(\langle \beta - \beta_1, X\rangle)^2] + (1-\alpha) \cdot \mathbb{E}_{X \sim \mathcal{D}_F}[(\langle \beta - \beta_2, X\rangle)^2] + \lambda \|\beta\|_2^2\right)$$

be the infinite-data ridge regression predictor. Assume that $\alpha \geq 1/2$. Then it holds that

$$\mathbb{E}_{\mathcal{D}_W}[L_1(\beta(\alpha, \lambda))] \geq (1-\alpha)^2 L^*(\rho)$$

and

$$\frac{\mathbb{E}_{\mathcal{D}_W}[L_1(\beta(\alpha, \lambda))]}{\mathbb{E}_{\mathcal{D}_W}[L_2(\beta(\alpha, \lambda))]} \geq \frac{(1-\alpha)^2}{\alpha^2}.$$

Proof. We define the quantities:

$$A := \lambda^2 \sum_{i=1}^{P} \frac{\lambda_i}{(\lambda_i + \lambda)^2} i^{-\delta}$$

$$B := (1-\alpha)^2 (1-\rho)^2 \sum_{i=1}^{P} \frac{\lambda_i^3}{(\lambda_i + \lambda)^2} i^{-\delta}$$

$$C := \lambda (1-\rho) \sum_{i=1}^{P} \frac{\lambda_i^2}{(\lambda_i + \lambda)^2} i^{-\delta}.$$

We compute the performance loss as follows:

$$\begin{aligned}
&\mathbb{E}_{\mathcal{D}_W}[L_1(\beta(\alpha, \lambda))] \\
&= \mathbb{E}_{\mathcal{D}_W}[\mathrm{Tr}(\Sigma(\beta_1 - \beta(\alpha,\lambda))(\beta_1 - \beta(\alpha,\lambda))^T)] \\
&= \mathbb{E}_{\mathcal{D}_W}\left[\mathrm{Tr}\left((\Sigma + \lambda I)^{-2} \Sigma \left(\lambda \beta_1 + \Sigma \cdot (1-\alpha)(\beta_1 - \beta_2)\right)\left(\lambda \beta_1 + \Sigma \cdot (1-\alpha)(\beta_1 - \beta_2)\right)^T\right)\right] \\
&= \mathbb{E}_{\mathcal{D}_W}\left[\mathrm{Tr}\left((\Sigma + \lambda I)^{-2} \Sigma \cdot \left(\lambda \beta_1 + \Sigma \cdot (1-\alpha)(\beta_1 - \beta_2)\right)\left(\lambda \beta_1 + \Sigma \cdot (1-\alpha)(\beta_1 - \beta_2)\right)^T\right)\right] \\
&= \lambda^2 \mathbb{E}_{\mathcal{D}_W}\left[\mathrm{Tr}\left((\Sigma + \lambda I)^{-2} \Sigma \cdot \beta_1 \beta_1^T\right)\right] + (1-\alpha)^2 \mathbb{E}_{\mathcal{D}_W}\left[\mathrm{Tr}\left((\Sigma + \lambda I)^{-2} \Sigma^3 \cdot (\beta_1 - \beta_2)(\beta_1 - \beta_2)^T\right)\right] \\
&\quad + \lambda(1-\alpha) \mathbb{E}_{\mathcal{D}_W}\left[\mathrm{Tr}\left((\Sigma + \lambda I)^{-2} \Sigma^2 \cdot \beta_1 (\beta_1 - \beta_2)^T\right)\right] \\
&= \lambda^2 \sum_{i=1}^{P} \frac{\lambda_i}{(\lambda_i + \lambda)^2} \mathbb{E}_{\mathcal{D}_W}[\langle \beta_1, v_i \rangle^2] + (1-\alpha)^2 \sum_{i=1}^{P} \frac{\lambda_i^3}{(\lambda_i + \lambda)^2} \mathbb{E}_{\mathcal{D}_W}[\langle \beta_1 - \beta_2, v_i \rangle^2] \\
&\quad + \lambda(1-\alpha) \sum_{i=1}^{P} \frac{\lambda_i^2}{(\lambda_i + \lambda)^2} \mathbb{E}_{\mathcal{D}_W}\left[\langle \beta_1, v_i \rangle \langle \beta_1 - \beta_2, v_i \rangle\right] \\
&= \lambda^2 \sum_{i=1}^{P} \frac{\lambda_i}{(\lambda_i + \lambda)^2} i^{-\delta} + (1-\alpha)^2 (1-\rho)^2 \sum_{i=1}^{P} \frac{\lambda_i^3}{(\lambda_i + \lambda)^2} i^{-\delta} + \lambda(1-\alpha)(1-\rho) \sum_{i=1}^{P} \frac{\lambda_i^2}{(\lambda_i + \lambda)^2} i^{-\delta} \\
&= A + (1-\alpha)^2 B + (1-\alpha) C.
\end{aligned}$$

An analogous calculation shows that the safety violation can be written as:

$$\mathbb{E}_{\mathcal{D}_W}[L_2(\beta(\alpha, \lambda))] = A + \alpha^2 B + \alpha C$$

Since $\alpha \geq 1/2$, then it holds that:

$$\frac{\mathbb{E}_{\mathcal{D}_W}[L_1(\beta(\alpha, \lambda))]}{\mathbb{E}_{\mathcal{D}_W}[L_2(\beta(\alpha, \lambda))]} = \frac{A + (1-\alpha)^2 B + (1-\alpha) C}{A + \alpha B + \alpha C} \geq \frac{(1-\alpha)^2}{\alpha^2}.$$

Combining this with the facts from Appendix 2.A—which imply that $\alpha \mathbb{E}_{\mathcal{D}_W}[L_1(\beta(\alpha, \lambda))] + (1-\alpha)\mathbb{E}_{\mathcal{D}_W}[L_2(\beta(\alpha, \lambda))] \geq \alpha(1-\alpha) L^*(\rho)$—we have that $\mathbb{E}_{\mathcal{D}_W}[L_1(\beta(\alpha, \lambda))] \geq (1-\alpha)^2 L^*(\rho)$ as desired. □

The following lemma computes the optimal values of α and λ for the incumbent.

Lemma 12. *Suppose that the power-law scaling assumptions hold with exponents $\gamma, \delta > 0$ and correlation coefficient $\rho \in [0, 1)$, and suppose that $P = \infty$. Let $L^*(\rho) = \mathbb{E}_{\mathcal{D}_W}[(\beta_1 - \beta_2)^T \Sigma (\beta_1 - \beta_2)^T]$. Suppose that $N_I = \infty$, and suppose that the safety constraint τ_I satisfies (1). Then it holds that $\alpha_I = \sqrt{\frac{\min(\tau_I, L^*(\rho))}{L^*(\rho)}}$, and $\lambda_I = 0$ is optimal for the incumbent. Moreover, it holds that:*

$$\mathbb{E}_{\mathcal{D}_W}[L_1^*(\beta_1, \beta_2, \mathcal{D}_F, \lambda_I, \infty, \alpha_O)] = (\sqrt{L^*(\rho)} - \sqrt{\min(\tau_I, L^*(\rho))})^2.$$

Proof. First, we apply Lemma 26 with $N = \infty$ to see that:

$$\mathbb{E}_{\mathcal{D}_W}[L_1^*(\beta_1, \beta_2, \mathcal{D}_F, \lambda, \infty, \alpha)] = \mathbb{E}_{\mathcal{D}_W}[L_1(\beta(\alpha, \lambda))]$$

and apply the definition of L_2^* to see that:

$$\mathbb{E}_{\mathcal{D}_W}[L_2^*(\beta_1, \beta_2, \mathcal{D}_F, \alpha)] = \mathbb{E}_{\mathcal{D}_W}[L_2(\beta(\alpha, 0))].$$

Let $\alpha^* = \sqrt{\frac{\min(\tau_I, L^*(\rho))}{L^*(\rho)}}$. By the assumption in the lemma statement, we know that:

$$\alpha^* \geq \sqrt{\frac{\mathbb{E}_{\mathcal{D}_W}[L_2^*(\beta_1, \beta_2, \mathcal{D}_F, 0.5)]}{L^*(\rho)}} = 0.5.$$

We show that $(\alpha_I, \lambda_I) = (\alpha^*, 0)$. Assume for sake of contradiction that $(\alpha, \lambda) \neq (\alpha^*, 0)$ satisfies the safety constraint $\mathbb{E}_{\mathcal{D}_W}[L_2^*(\beta_1, \beta_2, \mathcal{D}_F, \alpha)] \leq \tau_I$ and achieves strictly better performance loss:

$$\mathbb{E}_{\mathcal{D}_W}[L_1^*(\beta_1, \beta_2, \mathcal{D}_F, \lambda, \infty, \alpha)] < \mathbb{E}_{\mathcal{D}_W}[L_1^*(\beta_1, \beta_2, \mathcal{D}_F, 0, \infty, \alpha^*)].$$

We split into two cases: $\alpha^* = \alpha, \lambda \neq 0$ and $\alpha^* \neq \alpha$.

Case 1: $\alpha^* = \alpha, \lambda \neq 0$. By Lemma 11, we know that

$$\mathbb{E}[L_1^*(\beta_1, \beta_2, \mathcal{D}_F, \lambda, \infty, \alpha^*)] = \mathbb{E}_{\mathcal{D}_W}[L_1(\beta(\alpha^*, \lambda))] \geq (1 - \alpha^*)^2 L^*(\rho).$$

Equality is obtained at $\lambda = 0$, which is a contradiction.

Case 2: $\alpha \neq \alpha^*$. By Lemma 11, it must hold that $\alpha > \alpha^*$ in order for the performance to beat that of $(\alpha^*, 0)$. However, this means that the safety constraint

$$\mathbb{E}_{\mathcal{D}_W}[L_2^*(\beta_1, \beta_2, \mathcal{D}_F, \alpha)] = \alpha^2 L^*(\rho) > (\alpha^*)^2 L^*(\rho) = \tau_I$$

is violated, which is a contradiction.

Concluding the statement. This means that$(\alpha_I, \lambda_I) = (\alpha^*, 0)$, which also means that:

$$\begin{aligned}
\mathbb{E}_{\mathcal{D}_W}[L_1^*(\beta_1, \beta_2, \mathcal{D}_F, \lambda_I, \infty, \alpha_I)] &= \mathbb{E}_{\mathcal{D}_W}[L_1(\beta(\alpha_I, \lambda_I))] \\
&= (1 - \alpha_I)^2 \mathbb{E}_{\mathcal{D}_W}[(\beta_1 - \beta_2)^T \Sigma (\beta_1 - \beta_2)] \\
&= \left(\sqrt{L^*(\rho)} - \sqrt{\min(\tau_I, L^*(\rho))}\right)^2.
\end{aligned}$$

□

The following claim calculates $\mathbb{E}_{\mathcal{D}_W}[(\beta_1 - \beta_2)^T \Sigma (\beta_1 - \beta_2)]$.

Claim 13. *Suppose that the power-law scaling assumptions hold with exponents $\gamma, \delta > 0$ and correlation coefficient $\rho \in [0, 1)$, suppose that $P = \infty$. Then it holds that:*

$$\mathbb{E}_{\mathcal{D}_W}[(\beta_1 - \beta_2)^T \Sigma (\beta_1 - \beta_2)] = 2(1 - \rho) \left(\sum_{i=1}^{P} i^{-\delta-1-\gamma}\right) = \Theta(1 - \rho).$$

Proof. Let $\Sigma = V \Lambda V^T$ be the eigendecomposition of Σ, where Λ is a diagonal matrix consisting of the eigenvalues. We observe that

$$\mathbb{E}_{\mathcal{D}_W}[\langle \beta_1 - \beta_2, v_i \rangle^2] = \mathbb{E}_{\mathcal{D}_W}[\langle \beta_1, v_i \rangle^2] + \mathbb{E}_{\mathcal{D}_W}[\langle \beta_2, v_i \rangle^2] - 2\mathbb{E}_{\mathcal{D}_W}[\langle \beta_1, v_i \rangle \langle \beta_2, v_i \rangle] = i^{-\delta} + i^{-\delta} - 2\rho i^{-\delta} = 2(1 - \rho) i^{-\delta}.$$

This means that:

$$\begin{aligned}
\mathbb{E}_{\mathcal{D}_W}[(\beta_1 - \beta_2)^T \Sigma (\beta_1 - \beta_2)] &= \mathrm{Tr}(\Sigma \mathbb{E}_{\mathcal{D}_W}[(\beta_1 - \beta_2)(\beta_1 - \beta_2)^T]) \\
&= \mathrm{Tr}(\Lambda \mathbb{E}_{\mathcal{D}_W}[V^T (\beta_1 - \beta_2)(\beta_1 - \beta_2)^T V]) \\
&= \sum_{i=1}^{P} i^{-1-\gamma} \mathbb{E}_{\mathcal{D}_W}[\langle \beta_1 - \beta_2, v_i \rangle^2] \\
&= 2(1 - \rho) \sum_{i=1}^{P} i^{-\delta-1-\gamma} \\
&= \Theta(1 - \rho).
\end{aligned}$$

□

2.C. Proof of Theorem 1. We prove Theorem 1using the above lemmas along with Corollary 8(the proof of which we defer to Appendix 4).

Proof of Theorem 1. We analyze (α_C, λ_C) first for the incumbent $C = I$ and then for the entrant $C = E$.

Analysis of the incumbent $C = I$. To compute α_I and λ_I, we apply Lemma 12. By Lemma 12, we see that:

$$\mathbb{E}_{\mathcal{D}_W}[L_1^*(\beta_1, \beta_2, \mathcal{D}_F, \lambda_I, \infty, \alpha_I)] = \left(\sqrt{L^*(\rho)} - \sqrt{\min(\tau_I, L^*(\rho))}\right)^2.$$

Analysis of the entrant $C = E$. Since the entrant faces no safety constraint, the entrant can choose any $\alpha \in [0.5, 1]$. We apply Corollary 8to see that:

$$\mathbb{E}_{\mathcal{D}_W}[L_1^*(\beta_1, \beta_2, \mathcal{D}_F, \lambda_E, N, \alpha_E)] = \inf_{\alpha \in [0.5,1]} \inf_{\lambda > 0} \mathbb{E}_{\mathcal{D}_W}[L_1^*(\beta_1, \beta_2, \mathcal{D}_F, \lambda, N, \alpha)] = \Theta\left(N^{-\nu}\right),$$

which means that:

$$N_E^*(\infty, \tau_I, \infty, \mathcal{D}_W, \mathcal{D}_F) = \Theta\left(\left(\sqrt{L^*(\rho)} - \sqrt{\min(\tau_I, L^*(\rho)}\right)^{-2/\nu}\right)$$

as desired. We can further apply Claim 13 to see that $L^*(\rho) = \Theta(1-\rho)$. □

3. Proofs for Generalized Analysis of the Market-entry Threshold

3.A. Proofs for Finite data for the incumbent. We prove Theorem 4. The main technical tool is Theorem 7, the proof of which we defer to Appendix 4.

Proof of Theorem 4. We analyze (α_C, λ_C) first for the incumbent $C = I$ and then for the entrant $C = E$. Like in the theorem statement, let $L^*(\rho) = \mathbb{E}_{\mathcal{D}_W}[(\beta_1 - \beta_2)^T \Sigma (\beta_1 - \beta_2)] = \Theta(1-\rho)$ (Claim 13) and $G_I := (\sqrt{L^*(\rho)} - \sqrt{\min(\tau_I, L^*(\rho))})^2$, and $\nu = \min(2(1+\gamma), \delta + \gamma)$.

Analysis of the incumbent $C = I$. Recall from the facts in Appendix 2.A that:

$$L_1^*(\beta_1, \beta_2, \mathcal{D}_F, \alpha) = \alpha^2 L^*(\rho).$$

This means that the safety constraint is satisfied if and only if $\alpha_I \leq \sqrt{\frac{\min(\tau_I, L^*(\rho))}{L^*(\rho)}} =: \alpha^*$. The bound in Corollary 8implies that:

$$\begin{aligned}
&\mathbb{E}_{\mathcal{D}_W}[L_1^*(\beta_1, \beta_2, \mathcal{D}_F, \lambda_I, N_I, \alpha_I)] \\
&= \inf_{\alpha \in [0.5, \alpha^*]} \inf_{\lambda > 0} \mathbb{E}_{\mathcal{D}_W}[L_1^*(\beta_1, \beta_2, \mathcal{D}_F, \lambda, N_I, \alpha)] \\
&= \Theta\left(\inf_{\lambda > 0} \mathbb{E}_{\mathcal{D}_W}\left[L_1^*\left(\beta_1, \beta_2, \Sigma, \lambda, N_I, \alpha^*\right)\right]\right) \\
&= \begin{cases} \Theta\left(N_I^{-\nu}\right) & \text{if } N_I \leq (1-\alpha^*)^{-\frac{1}{\nu}}(1-\rho)^{-\frac{1}{\nu}} \\ \Theta\left(\left(\frac{N_I}{(1-\alpha^*)(1-\rho)}\right)^{-\frac{\nu}{\nu+1}}\right) & \text{if } (1-\alpha^*)^{-\frac{1}{\nu}}(1-\rho)^{-\frac{1}{\nu}} \leq N_I \leq (1-\alpha^*)^{-\frac{2+\nu}{\nu}}(1-\rho)^{-\frac{1}{\nu}} \\ \Theta((1-\alpha^*)^2(1-\rho)) & \text{if } N_I \geq (1-\alpha^*)^{-\frac{2+\nu}{\nu}}(1-\rho)^{-\frac{1}{\nu}}, \end{cases} \\
&= \begin{cases} \Theta\left(N_I^{-\nu}\right) & \text{if } N_I \leq G_I^{-\frac{1}{2\nu}}(1-\rho)^{-\frac{1}{2\nu}} \\ \Theta\left(N_I^{-\frac{\nu}{\nu+1}} \cdot G_I^{\frac{\nu}{2(\nu+1)}}(1-\rho)^{\frac{\nu}{2(\nu+1)}}\right) & \text{if } G_I^{-\frac{1}{2\nu}}(1-\rho)^{-\frac{1}{2\nu}} \leq N_I \leq G_I^{-\frac{1}{2}-\frac{1}{\nu}}(1-\rho)^{\frac{1}{2}} \\ \Theta\left(G_I\right) & \text{if } N_I \geq G_I^{-\frac{1}{2}-\frac{1}{\nu}}(1-\rho)^{\frac{1}{2}} \end{cases}.
\end{aligned}$$

Analysis of the entrant $C = E$. Since the entrant faces no safety constraint, the entrant can choose any $\alpha \in [0.5, 1]$. We apply Corollary 7to see that:

$$\mathbb{E}_{\mathcal{D}_W}[L_1^*(\beta_1, \beta_2, \mathcal{D}_F, \lambda_E, N, \alpha_E)] = \inf_{\alpha \in [0.5,1]} \inf_{\lambda > 0} \mathbb{E}_{\mathcal{D}_W}[L_1^*(\beta_1, \beta_2, \mathcal{D}_F, \lambda, N, \alpha)] = \Theta\left(N^{-\nu}\right),$$

which means that:

$$N_E^*(N_I, \tau_I, \infty, \mathcal{D}_W, \mathcal{D}_F) = \begin{cases} \Theta\left(N_I\right) & \text{if } N_I \leq G_I^{-\frac{1}{2\nu}}(1-\rho)^{-\frac{1}{2\nu}} \\ \Theta\left(N_I^{\frac{1}{\nu+1}} \cdot G_I^{-\frac{1}{2(\nu+1)}}(1-\rho)^{-\frac{1}{2(\nu+1)}}\right) & \text{if } G_I^{-\frac{1}{2\nu}}(1-\rho)^{-\frac{1}{2\nu}} \leq N_I \leq G_I^{-\frac{1}{2}-\frac{1}{\nu}}(1-\rho)^{\frac{1}{2}} \\ \Theta\left(G_I^{-\frac{1}{\nu}}\right) & \text{if } N_I \geq G_I^{-\frac{1}{2}-\frac{1}{\nu}}(1-\rho)^{\frac{1}{2}}. \end{cases}$$

as desired.

□

3.B. Proofs for Safety constraint for the new company. We prove Theorem 5. When the the safety constraints of the two firms are sufficiently close, it no longer suffices to analyze the loss up to constants for the entrant, and we require a more fine-grained analysis of the error terms than is provided in the scaling laws in Corollary 8. In this case, we turn to scaling laws for the *excess loss* as given by Corollary 10.

Proof of Theorem 5. We analyze (α_C, λ_C) first for the incumbent $C = I$ and then for the entrant $C = E$. Like in the theorem statement, let $L^*(\rho) = \mathbb{E}_{\mathcal{D}_W}[(\beta_1 - \beta_2)^T \Sigma (\beta_1 - \beta_2)] = \Theta(1-\rho)$ (Claim 13), $G_I = (\sqrt{L^*(\rho)} - \sqrt{\min(\tau_I, L^*(\rho))})^2$, $G_E = (\sqrt{L^*(\rho)} - \sqrt{\min(\tau_E, L^*(\rho))})^2$, $D = G_I - G_E$, and $\nu = \min(2(1+\gamma), \delta + \gamma)$.

Analysis of the incumbent $C = I$. Since the incumbent has infinite data, we apply Lemma 12 to see that:

$$\begin{aligned}\mathbb{E}_{\mathcal{D}_W}[L_1^*(\beta_1, \beta_2, \mathcal{D}_F, \lambda_I, \infty, \alpha_I)] &= \left(\sqrt{L^*(\rho)} - \sqrt{\min(\tau_I, L^*(\rho))}\right)^2 \\ &= D + G_E.\end{aligned}$$

Analysis of the entrant $C = E$. Recall from the facts in Appendix 2.A that:

$$L_1^*(\beta_1, \beta_2, \mathcal{D}_F, \alpha) = \alpha^2 L^*(\rho).$$

This means that the safety constraint is satisfied if and only if $\alpha_E \le \sqrt{\frac{\min(\tau_E, L^*(\rho))}{L^*(\rho)}} =: \alpha^*$. The bound in Corollary 10implies that:

$$\begin{aligned}
&\mathbb{E}_{\mathcal{D}_W}[L_1^*(\beta_1, \beta_2, \mathcal{D}_F, \lambda_E, N, \alpha_E)] \\
&= \inf_{\alpha \in [0.5, \alpha^*]} \inf_{\lambda > 0} \mathbb{E}_{\mathcal{D}_W}[L_1^*(\beta_1, \beta_2, \mathcal{D}_F, \lambda, N, \alpha)] \\
&= \inf_{\alpha \in [0.5, \alpha^*]} \left(\inf_{\lambda > 0} \left(\mathbb{E}_{\mathcal{D}_W}[L_1^*(\beta_1, \beta_2, \mathcal{D}_F, \lambda, N, \alpha) - L_1(\beta(\alpha, 0))] \right) + \mathbb{E}_{\mathcal{D}_W}[L_1(\beta(\alpha, 0))] \right) \\
&= \inf_{\alpha \in [0.5, \alpha^*]} \left(\inf_{\lambda > 0} \left(\mathbb{E}_{\mathcal{D}_W}[L_1^*(\beta_1, \beta_2, \mathcal{D}_F, \lambda, N, \alpha) - L_1(\beta(\alpha, 0))] \right) + (1-\alpha)^2 L^*(\rho) \right) \\
&= \Theta\left(\inf_{\lambda > 0} \left(\mathbb{E}_{\mathcal{D}_W}[L_1^*(\beta_1, \beta_2, \mathcal{D}_F, \lambda, N, \alpha) - L_1(\beta(\alpha^*, 0))] \right) \right) + (1-\alpha^*)^2 L^*(\rho) \\
&= \begin{cases} (1-\alpha^*)^2 L^*(\rho) + \Theta\left(N^{-\nu}\right) & \text{if } N \le (1-\alpha^*)^{-\frac{1}{\nu}} (1-\rho)^{-\frac{1}{\nu}} \\ (1-\alpha^*)^2 L^*(\rho) + \Theta\left(\left(\frac{N}{(1-\alpha^*)(1-\rho)} \right)^{-\frac{\nu}{\nu+1}} \right) & \text{if } (1-\alpha^*)^{-\frac{1}{\nu}} (1-\rho)^{-\frac{1}{\nu}} \le N \le (1-\alpha^*)^{-\frac{\nu'+1}{\nu-\nu'}} (1-\rho)^{-\frac{\nu'+1}{\nu-\nu'}} \\ (1-\alpha^*)^2 L^*(\rho) + \Theta\left((1-\alpha^*)(1-\rho) N^{-\frac{\nu'}{\nu'+1}} \right) & \text{if } N \ge (1-\alpha^*)^{-\frac{\nu'+1}{\nu-\nu'}} (1-\rho)^{-\frac{\nu'+1}{\nu-\nu'}}, \end{cases} \\
&= \begin{cases} G_E + \Theta\left(N^{-\nu}\right) & \text{if } N \le (1-\alpha^*)^{-\frac{1}{\nu}} (1-\rho)^{-\frac{1}{\nu}} \\ G_E + \Theta\left(\left(\frac{N}{(1-\alpha^*)(1-\rho)} \right)^{-\frac{\nu}{\nu+1}} \right) & \text{if } (1-\alpha^*)^{-\frac{1}{\nu}} (1-\rho)^{-\frac{1}{\nu}} \le N \le (1-\alpha^*)^{-\frac{\nu'+1}{\nu-\nu'}} (1-\rho)^{-\frac{\nu'+1}{\nu-\nu'}} \\ G_E + \Theta\left((1-\alpha^*)(1-\rho) N^{-\frac{\nu'}{\nu'+1}} \right) & \text{if } N \ge (1-\alpha^*)^{-\frac{\nu'+1}{\nu-\nu'}} (1-\rho)^{-\frac{\nu'+1}{\nu-\nu'}}, \end{cases}.
\end{aligned}$$

Using this, we can compute the market-entry threshold as follows:

$$\begin{aligned}
&N_E^*(\infty, \tau_I, \tau_E, \mathcal{D}_W, \mathcal{D}_F) \\
&= \begin{cases} \Theta(D^{-\frac{1}{\nu}}) & \text{if } D \ge (1-\alpha^*)(1-\rho) \\ \Theta\left(D^{-\frac{\nu+1}{\nu}} (1-\alpha^*)(1-\rho) \right) & \text{if } (1-\alpha^*)^{\frac{\nu}{\nu-\nu'}} (1-\rho)^{\frac{\nu}{\nu-\nu'}} \le D \le (1-\alpha^*)(1-\rho) \\ \Theta\left(\left(\frac{D}{(1-\alpha^*)(1-\rho)} \right)^{-\frac{\nu'+1}{\nu'}} \right) & \text{if } D \le (1-\alpha^*)^{\frac{\nu}{\nu-\nu'}} (1-\rho)^{\frac{\nu}{\nu-\nu'}} \end{cases} \\
&= \begin{cases} \Theta(D^{-\frac{1}{\nu}}) & \text{if } D \ge G_E^{\frac{1}{2}} (1-\rho)^{\frac{1}{2}} \\ \Theta\left(D^{-\frac{\nu+1}{\nu}} G_E^{\frac{1}{2}} (1-\rho)^{\frac{1}{2}} \right) & \text{if } G_E^{\frac{\nu}{2(\nu-\nu')}} (1-\rho)^{\frac{\nu}{2(\nu-\nu')}} \le D \le G_E^{\frac{1}{2}} (1-\rho)^{\frac{1}{2}} \\ \Theta\left(\left(\frac{D}{G_E^{\frac{1}{2}} (1-\rho)^{\frac{1}{2}}} \right)^{-\frac{\nu'+1}{\nu'}} \right) & \text{if } D \le G_E^{\frac{\nu}{2(\nu-\nu')}} (1-\rho)^{\frac{\nu}{2(\nu-\nu')}} \end{cases}
\end{aligned}$$

□

4. Proofs for Deriving Scaling Laws for Multi-Objective Environments

In this section, we derive a deterministic equivalent and scaling laws for high-dimensional multi-objective linear regression. Before diving into this, we introduce notation, derive a basic decomposition, and give an outline for the remainder of the section.

Notation. Recall that (X_i, Y_i) denotes the labelled training dataset. Let the sample covariance be:

$$\hat{\Sigma} = \frac{1}{N}\sum_{i=1}^{N} X_i X_i^T.$$

We also consider the following reparameterization where we group together inputs according to how they are labelled. For $j \in \{1, 2\}$, we let $X_{1,j}, \ldots, X_{N_j,j}$ be the inputs labelled by β_j. We let

$$\hat{\Sigma}_1 = \frac{1}{N_1}\sum_{i=1}^{N_1} X_{i,1} X_{i,1}^T$$
$$\hat{\Sigma}_2 = \frac{1}{N_2}\sum_{i=1}^{N_2} X_{i,2} X_{i,2}^T.$$

It is easy to see that $\hat{\Sigma} = \alpha\hat{\Sigma}_1 + (1-\alpha)\hat{\Sigma}_2$. Moreover, $\mathbb{E}[\hat{\Sigma}] = \mathbb{E}[\hat{\Sigma}_1] = \mathbb{E}[\hat{\Sigma}_2] = \Sigma$. Furthermore, $\hat{\Sigma}_1$ and $\hat{\Sigma}_2$ are fully independent. We let $\sim$ denote asymptotic equivalence following (3).

Basic decomposition. A simple calculation shows that the solution and population-level loss of ridge regression takes the following form.

Claim 14. *Assume the notation above. Let $B^{sn} = \beta_1\beta_1^T$, let $B^{df} = (\beta_1 - \beta_2)(\beta_1 - \beta_2)^T$, and let $B^{mx} = (\beta_1 - \beta_2)\beta_1^T$. The learned predictor takes the form:*

$$\hat{\beta}(\alpha, \lambda, X) = (\hat{\Sigma} + \lambda I)^{-1}(\alpha\hat{\Sigma}_1\beta_1 + (1-\alpha)\hat{\Sigma}_2\beta_2).$$

Moreover, it holds that:

$$L_1(\hat{\beta}(\alpha, \lambda, X)) = \underbrace{\lambda^2 \operatorname{Tr}((\hat{\Sigma} + \lambda I)^{-1}\Sigma(\hat{\Sigma} + \lambda I)^{-1}B^{sn})}_{(T1)} + \underbrace{(1-\alpha)^2 \operatorname{Tr}(\hat{\Sigma}_2(\hat{\Sigma} + \lambda I)^{-1}\Sigma(\hat{\Sigma} + \lambda I)^{-1}\hat{\Sigma}_2 B^{df})}_{(T2)}$$
$$+ \underbrace{2\lambda(1-\alpha) \cdot \operatorname{Tr}((\hat{\Sigma} + \lambda I)^{-1}\Sigma(\hat{\Sigma} + \lambda I)^{-1}\hat{\Sigma}_2 B^{mx})}_{(T3)}.$$

Proof. For $1 \leq i \leq N$, let Y_i be the label for input X_i in the training dataset. For $i \in \{1, 2\}$ and $1 \leq i \leq N_i$, let $Y_{i,j} := \langle \beta_i, X_{i,j}\rangle$ be the label for the input $X_{i,j}$ according to β_i.

For the first part, it follows from standard analyses of ridge regression that the learned predictor takes the form:

$$\begin{aligned}
\hat{\beta}(\alpha, \lambda, X) &= (\hat{\Sigma} + \lambda I)^{-1}\left(\frac{1}{N}\sum_{i=1}^{N} X_i Y_i\right) \\
&= (\hat{\Sigma} + \lambda I)^{-1}\left(\frac{1}{N}\sum_{i=1}^{N} X_{i,1} Y_{i,1} + \frac{1}{N}\sum_{i=1}^{N} X_{i,2} Y_{i,2}\right) \\
&= (\hat{\Sigma} + \lambda I)^{-1}\left(\alpha\hat{\Sigma}_1\beta_1 + (1-\alpha)\hat{\Sigma}_2\beta_2\right)
\end{aligned}$$

as desired.

For the second part, we first observe that the difference $\beta_1 - \hat{\beta}(\alpha, \lambda, X)$ takes the form:

$$\begin{aligned}
\beta_1 - \hat{\beta}(\alpha, \lambda, X) &= \beta_1 - (\hat{\Sigma} + \lambda I)^{-1}\left(\alpha\hat{\Sigma}_1\beta_1 + (1-\alpha)\hat{\Sigma}_2\beta_2\right) \\
&= (\hat{\Sigma} + \lambda I)^{-1}\left(\lambda\beta_1 + (1-\alpha)\hat{\Sigma}_2(\beta_1 - \beta_2)\right).
\end{aligned}$$

This means that:

$$\begin{aligned}
&L_1(\hat{\beta}(\alpha, \lambda, X)) \\
&= (\beta_1 - \hat{\beta}(\alpha, \lambda, X))^T\Sigma(\beta_1 - \hat{\beta}(\alpha, \lambda, X)) \\
&= \left(\lambda\beta_1 + (1-\alpha)\hat{\Sigma}_2(\beta_1 - \beta_2)\right)^T(\hat{\Sigma} + \lambda I)^{-1}\Sigma(\hat{\Sigma} + \lambda I)^{-1}\left(\lambda\beta_1 + (1-\alpha)\hat{\Sigma}_2(\beta_1 - \beta_2)\right) \\
&= \lambda^2 \cdot \beta_1^T\hat{\Sigma} + \lambda I)^{-1}\Sigma(\hat{\Sigma} + \lambda I)^{-1}\beta_1 + (1-\alpha)^2 \cdot (\beta_1 - \beta_2)^T\hat{\Sigma}_2\hat{\Sigma} + \lambda I)^{-1}\Sigma(\hat{\Sigma} + \lambda I)^{-1}\hat{\Sigma}_2(\beta_1 - \beta_2) \\
&\quad + 2\lambda(1-\alpha) \cdot \beta_1^T\hat{\Sigma} + \lambda I)^{-1}\Sigma(\hat{\Sigma} + \lambda I)^{-1}\hat{\Sigma}_2(\beta_1 - \beta_2) \\
&= \lambda^2 \operatorname{Tr}((\hat{\Sigma} + \lambda I)^{-1}\Sigma(\hat{\Sigma} + \lambda I)^{-1}B^{\mathrm{sn}}) + (1-\alpha)^2 \operatorname{Tr}(\hat{\Sigma}_2(\hat{\Sigma} + \lambda I)^{-1}\Sigma(\hat{\Sigma} + \lambda I)^{-1}\hat{\Sigma}_2 B^{\mathrm{df}}) \\
&\quad + 2\lambda(1-\alpha) \cdot \operatorname{Tr}((\hat{\Sigma} + \lambda I)^{-1}\Sigma(\hat{\Sigma} + \lambda I)^{-1}\hat{\Sigma}_2 B^{\mathrm{mx}}).
\end{aligned}$$

as desired. □

Outline for the rest of this Appendix. The bulk of our analysis in this section boils down to analyzing Term 1 (T1), Term 2 (T2), and Term 3 (T3) in Claim 14. Our main technical tool is the random matrix machinery from Appendix 5. In Appendix 4.A, we provide useful sublemmas about intermediate deterministic equivalents that we apply to analyze Terms 2 and 3. We then analyze Term 1 (Appendix 4.B), Term 2 (Appendix 4.C), and Term 3 (Appendix 4.D), and use this to prove Lemma 6(Appendix 4.E).

We apply the power scaling assumptions to derive a simpler expression for the deterministic equivalent (Lemma 26 in Appendix 4.F). We then apply Lemma 26 to prove Theorem 7(Appendix 4.G), and we prove Corollary 8(Appendix 4.H). We also apply Lemma 26 to prove Theorem 9(Appendix 4.I), and we prove Corollary 10(Appendix 4.J). We defer auxiliary calculations to Appendix 4.K.

4.A. Useful lemmas about intermediate deterministic equivalents. The results in this section consider $Z_1 := \frac{\alpha}{1-\alpha}\hat{\Sigma}_1 + \frac{\lambda}{1-\alpha}I$, which we introduce when conditioning on the randomness of $\hat{\Sigma}_1$ when analyzing (T2) and (T3). We derive several properties of Z_1 and the effective regularizer $\kappa_1 = \kappa(1, N(1-\alpha), Z_1^{-1/2}\Sigma Z_1^{-1/2})$ below.

The first set of lemmas relate the trace of various matrices involving κ_1 and Z_1 to deterministic quantities. A subtlety is that κ_1 and Z_1 are correlated, so we cannot directly apply Marčenko-Pastur, and instead we must indirectly analyze this quantity.

Lemma 15. *Consider the setup of Lemma 6, and assume the notation above. Assume $\alpha < 1$. Let $Z_1 = \frac{\alpha}{1-\alpha}\hat{\Sigma}_1 + \frac{\lambda}{1-\alpha}I$, and let $\kappa_1 = \kappa(1, N(1-\alpha), Z_1^{-1/2}\Sigma Z_1^{-1/2})$. Suppose that B has bounded operator norm.*

$$\kappa_1 \operatorname{Tr}\left((\Sigma + \kappa_1 Z_1)^{-1} B\right) \sim \frac{(1-\alpha)\kappa}{\lambda} \operatorname{Tr}\left((\Sigma + \kappa I)^{-1} B\right)$$

Proof. By Claim 19, we know that:

$$\begin{aligned}(1-\alpha)\operatorname{Tr}\left(\left(\hat{\Sigma} + \lambda I\right)^{-1} B\right) &= \operatorname{Tr}\left(\left(\hat{\Sigma}_2 + Z_1\right)^{-1} B\right) \\ &\sim_{(A)} \kappa_1 \operatorname{Tr}\left((\Sigma + \kappa_1 I)^{-1} B\right).\end{aligned}$$

where (A) applies Lemma 36 and Claim 20.

Furthermore, by Lemma 36, it holds that:

$$\lambda \operatorname{Tr}\left(\left(\hat{\Sigma} + \lambda I\right)^{-1} B\right) \sim \kappa \operatorname{Tr}\left((\Sigma + \kappa I)^{-1} B\right).$$

Putting this all together yields the desired result. ☐

Lemma 16. *Consider the setup of Lemma 6, and assume the notation above. Assume $\alpha < 1$. Let $Z_1 = \frac{\alpha}{1-\alpha}\hat{\Sigma}_1 + \frac{\lambda}{1-\alpha}I$, and let $\kappa_1 = \kappa(1, N(1-\alpha), Z_1^{-1/2}\Sigma Z_1^{-1/2})$. Suppose that A and B have bounded operator norm. Then it holds that:*

$$(\kappa_1)^2\left(\operatorname{Tr}\left((\Sigma + \kappa_1 Z_1)^{-1} A (\Sigma + \kappa_1 Z_1)^{-1} B\right) + E_1\right) \sim \frac{(1-\alpha)^2\kappa^2}{\lambda^2}\left(\operatorname{Tr}\left((\Sigma + \kappa I)^{-1} A (\Sigma + \kappa I)^{-1} B\right) + E_2\right)$$

where

$$\begin{aligned}\kappa &= \kappa(\lambda, N, \Sigma) \\ E_1 &= \frac{\frac{1}{N(1-\alpha)}\operatorname{Tr}(A(\Sigma + \kappa_1 Z_1)^{-1}\Sigma(\Sigma + \kappa_1 Z_1)^{-1})}{1 - \frac{1}{N(1-\alpha)}\operatorname{Tr}((\Sigma + \kappa_1 Z_1)^{-1})\Sigma(\Sigma + \kappa_1 Z_1)^{-1}\Sigma} \cdot \operatorname{Tr}\left((\Sigma + \kappa_1 Z_1)^{-1}\Sigma(\Sigma + \kappa_1 Z_1)^{-1} B\right) \\ E_2 &= \frac{\frac{1}{N}\operatorname{Tr}(A\Sigma(\Sigma + \kappa I)^{-2})}{1 - \frac{1}{N}\operatorname{Tr}(\Sigma^2(\Sigma + \kappa I)^{-2})} \cdot \operatorname{Tr}\left((\Sigma + \kappa I)^{-1}\Sigma(\Sigma + \kappa I)^{-1} B\right)\end{aligned}$$

Proof. By Claim 19, we know that:

$$\begin{aligned}(1-\alpha)^2 \operatorname{Tr}\left(\left(\hat{\Sigma} + \lambda I\right)^{-1} A \left(\hat{\Sigma} + \lambda I\right)^{-1} B\right) &= \operatorname{Tr}\left(\left(\hat{\Sigma}_2 + Z_1\right)^{-1} A \left(\hat{\Sigma}_2 + Z_1\right)^{-1} B\right) \\ &\sim_{(A)} \kappa_1^2\left(\operatorname{Tr}\left((\Sigma + \kappa_1 Z_1)^{-1} A (\Sigma + \kappa_1 Z_1)^{-1} B\right) + E_1\right).\end{aligned}$$

where (A) applies Lemma 36 and Claim 20.

Furthermore, by Lemma 36, it holds that:

$$\lambda^2 \operatorname{Tr}\left(\left(\hat{\Sigma} + \lambda I\right)^{-1} A \left(\hat{\Sigma} + \lambda I\right)^{-1} B\right) \sim \kappa^2\left(\operatorname{Tr}\left((\Sigma + \kappa I)^{-1} A (\Sigma + \kappa I)^{-1} B\right) + E_2\right).$$

Putting this all together yields the desired result.

☐

Lemma 17. *Consider the setup of Lemma 6, and assume the notation above. Assume $\alpha < 1$. Let $Z_1 = \frac{\alpha}{1-\alpha}\hat{\Sigma}_1 + \frac{\lambda}{1-\alpha}I$, and let $\kappa_1 = \kappa(1, N(1-\alpha), Z_1^{-1/2}\Sigma Z_1^{-1/2})$. Then it holds that:*

$$\kappa_1^2 \frac{\mathrm{Tr}((\Sigma + \kappa_1 Z_1)^{-1}\Sigma(\Sigma + \kappa_1 Z_1)^{-1}\Sigma)}{1 - \frac{1}{N(1-\alpha)}\mathrm{Tr}((\Sigma + \kappa_1 Z_1)^{-1}\Sigma(\Sigma + \kappa_1 Z_1)^{-1}\Sigma)} \sim \frac{(1-\alpha)^2\kappa^2}{\lambda^2}\frac{\mathrm{Tr}(\Sigma^2(\Sigma + \kappa I)^{-2})}{1 - \frac{1}{N}\mathrm{Tr}(\Sigma^2(\Sigma+\kappa I)^{-2})}$$

Proof. By Claim 19, we know that:

$$\begin{aligned}
&(1-\alpha)^2 \,\mathrm{Tr}\left(\left(\hat{\Sigma} + \lambda I\right)^{-1}\Sigma\left(\hat{\Sigma} + \lambda I\right)^{-1}\Sigma\right) \\
&= \mathrm{Tr}\left(\left(\hat{\Sigma}_2 + Z_1\right)^{-1}\Sigma\left(\hat{\Sigma}_2 + Z_1\right)^{-1}\Sigma\right) \\
&\sim_{(A)} \kappa_1^2\left(1 + \frac{\frac{1}{N(1-\alpha)}\mathrm{Tr}((\Sigma + \kappa_1 Z_1)^{-1}\Sigma(\Sigma + \kappa_1 Z_1)^{-1}\Sigma)}{1 - \frac{1}{N(1-\alpha)}\mathrm{Tr}((\Sigma + \kappa_1 Z_1)^{-1}\Sigma(\Sigma + \kappa_1 Z_1)^{-1}\Sigma)}\right)\mathrm{Tr}\left((\Sigma + \kappa_1 Z_1)^{-1}\Sigma\left(\Sigma + \kappa_1 Z_1\right)^{-1}\Sigma\right) \\
&= \kappa_1^2 \frac{\mathrm{Tr}((\Sigma + \kappa_1 Z_1)^{-1}\Sigma(\Sigma + \kappa_1 Z_1)^{-1}\Sigma)}{1 - \frac{1}{N(1-\alpha)}\mathrm{Tr}((\Sigma + \kappa_1 Z_1)^{-1}\Sigma(\Sigma + \kappa_1 Z_1)^{-1}\Sigma)}
\end{aligned}$$

where (A) applies Lemma 36 and Claim 20.

Furthermore, by Lemma 36, it holds that:

$$\begin{aligned}
&\lambda^2 \,\mathrm{Tr}\left(\left(\hat{\Sigma} + \lambda I\right)^{-1}\Sigma\left(\hat{\Sigma} + \lambda I\right)^{-1}\Sigma\right) \\
&\sim_{(A)} \kappa^2\left(1 + \frac{\frac{1}{N}\mathrm{Tr}(\Sigma^2(\Sigma + \kappa I)^{-2})}{1 - \frac{1}{N}\mathrm{Tr}(\Sigma^2(\Sigma + \kappa I)^{-2}}\right)\mathrm{Tr}\left((\Sigma + \kappa I)^{-1}\Sigma\left(\Sigma + \kappa I\right)^{-1}\Sigma\right) \\
&= \kappa^2\left(\frac{\mathrm{Tr}\left(\Sigma^2\left(\Sigma + \kappa I\right)^{-2}\right)}{1 - \frac{1}{N}\mathrm{Tr}(\Sigma^2(\Sigma + \kappa I)^{-2}}\right).
\end{aligned}$$

where (A) applies Lemma 36.

Putting this all together yields the desired result. □

Next, we relate the random effective regularizer κ_1 to the deterministic effective regularizer $\kappa(\lambda, N, \Sigma)$.

Lemma 18. *Consider the setup of Lemma 6, and assume the notation above. Assume $\alpha < 1$. Let $Z_1 = \frac{\alpha}{1-\alpha}\hat{\Sigma}_1 + \frac{\lambda}{1-\alpha}I$, and let $\kappa_1 = \kappa(1, N(1-\alpha), Z_1^{-1/2}\Sigma Z_1^{-1/2})$. Let $\kappa = \kappa(\lambda, N, \Sigma)$. Then, it holds that $\lambda\kappa_1 \sim \kappa$.*

Proof. Recall that $\kappa_1 = \kappa(1, N(1-\alpha), Z_1^{-1/2}\Sigma Z_1^{-1/2})$ is the unique value such that:

$$\frac{1}{\kappa_1} + \frac{1}{N(1-\alpha)}\mathrm{Tr}((Z_1^{-1/2}\Sigma Z_1^{-1/2} + \kappa_1 I)^{-1}Z_1^{-1/2}\Sigma Z_1^{-1/2}) = 1.$$

We can write this as:

$$1 + \frac{\kappa_1}{N(1-\alpha)}\mathrm{Tr}((\Sigma + \kappa_1 Z_1)^{-1}\Sigma) = \kappa_1.$$

Now we apply Lemma 15 to see that:

$$\kappa_1 = 1 + \frac{\kappa_1}{N(1-\alpha)}\mathrm{Tr}((\Sigma + \kappa_1 Z_1)^{-1}\Sigma) \sim 1 + \frac{1}{N(1-\alpha)}\frac{(1-\alpha)\kappa}{\lambda}\mathrm{Tr}((\Sigma + \kappa I)^{-1}\Sigma).$$

We can write this to see that:

$$\kappa_1 \sim \frac{\kappa}{\lambda}\left(\frac{\lambda}{\kappa} + \frac{1}{N}\mathrm{Tr}((\Sigma + \kappa I)^{-1}\Sigma)\right) = \frac{\kappa}{\lambda}.$$

This implies that $\lambda\kappa_1 \sim \kappa$ as desired.

□

The proofs of these results relied on the following facts.

Claim 19. *Consider the setup of Lemma 6, and assume the notation above. Assume $\alpha < 1$. Let $Z_1 = \frac{\alpha}{1-\alpha}\hat{\Sigma}_1 + \frac{\lambda}{1-\alpha}I$. Then it holds that:*

$$(\hat{\Sigma} + \lambda I)^{-1} = (1-\alpha)^{-1}(\hat{\Sigma}_2 + Z_1)^{-1}.$$

Proof. We observe that:

$$(1-\alpha)(\hat{\Sigma}+\lambda I)^{-1} = (1-\alpha)(\alpha\hat{\Sigma}_1 + (1-\alpha)\hat{\Sigma}_2 + \lambda I)^{-1}$$
$$= (1-\alpha)(1-\alpha)^{-1}\left(\hat{\Sigma}_2 + \frac{\alpha}{1-\alpha}\hat{\Sigma}_1 + \frac{\lambda}{1-\alpha}I\right)^{-1}$$
$$= \left(\hat{\Sigma}_2 + Z_1\right)^{-1},$$

where $Z_1 = \frac{\alpha}{1-\alpha}\hat{\Sigma}_1 + \frac{\lambda}{1-\alpha}I$. □

Claim 20. *Consider the setup of Lemma 6, and assume the notation above. Assume $\alpha < 1$. Let $Z_1 = \frac{\alpha}{1-\alpha}\hat{\Sigma}_1 + \frac{\lambda}{1-\alpha}I$. Then it holds that Z_1 and Z_1^{-1} both have bounded operator norm.*

Proof. Since $\hat{\Sigma}_1$ is PSD, we observe that:

$$\|Z_1\|_{op} = \frac{\alpha}{1-\alpha}\|\hat{\Sigma}_1\|_{op} + \frac{\lambda}{1-\alpha}.$$

The fact that $\|\hat{\Sigma}_1\|_{op}$ is bounded follows from the boundedness requirements from Assumption 1. This proves that $\|Z_1\|_{op}$ is bounded.

To see that $\|Z_1^{-1}\|$ is also bounded, note that:

$$\|Z_1^{-1}\|_{op} \geq \frac{1-\alpha}{\lambda}$$

□

4.B. Analysis of Term 1 (T1). We show the following deterministic equivalent for term 1. This analysis is identical to the analysis of the deterministic equivalent for single-objective linear regression (2, 3), and we include it for completeness.

Lemma 21. *Consider the setup of Lemma 6, and assume the notation above. Then it holds that:*

$$\lambda^2 \operatorname{Tr}((\hat{\Sigma}+\lambda I)^{-1}\Sigma(\hat{\Sigma}+\lambda I)^{-1}B^{sn}) \sim \frac{\kappa^2}{1-\frac{1}{N}\operatorname{Tr}(\Sigma^2(\Sigma+\kappa I)^{-2})}\cdot \operatorname{Tr}(\Sigma(\Sigma+\kappa I)^{-2}B^{sn})$$

Proof. We apply Lemma 36 to see that:

$$\lambda^2 \operatorname{Tr}((\hat{\Sigma}+\lambda I)^{-1}\Sigma(\hat{\Sigma}+\lambda I)^{-1}B^{\text{sn}})$$
$$\sim \kappa^2 \operatorname{Tr}((\Sigma+\kappa I)^{-1}\Sigma(\Sigma+\kappa I)^{-1}B^{\text{sn}}) + \frac{\frac{1}{N}\operatorname{Tr}(\Sigma^2(\Sigma+\kappa I)^{-2})}{1-\frac{1}{N}\operatorname{Tr}(\Sigma^2(\Sigma+\kappa I)^{-2})}$$
$$= \frac{\kappa^2}{1-\frac{1}{N}\operatorname{Tr}(\Sigma^2(\Sigma+\kappa I)^{-2})}\cdot\operatorname{Tr}(\Sigma(\Sigma+\kappa I)^{-2}B^{\text{sn}}),$$

as desired. □

4.C. Analysis of Term 2 (T2). We show the following deterministic equivalent for term 2.

Lemma 22. *Consider the setup of Lemma 6, and assume the notation above. Then it holds that:*

$$(1-\alpha)^2 \operatorname{Tr}\left(\hat{\Sigma}_2(\hat{\Sigma}+\lambda I)^{-1}\Sigma(\hat{\Sigma}+\lambda I)^{-1}\hat{\Sigma}_2 B^{df}\right)$$
$$\sim \frac{(1-\alpha)^2}{1-\frac{1}{N}\operatorname{Tr}(\Sigma^2(\Sigma+\kappa I)^{-2})}\left(\operatorname{Tr}\left((\Sigma+\kappa I)^{-1}\Sigma(\Sigma+\kappa I)^{-1}\Sigma B^{df}\Sigma\right)\right)$$
$$+\frac{(1-\alpha)\frac{1}{N}\operatorname{Tr}(\Sigma^2(\Sigma+\kappa I)^{-2})}{1-\frac{1}{N}\operatorname{Tr}(\Sigma^2(\Sigma+\kappa I)^{-2})}\cdot\left(\operatorname{Tr}\left(\Sigma B^{df}\right) - 2(1-\alpha)\operatorname{Tr}\left((\Sigma+\kappa I)^{-1}\Sigma B^{df}\Sigma\right)\right)$$

The key idea of the proof is to unwrap the randomness in layers. First, we condition on $\hat{\Sigma}_1$ and replace the randomness $\hat{\Sigma}_2$ with a deterministic equivalent where the effective regularizer κ_1 depends on $\hat{\Sigma}_1$ (Lemma 23). At this stage, we unfortunately cannot directly deal with the randomness $\hat{\Sigma}_1$ with deterministic equivalence due to the presence of terms κ_1 which depend on $\hat{\Sigma}_1$, and we instead apply the sublemmas from the previous section.

The following lemma replaces the randomness $\hat{\Sigma}_2$ with a deterministic equivalent.

Lemma 23. *Consider the setup of Lemma 6, and assume the notation above. Assume that $\alpha < 1$. Let $Z_1 = \frac{\alpha}{1-\alpha}\hat{\Sigma}_1 + \frac{\lambda}{1-\alpha}I$, and let $\kappa_1 = \kappa(1, N(1-\alpha), Z_1^{-1/2}\Sigma Z_1^{-1/2})$. Then it holds that:*

$$(1-\alpha)^2 \operatorname{Tr}\left(\hat{\Sigma}_2(\hat{\Sigma}+\lambda I)^{-1}\Sigma(\hat{\Sigma}+\lambda I)^{-1}\hat{\Sigma}_2 B^{df}\right)$$
$$\sim \frac{\operatorname{Tr}\left((\Sigma+\kappa_1 Z_1)^{-1}\Sigma(\Sigma+\kappa_1 Z_1)^{-1}\Sigma B^{df}\Sigma\right)}{1-\frac{1}{N(1-\alpha)}\operatorname{Tr}((\Sigma+\kappa_1 Z_1)^{-1}\Sigma(\Sigma+\kappa_1 Z_1)^{-1}\Sigma)}$$
$$+\frac{\frac{1}{N(1-\alpha)}\operatorname{Tr}((\Sigma+\kappa_1 Z_1)^{-1}\Sigma(\Sigma+\kappa_1 Z_1)^{-1}\Sigma)}{1-\frac{1}{N(1-\alpha)}\operatorname{Tr}((\Sigma+\kappa_1 Z_1)^{-1}\Sigma(\Sigma+\kappa_1 Z_1)^{-1}\Sigma)}\cdot\left(\operatorname{Tr}\left(\Sigma B^{df}\right) - 2\operatorname{Tr}\left(\Sigma(\Sigma+\kappa_1 Z_1)^{-1}\Sigma B^{df}\right)\right).$$

Proof. By Claim 19 we have that:

$$\begin{aligned}(1-\alpha)^2 \operatorname{Tr}\left(\hat{\Sigma}_2(\hat{\Sigma}+\lambda I)^{-1}\Sigma(\hat{\Sigma}+\lambda I)^{-1}\hat{\Sigma}_2 B^{\text{df}}\right) &= \operatorname{Tr}\left(\hat{\Sigma}_2\left(\hat{\Sigma}_2+Z_1\right)^{-1}\Sigma\left(\hat{\Sigma}_2+Z_1\right)^{-1}\hat{\Sigma}_2 B^{\text{df}}\right) \\ &\sim_{(A)} \operatorname{Tr}\left(\Sigma(\Sigma+\kappa_1 Z_1)^{-1}\Sigma(\Sigma+\kappa_1 Z_1)^{-1}\Sigma B^{\text{df}}\right) + E \\ &= \operatorname{Tr}\left((\Sigma+\kappa_1 Z_1)^{-1}\Sigma(\Sigma+\kappa_1 Z_1)^{-1}\Sigma B^{\text{df}}\Sigma\right) + E\end{aligned}$$

where (A) follows from Lemma 39 and Claim 20, and E is defined such that

$$E := \frac{\frac{1}{N(1-\alpha)}\operatorname{Tr}((\Sigma+\kappa_1 Z_1)^{-1}\Sigma(\Sigma+\kappa_1 Z_1)^{-1}\Sigma)}{1-\frac{1}{N(1-\alpha)}\operatorname{Tr}(\Sigma+\kappa_1 Z_1)^{-1}\Sigma(\Sigma+\kappa_1 Z_1)^{-1}\Sigma)} \cdot (\kappa_1)^2 \operatorname{Tr}\left(Z_1\left(\Sigma+\kappa_1 Z_1\right)^{-1}\Sigma\left(\Sigma+\kappa_1 Z_1\right)^{-1} Z_1 B^{\text{df}}\right).$$

and $\kappa_1 = \kappa(\lambda, N(1-\alpha), Z_1^{-1/2}\Sigma Z_1^{-1/2})$.

Note that:

$$\begin{aligned}&(\kappa_1)^2 \operatorname{Tr}\left(Z_1\left(\Sigma+\kappa_1 Z_1\right)^{-1}\Sigma\left(\Sigma+\kappa_1 Z_1\right)^{-1} Z_1 B^{\text{df}}\right) \\ &= \operatorname{Tr}\left((\kappa_1 Z_1)\left(\Sigma+\kappa_1 Z_1\right)^{-1}\Sigma\left(\Sigma+\kappa_1 Z_1\right)^{-1}(\kappa_1 Z_1) B^{\text{df}}\right) \\ &= \operatorname{Tr}\left(\left(I-\Sigma(\Sigma+\kappa_1 Z_1)^{-1}\right)\Sigma\left(I-\Sigma(\Sigma+\kappa_1 Z_1)^{-1}\right)^T B^{\text{df}}\right) \\ &= \operatorname{Tr}\left(\Sigma B^{\text{df}}\right) - 2\operatorname{Tr}\left((\Sigma+\kappa_1 Z_1)^{-1}\Sigma B^{\text{df}}\Sigma\right) + \operatorname{Tr}\left((\Sigma+\kappa_1 Z_1)^{-1}\Sigma(\Sigma+\kappa_1 Z_1)^{-1}\Sigma B^{\text{df}}\Sigma\right).\end{aligned}$$

Note that:

$$\begin{aligned}&\operatorname{Tr}\left((\Sigma+\kappa_1 Z_1)^{-1}\Sigma(\Sigma+\kappa_1 Z_1)^{-1}\Sigma B^{\text{df}}\Sigma\right) \\ &+ \operatorname{Tr}\left((\Sigma+\kappa_1 Z_1)^{-1}\Sigma(\Sigma+\kappa_1 Z_1)^{-1}\Sigma B^{\text{df}}\Sigma\right) \cdot \frac{\frac{1}{N(1-\alpha)}\operatorname{Tr}((\Sigma+\kappa_1 Z_1)^{-1}\Sigma(\Sigma+\kappa_1 Z_1)^{-1}\Sigma)}{1-\frac{1}{N(1-\alpha)}\operatorname{Tr}(\Sigma+\kappa_1 Z_1)^{-1}\Sigma(\Sigma+\kappa_1 Z_1)^{-1}\Sigma)} \\ &= \frac{\operatorname{Tr}\left((\Sigma+\kappa_1 Z_1)^{-1}\Sigma(\Sigma+\kappa_1 Z_1)^{-1}\Sigma B^{\text{df}}\Sigma\right)}{1-\frac{1}{N(1-\alpha)}\operatorname{Tr}(\Sigma+\kappa_1 Z_1)^{-1}\Sigma(\Sigma+\kappa_1 Z_1)^{-1}\Sigma)}\end{aligned}$$

□

Now we are ready to prove Lemma 22.

Proof of Lemma 22. The statement follows trivially if $\alpha = 1$. By Lemma 23, it holds that:

$$\begin{aligned}&(1-\alpha)^2 \operatorname{Tr}\left(\hat{\Sigma}_2(\hat{\Sigma}+\lambda I)^{-1}\Sigma(\hat{\Sigma}+\lambda I)^{-1}\hat{\Sigma}_2 B^{\text{df}}\right) \\ &\sim \frac{\operatorname{Tr}\left((\Sigma+\kappa_1 Z_1)^{-1}\Sigma\left(\Sigma+\kappa_1 Z_1\right)^{-1}\Sigma B^{\text{df}}\Sigma\right)}{1-\frac{1}{N(1-\alpha)}\operatorname{Tr}((\Sigma+\kappa_1 Z_1)^{-1}\Sigma(\Sigma+\kappa_1 Z_1)^{-1}\Sigma)} \\ &+ \frac{\frac{1}{N(1-\alpha)}\operatorname{Tr}((\Sigma+\kappa_1 Z_1)^{-1}\Sigma(\Sigma+\kappa_1 Z_1)^{-1}\Sigma)}{1-\frac{1}{N(1-\alpha)}\operatorname{Tr}((\Sigma+\kappa_1 Z_1)^{-1}\Sigma(\Sigma+\kappa_1 Z_1)^{-1}\Sigma)} \cdot \left(\operatorname{Tr}\left(\Sigma B^{\text{df}}\right) - 2\operatorname{Tr}\left((\Sigma+\kappa_1 Z_1)^{-1}\Sigma B^{\text{df}}\Sigma\right)\right) \\ &\sim_{(A)} (1-\alpha)^2\left(\operatorname{Tr}\left((\Sigma+\kappa I)^{-1}\Sigma\left(\Sigma+\kappa I\right)^{-1}\Sigma B^{\text{df}}\Sigma\right)\right) \\ &+ \frac{\frac{1}{N}\operatorname{Tr}(\Sigma^2(\Sigma+\kappa I)^{-2})}{1-\frac{1}{N}\operatorname{Tr}(\Sigma^2(\Sigma+\kappa I)^{-2})} \cdot (1-\alpha)^2 \cdot \operatorname{Tr}\left((\Sigma+\kappa I)^{-1}\Sigma(\Sigma+\kappa I)^{-1}\Sigma B^{\text{df}}\Sigma\right) \\ &+ \frac{\frac{1}{N(1-\alpha)}\operatorname{Tr}((\Sigma+\kappa_1 Z_1)^{-1}\Sigma(\Sigma+\kappa_1 Z_1)^{-1}\Sigma)}{1-\frac{1}{N(1-\alpha)}\operatorname{Tr}((\Sigma+\kappa_1 Z_1)^{-1}\Sigma(\Sigma+\kappa_1 Z_1)^{-1}\Sigma)} \cdot \left(\operatorname{Tr}\left(\Sigma B^{\text{df}}\right) - 2(1-\alpha)\operatorname{Tr}\left((\Sigma+\kappa I)^{-1}\Sigma B^{\text{df}}\Sigma\right)\right) \\ &= \frac{(1-\alpha)^2}{1-\frac{1}{N}\operatorname{Tr}(\Sigma^2(\Sigma+\kappa I)^{-2})}\left(\operatorname{Tr}\left((\Sigma+\kappa I)^{-1}\Sigma\left(\Sigma+\kappa I\right)^{-1}\Sigma B^{\text{df}}\Sigma\right)\right) \\ &+ \frac{\frac{1}{N(1-\alpha)}\operatorname{Tr}(\Sigma^2(\Sigma+\kappa_1 Z_1)^{-2})}{1-\frac{1}{N(1-\alpha)}\operatorname{Tr}(\Sigma^2(\Sigma+\kappa_1 Z_1)^{-2})} \cdot \left(\operatorname{Tr}\left(\Sigma B^{\text{df}}\right) - 2(1-\alpha)\operatorname{Tr}\left((\Sigma+\kappa I)^{-1}\Sigma B^{\text{df}}\Sigma\right)\right) \\ &\sim_{(B)} \frac{(1-\alpha)^2}{1-\frac{1}{N}\operatorname{Tr}(\Sigma^2(\Sigma+\kappa I)^{-2})}\left(\operatorname{Tr}\left((\Sigma+\kappa I)^{-1}\Sigma\left(\Sigma+\kappa I\right)^{-1}\Sigma B^{\text{df}}\Sigma\right)\right) \\ &+ (1-\alpha)\frac{\frac{1}{N}\operatorname{Tr}(\Sigma^2(\Sigma+\kappa I)^{-2})}{1-\frac{1}{N}\operatorname{Tr}(\Sigma^2(\Sigma+\kappa I)^{-2})} \cdot \left(\operatorname{Tr}\left(\Sigma B^{\text{df}}\right) - 2(1-\alpha)\operatorname{Tr}\left((\Sigma+\kappa I)^{-1}\Sigma B^{\text{df}}\Sigma\right)\right)\end{aligned}$$

where (A) applies Lemma 16, Lemma 15, and (B) uses Lemma 17 and Lemma 18.

□

282 **4.D. Analysis of Term 3 (T3).** We show the following deterministic equivalent for term 3.

Lemma 24. *Consider the setup of Lemma 6, and assume the notation above. Let* $B^{mx} = (\beta_1 - \beta_2)\beta_1^T$*, and let* $\kappa = \kappa(\lambda, N, \Sigma)$*. Then it holds that:*

$$\begin{aligned}
&2\lambda(1-\alpha)\operatorname{Tr}\left((\hat{\Sigma}+\lambda I)^{-1}\Sigma(\hat{\Sigma}+\lambda I)^{-1}\hat{\Sigma}_2 B^{mx}\right) \\
&\sim \frac{2(1-\alpha)\kappa}{1-\frac{1}{N}\operatorname{Tr}(\Sigma^2(\Sigma+\kappa I)^{-2})}\operatorname{Tr}\left((\Sigma+\kappa I)^{-1}\Sigma(\Sigma+\kappa I)^{-1}\Sigma B^{mx}\right) \\
&- 2\frac{(1-\alpha)\frac{1}{N}\operatorname{Tr}(\Sigma^2(\Sigma+\kappa I)^{-2})}{1-\frac{1}{N}\operatorname{Tr}(\Sigma^2(\Sigma+\kappa I)^{-2})}\cdot \kappa \operatorname{Tr}\left((\Sigma+\kappa I)^{-1}\Sigma B^{mx}\right)
\end{aligned}$$

283 The analysis follows a similar structure to the analysis of (T2); we similarly unwrap the randomness in layers.

Lemma 25. *Consider the setup of Lemma 6, and assume the notation above. Assume* $\alpha < 1$*. Let* $Z_1 = \frac{\alpha}{1-\alpha}\hat{\Sigma}_1 + \frac{\lambda}{1-\alpha}I$*, and let* $\kappa_1 = \kappa(1, N(1-\alpha), Z_1^{-1/2}\Sigma Z_1^{-1/2})$*. Then it holds that:*

$$\begin{aligned}
&2\lambda(1-\alpha)^2\operatorname{Tr}\left((\hat{\Sigma}+\lambda I)^{-1}\Sigma(\hat{\Sigma}+\lambda I)^{-1}\hat{\Sigma}_2 B^{mx}\right) \\
&\sim 2\frac{\lambda\kappa_1}{(1-\alpha)}\frac{\operatorname{Tr}\left((\Sigma+\kappa_1 Z_1)^{-1}\Sigma(\Sigma+\kappa_1 Z_1)^{-1}\Sigma B^{mx}\right)}{1-\frac{1}{N(1-\alpha)}\operatorname{Tr}((\Sigma+\kappa_1 Z_1)^{-1}\Sigma(\Sigma+\kappa_1 Z_1)^{-1}\Sigma)} \\
&- 2\frac{\lambda\kappa_1}{(1-\alpha)}\cdot\frac{\frac{1}{N(1-\alpha)}\operatorname{Tr}(\Sigma^2(\Sigma+\kappa_1 Z_1)^{-2})}{1-\frac{1}{N(1-\alpha)}\operatorname{Tr}((\Sigma+\kappa_1 Z_1)^{-1}\Sigma(\Sigma+\kappa_1 Z_1)^{-1}\Sigma)}\cdot\operatorname{Tr}\left(\Sigma(\Sigma+\kappa_1 Z_1)^{-1}\Sigma B^{mx}\right).
\end{aligned}$$

Proof. By Claim 19 we have that:

$$\begin{aligned}
2\lambda(1-\alpha)\operatorname{Tr}\left((\hat{\Sigma}+\lambda I)^{-1}\Sigma(\hat{\Sigma}+\lambda I)^{-1}\hat{\Sigma}_2 B^{\mathrm{mx}}\right) &= 2\frac{\lambda}{(1-\alpha)}\operatorname{Tr}\left(\left(\hat{\Sigma}_2+Z_1\right)^{-1}\Sigma\left(\hat{\Sigma}_2+Z_1\right)^{-1}\hat{\Sigma}_2 B^{\mathrm{mx}}\right) \\
&\sim_{(A)} 2\frac{\lambda}{(1-\alpha)}\left(\kappa_1\operatorname{Tr}\left((\Sigma+\kappa_1 Z_1)^{-1}\Sigma(\Sigma+\kappa_1 Z_1)^{-1}\Sigma B^{\mathrm{mx}}\right) - E\right)
\end{aligned}$$

where (A) follows from Lemma 40 and Claim 20, and E is defined such that

$$E := \frac{\frac{1}{N(1-\alpha)}\operatorname{Tr}((\Sigma+\kappa_1 Z_1)^{-1}\Sigma(\Sigma+\kappa_1 Z_1)^{-1}\Sigma)}{1-\frac{1}{N(1-\alpha)}\operatorname{Tr}((\Sigma+\kappa_1 Z_1)^{-1}\Sigma(\Sigma+\kappa_1 Z_1)^{-1}\Sigma)}\cdot(\kappa_1)^2\operatorname{Tr}\left((\Sigma+\kappa_1 Z_1)^{-1}\Sigma\left(\Sigma+\kappa_1 Z_1\right)^{-1}Z_1 B^{\mathrm{mx}}\right).$$

284 and $\kappa_1 = \kappa(\lambda, N(1-\alpha), Z_1^{-1/2}\Sigma Z_1^{-1/2})$.

Note that:

$$\begin{aligned}
&(\kappa_1)^2\operatorname{Tr}\left((\Sigma+\kappa_1 Z_1)^{-1}\Sigma\left(\Sigma+\kappa_1 Z_1\right)^{-1}Z_1 B^{\mathrm{mx}}\right) \\
&= \kappa_1\operatorname{Tr}\left((\Sigma+\kappa_1 Z_1)^{-1}\Sigma\left(\Sigma+\kappa_1 Z_1\right)^{-1}(\kappa_1 Z_1)B^{\mathrm{mx}}\right) \\
&= \kappa_1\operatorname{Tr}\left((\Sigma+\kappa_1 Z_1)^{-1}\Sigma\left(I-(\Sigma+\kappa_1 Z_1)^{-1}\Sigma\right)B^{\mathrm{mx}}\right) \\
&= \kappa_1\operatorname{Tr}\left((\Sigma+\kappa_1 Z_1)^{-1}\Sigma B^{\mathrm{mx}}\right) - \kappa_1\operatorname{Tr}\left((\Sigma+\kappa_1 Z_1)^{-1}\Sigma(\Sigma+\kappa_1 Z_1)^{-1}\Sigma B^{\mathrm{mx}}\right)
\end{aligned}$$

Moreover, note that:

$$\begin{aligned}
&2\frac{\lambda\kappa_1}{(1-\alpha)}\operatorname{Tr}\left((\Sigma+\kappa_1 Z_1)^{-1}\Sigma(\Sigma+\kappa_1 Z_1)^{-1}\Sigma B^{\mathrm{mx}}\right) \\
&+ 2\frac{\lambda}{(1-\alpha)}\cdot\frac{\frac{1}{N(1-\alpha)}\operatorname{Tr}((\Sigma+\kappa_1 Z_1)^{-1}\Sigma(\Sigma+\kappa_1 Z_1)^{-1}\Sigma)}{1-\frac{1}{N(1-\alpha)}\operatorname{Tr}((\Sigma+\kappa_1 Z_1)^{-1}\Sigma(\Sigma+\kappa_1 Z_1)^{-1}\Sigma)}\cdot\kappa_1\operatorname{Tr}\left((\Sigma+\kappa_1 Z_1)^{-1}\Sigma(\Sigma+\kappa_1 Z_1)^{-1}\Sigma B^{\mathrm{mx}}\right) \\
&= 2\frac{\lambda}{(1-\alpha)}\frac{\operatorname{Tr}\left((\Sigma+\kappa_1 Z_1)^{-1}\Sigma(\Sigma+\kappa_1 Z_1)^{-1}\Sigma B^{\mathrm{mx}}\right)}{1-\frac{1}{N(1-\alpha)}\operatorname{Tr}((\Sigma+\kappa_1 Z_1)^{-1}\Sigma(\Sigma+\kappa_1 Z_1)^{-1}\Sigma)}\cdot\kappa_1.
\end{aligned}$$

285 □

286 Now we are ready to prove Lemma 22.

Proof of Lemma 22. The statement follows trivially if $\alpha = 1$. By Lemma 23, it holds that:

$$
\begin{aligned}
&2\lambda(1-\alpha)^2 \operatorname{Tr}\left((\hat{\Sigma}+\lambda I)^{-1}\Sigma(\hat{\Sigma}+\lambda I)^{-1}\hat{\Sigma}_2 B^{\mathtt{mx}}\right)\\
&\sim 2\frac{\lambda\kappa_1}{(1-\alpha)} \frac{\operatorname{Tr}\left((\Sigma+\kappa_1 Z_1)^{-1}\Sigma(\Sigma+\kappa_1 Z_1)^{-1}\Sigma B^{\mathtt{mx}}\right)}{1-\frac{1}{N(1-\alpha)}\operatorname{Tr}((\Sigma+\kappa_1 Z_1)^{-1}\Sigma(\Sigma+\kappa_1 Z_1)^{-1}\Sigma)}\\
&-\frac{\frac{1}{N(1-\alpha)}\operatorname{Tr}((\Sigma+\kappa_1 Z_1)^{-1}\Sigma(\Sigma+\kappa_1 Z_1)^{-1}\Sigma)}{1-\frac{1}{N(1-\alpha)}\operatorname{Tr}((\Sigma+\kappa_1 Z_1)^{-1}\Sigma(\Sigma+\kappa_1 Z_1)^{-1}\Sigma)}\cdot 2\frac{\lambda\kappa_1}{(1-\alpha)}\operatorname{Tr}\left((\Sigma+\kappa_1 Z_1)^{-1}\Sigma B^{\mathtt{mx}}\right)\\
&\sim_{(A)} 2(1-\alpha)\kappa \operatorname{Tr}\left((\Sigma+\kappa I)^{-1}\Sigma(\Sigma+\kappa I)^{-1}\Sigma B^{\mathtt{mx}}\right)\\
&+2(1-\alpha)\kappa\frac{\frac{1}{N}\operatorname{Tr}(\Sigma^2(\Sigma+\kappa I)^{-2})}{1-\frac{1}{N}\operatorname{Tr}(\Sigma^2(\Sigma+\kappa I)^{-2})}\operatorname{Tr}\left((\Sigma+\kappa I)^{-1}\Sigma(\Sigma+\kappa I)^{-1}\Sigma B^{\mathtt{mx}}\right)\\
&-2\frac{\frac{1}{N(1-\alpha)}\operatorname{Tr}((\Sigma+\kappa_1 Z_1)^{-1}\Sigma(\Sigma+\kappa_1 Z_1)^{-1}\Sigma)}{1-\frac{1}{N(1-\alpha)}\operatorname{Tr}((\Sigma+\kappa_1 Z_1)^{-1}\Sigma(\Sigma+\kappa_1 Z_1)^{-1}\Sigma)}\cdot\kappa\operatorname{Tr}\left((\Sigma+\kappa I)^{-1}\Sigma B^{\mathtt{mx}}\right)\\
&=2\frac{(1-\alpha)\kappa}{1-\frac{1}{N}\operatorname{Tr}(\Sigma^2(\Sigma+\kappa I)^{-2})}\operatorname{Tr}\left((\Sigma+\kappa I)^{-1}\Sigma(\Sigma+\kappa I)^{-1}\Sigma B^{\mathtt{mx}}\right)\\
&-2\frac{\frac{1}{N(1-\alpha)}\operatorname{Tr}((\Sigma+\kappa_1 Z_1)^{-1}\Sigma(\Sigma+\kappa_1 Z_1)^{-1}\Sigma)}{1-\frac{1}{N(1-\alpha)}\operatorname{Tr}((\Sigma+\kappa_1 Z_1)^{-1}\Sigma(\Sigma+\kappa_1 Z_1)^{-1}\Sigma)}\cdot\kappa\operatorname{Tr}\left((\Sigma+\kappa I)^{-1}\Sigma B^{\mathtt{mx}}\right)\\
&\sim_{(B)} 2\frac{(1-\alpha)\kappa}{1-\frac{1}{N}\operatorname{Tr}(\Sigma^2(\Sigma+\kappa I)^{-2})}\operatorname{Tr}\left((\Sigma+\kappa I)^{-1}\Sigma(\Sigma+\kappa I)^{-1}\Sigma B^{\mathtt{mx}}\right)\\
&-2(1-\alpha)\frac{\frac{1}{N}\operatorname{Tr}(\Sigma^2(\Sigma+\kappa I)^{-2})}{1-\frac{1}{N}\operatorname{Tr}(\Sigma^2(\Sigma+\kappa I)^{-2})}\cdot\kappa\operatorname{Tr}\left((\Sigma+\kappa I)^{-1}\Sigma B^{\mathtt{mx}}\right)
\end{aligned}
$$

where (A) applies Lemma 16, Lemma 15, and Lemma 18, and (B) uses Lemma 17 and Lemma 18.

□

4.E. Proof of Lemma 6. Lemma 6follows from the sublemmas in this section.

Proof. We apply Claim 14 to decompose the error in terms (T1), (T2), and (T3). We replace these terms with deterministic equivalents using Lemma 21, Lemma 22, and Lemma 24. The statement follows from adding these terms. □

4.F. Reformulation of Lemma 6 using power-law scaling assumptions. Under the power-law scaling assumptions, we show the following:

Lemma 26. *Suppose that power scaling holds for the eigenvalues and alignment coefficients with scaling $\gamma, \delta > 0$ and correlation coefficient $\rho \in [0, 1)$, and suppose that $P = \infty$. Suppose that $\lambda \in (0, 1)$, and $N \geq 1$. Let $L_1^{det} := L_1^{det}(\beta_1, \beta_2, \mathcal{D}_F, \lambda, N, \alpha)$ be the deterministic equivalent from Lemma 6. Let $\kappa = \kappa(\lambda, N, \Sigma)$ from Definition 1. Let $L^*(\rho) = \mathbb{E}_{\mathcal{D}_W}[(\beta_1 - \beta_2)^T \Sigma(\beta_1 - \beta_2)]$. Then it holds that:*

$$
\begin{aligned}
Q\cdot\mathbb{E}_{\mathcal{D}_W}[L_1^{det}] &= \kappa^2(1-2(1-\alpha)^2(1-\rho))\sum_{i=1}^{P}\frac{i^{-\delta-1-\gamma}}{(i^{-1-\gamma}+\kappa)^2}+(1-\alpha)^2 L^*(\rho)\\
&+2\kappa(1-\rho)(1-\alpha)(1-2(1-\alpha))\sum_{i=1}^{P}\frac{i^{-\delta-2(1+\gamma)}}{(i^{-1-\gamma}+\kappa)^2}\\
&+2(1-\alpha)(1-\rho)\frac{1}{N}\left(\sum_{i=1}^{P}\frac{i^{-2-2\gamma}}{(i^{-1-\gamma}+\kappa)^2}\right)\cdot(1-2(1-\alpha))\sum_{i=1}^{P}\frac{i^{-\delta-2-2\gamma}}{i^{-1-\gamma}+\kappa},
\end{aligned}
$$

where $Q = 1 - \frac{1}{N}\sum_{i=1}^{P}\frac{i^{-2-2\gamma}}{(i^{-1-\gamma}+\kappa)^2}$.

Before proving Lemma 26, we prove a number of sublemmas where we analyze each of the terms in Lemma 6, using the power-law scaling assumptions. In the proofs in this section, we use the notation $F \approx F'$ to denote that $F = \Theta(F')$ where the Θ is allowed to hide dependence on the scaling exponents γ and δ. Moreover let $\Sigma = V\Lambda V^T$ be the eigendecomposition of Σ, where Λ is a diagonal matrix consisting of the eigenvalues.

Lemma 27. *Suppose that the power-law scaling assumptions hold with exponents $\gamma, \delta > 0$ and correlation coefficient $\rho \in [0, 1)$, and suppose that $P = \infty$. Assume the notation from Lemma 6. Let $\nu = \min(2(1 + \gamma), \gamma + \delta)$. Then it holds that:*

$$
\mathbb{E}_{\mathcal{D}_W}[T_1] := \kappa^2\cdot\operatorname{Tr}(\Sigma\Sigma_\kappa^{-2}\mathbb{E}_{\mathcal{D}_W}[B^{sn}]) = \kappa^2\sum_{i=1}^{P}\frac{i^{-\delta-1-\gamma}}{(i^{-1-\gamma}+\kappa)^2}.
$$

Proof. Observe that:

$$\begin{aligned}\mathrm{Tr}(\Sigma\Sigma_\kappa^{-2}\mathbb{E}_{\mathcal{D}_W}[B^{\mathrm{sn}}]) &= \mathrm{Tr}(\Lambda(\Lambda+\kappa I)^{-2}\mathbb{E}_{\mathcal{D}_W}[V^T\beta_1\beta_1^T V]) \\ &= \sum_{i=1}^P \frac{i^{-1-\gamma}}{(i^{-1-\gamma}+\kappa)^2}\cdot\mathbb{E}_{\mathcal{D}_W}[\langle\beta_1, v_i\rangle^2] \\ &= \sum_{i=1}^P \frac{i^{-\delta-1-\gamma}}{(i^{-1-\gamma}+\kappa)^2}\end{aligned}$$

□

Lemma 28. *Suppose that the power-law scaling assumptions hold with exponents $\gamma, \delta > 0$ and correlation coefficient $\rho \in [0, 1)$, and suppose that $P = \infty$. Assume the notation from Lemma 6. Then it holds that:*

$$\mathbb{E}_{\mathcal{D}_W}[T_2] := (1-\alpha)^2\left(\mathrm{Tr}\left(\Sigma_\kappa^{-2}\Sigma^3\mathbb{E}_{\mathcal{D}_W}[B^{df}]\right)\right) = 2(1-\alpha)^2(1-\rho)\sum_{i=1}^P\frac{i^{-\delta-3(1+\gamma)}}{(i^{-1-\gamma}+\kappa)^2}.$$

Proof. First, we observe that

$$\mathbb{E}_{\mathcal{D}_W}[\langle\beta_1-\beta_2, v_i\rangle^2] = \mathbb{E}_{\mathcal{D}_W}[\langle\beta_1, v_i\rangle^2] + \mathbb{E}_{\mathcal{D}_W}[\langle\beta_2, v_i\rangle^2] - 2\mathbb{E}_{\mathcal{D}_W}[\langle\beta_1, v_i\rangle\langle\beta_2, v_i\rangle] = i^{-\delta}+i^{-\delta}-2\rho i^{-\delta} = 2(1-\rho)i^{-\delta}.$$

It is easy to see that:

$$\begin{aligned}\mathrm{Tr}\left(\Sigma_\kappa^{-2}\Sigma^3\mathbb{E}_{\mathcal{D}_W}[B^{\mathrm{df}}]\right) &= \mathrm{Tr}(\Lambda^3(\Lambda+\kappa I)^{-2}\mathbb{E}_{\mathcal{D}_W}[V^T(\beta_1-\beta_2)(\beta_1-\beta_2)^T V]) \\ &= \sum_{i=1}^P\frac{i^{-3(1+\gamma)}}{(i^{-1-\gamma}+\kappa)^2}\cdot\mathbb{E}_{\mathcal{D}_W}[\langle\beta_1-\beta_2, v_i\rangle^2] \\ &= 2(1-\rho)\sum_{i=1}^P\frac{i^{-\delta-3(1+\gamma)}}{(i^{-1-\gamma}+\kappa)^2}.\end{aligned}$$

□

Lemma 29. *Suppose that the power-law scaling assumptions hold with exponents $\gamma, \delta > 0$ and correlation coefficient $\rho \in [0, 1)$, and suppose that $P = \infty$. Assume the notation from Lemma 6. Then it holds that:*

$$\mathbb{E}_{\mathcal{D}_W}[T_3] := 2(1-\alpha)\kappa\cdot\mathrm{Tr}\left(\Sigma_\kappa^{-2}\Sigma^2 B^{mx}\right) = 2(1-\alpha)\kappa(1-\rho)\sum_{i=1}^P\frac{i^{-\delta-2-2\gamma}}{(i^{-1-\gamma}+\kappa)^2}.$$

Proof. First, we observe that

$$\mathbb{E}_{\mathcal{D}_W}[\langle\beta_1-\beta_2, v_i\rangle\langle\beta_1, v_i\rangle] = \mathbb{E}_{\mathcal{D}_W}[\langle\beta_1, v_i\rangle^2] - \mathbb{E}_{\mathcal{D}_W}[\langle\beta_1, v_i\rangle\langle\beta_2, v_i\rangle] = i^{-\delta} - \rho i^{-\delta} = (1-\rho)i^{-\delta}.$$

Observe that:

$$\begin{aligned}\mathrm{Tr}\left(\Sigma_\kappa^{-2}\Sigma^2 B^{\mathrm{mx}}\right) &= \mathrm{Tr}(\Lambda^2(\Lambda+\kappa I)^{-2}\mathbb{E}_{\mathcal{D}_W}[V^T(\beta_1-\beta_2)\beta_1^T V]) \\ &= \sum_{i=1}^P\frac{i^{-2(1+\gamma)}}{(i^{-1-\gamma}+\kappa)^2}\cdot\mathbb{E}_{\mathcal{D}_W}[\langle\beta_1-\beta_2, v_i\rangle\langle\beta_1, v_i\rangle] \\ &= (1-\rho)\sum_{i=1}^P\frac{i^{-\delta-2-2\gamma}}{(i^{-1-\gamma}+\kappa)^2}.\end{aligned}$$

This means that:

$$\mathbb{E}_{\mathcal{D}_W}[T_3] = 2(1-\alpha)\kappa(1-\rho)\sum_{i=1}^P\frac{i^{-\delta-2-2\gamma}}{(i^{-1-\gamma}+\kappa)^2}.$$

□

Lemma 30. *Suppose that the power-law scaling assumptions hold with exponents $\gamma, \delta > 0$ and correlation coefficient $\rho \in [0, 1)$, and suppose that $P = \infty$. Assume the notation from Lemma 6. Then it holds that:*

$$\begin{aligned}|\mathbb{E}_{\mathcal{D}_W}[T_4]| &:= 2\kappa(1-\alpha)\frac{1}{N}\mathrm{Tr}(\Sigma^2\Sigma_\kappa^{-2})\cdot\mathrm{Tr}\left(\Sigma_\kappa^{-1}\Sigma\mathbb{E}_{\mathcal{D}_W}[B^{mx}]\right) \\ &= 2\kappa(1-\alpha)(1-\rho)\frac{1}{N}\left(\sum_{i=1}^P\frac{i^{-2-2\gamma}}{(i^{-1-\gamma}+\kappa)^2}\right)\left(\sum_{i=1}^P\frac{i^{-\delta-1-\gamma}}{i^{-1-\gamma}+\kappa}\right)\end{aligned}$$

Proof. First, we observe that

$$\mathbb{E}_{\mathcal{D}_W}[\langle \beta_1 - \beta_2, v_i \rangle \langle \beta_1, v_i \rangle] = \mathbb{E}_{\mathcal{D}_W}[\langle \beta_1, v_i \rangle^2] - \mathbb{E}_{\mathcal{D}_W}[\langle \beta_1, v_i \rangle \langle \beta_2, v_i \rangle] = i^{-\delta} + -\rho i^{-\delta} = (1-\rho) i^{-\delta}.$$

Observe that:

$$\begin{aligned} \operatorname{Tr}\left(\Sigma_\kappa^{-1} \Sigma \mathbb{E}_{\mathcal{D}_W}[B^{\mathtt{mx}}]\right) &= \operatorname{Tr}(\Lambda(\Lambda + \kappa I)^{-1} \mathbb{E}_{\mathcal{D}_W}[V^T (\beta_1 - \beta_2) \beta_1^T V]) \\ &= \sum_{i=1}^P \frac{i^{-1-\gamma}}{i^{-1-\gamma} + \kappa} \cdot \mathbb{E}_{\mathcal{D}_W}[\langle \beta_1 - \beta_2, v_i \rangle \langle \beta_1, v_i \rangle] \\ &= (1-\rho) \sum_{i=1}^P \frac{i^{-\delta-1-\gamma}}{i^{-1-\gamma} + \kappa}. \end{aligned}$$

Now, apply Lemma 32, we see that:

$$\begin{aligned} |\mathbb{E}_{\mathcal{D}_W}[T_4]| &:= 2\kappa(1-\alpha) \frac{1}{N} \operatorname{Tr}(\Sigma^2 \Sigma_\kappa^{-2}) \cdot \operatorname{Tr}\left(\Sigma_\kappa^{-1} \Sigma B^{\mathtt{mx}}\right) \\ &=_{(A)} 2\kappa(1-\alpha)(1-\rho) \frac{1}{N} \left(\sum_{i=1}^P \frac{i^{-2-2\gamma}}{(i^{-1-\gamma} + \kappa)^2} \right) \left(\sum_{i=1}^P \frac{i^{-\delta-1-\gamma}}{i^{-1-\gamma} + \kappa} \right) \end{aligned}$$

where (A) follows from Lemma 32. ☐

Lemma 31. *Suppose that the power-law scaling assumptions hold with exponents $\gamma, \delta > 0$ and correlation coefficient $\rho \in [0, 1)$, and suppose that $P = \infty$. Assume the notation from Lemma 6, and similarly let*

$$\begin{aligned} \mathbb{E}_{\mathcal{D}_W}[T_5] &:= (1-\alpha) \frac{1}{N} \operatorname{Tr}(\Sigma^2 \Sigma_\kappa^{-2}) \cdot \left(\operatorname{Tr}\left(\Sigma \mathbb{E}_{\mathcal{D}_W}[B^{\mathtt{df}}]\right) - 2(1-\alpha) \operatorname{Tr}\left(\Sigma_\kappa^{-1} \Sigma^2 \mathbb{E}_{\mathcal{D}_W}[B^{\mathtt{df}}]\right)\right) \\ &= 2(1-\alpha)(1-\rho) \frac{1}{N} \left(\sum_{i=1}^P \frac{i^{-2-2\gamma}}{(i^{-1-\gamma} + \kappa)^2} \right) \cdot \left(\sum_{i=1}^P i^{-\delta-1-\gamma} - 2(1-\alpha) \cdot \sum_{i=1}^P \frac{i^{-\delta-2-2\gamma}}{(i^{-1-\gamma} + \kappa)} \right). \end{aligned}$$

Proof. First, we observe that

$$\mathbb{E}_{\mathcal{D}_W}[\langle \beta_1 - \beta_2, v_i \rangle^2] = \mathbb{E}_{\mathcal{D}_W}[\langle \beta_1, v_i \rangle^2] + \mathbb{E}_{\mathcal{D}_W}[\langle \beta_2, v_i \rangle^2] - 2\mathbb{E}_{\mathcal{D}_W}[\langle \beta_1, v_i \rangle \langle \beta_2, v_i \rangle] = i^{-\delta} + i^{-\delta} - 2\rho i^{-\delta} = 2(1-\rho) i^{-\delta}.$$

Now, observe that:

$$\begin{aligned} \mathbb{E}_{\mathcal{D}_W}[T_5] &:= (1-\alpha) \frac{1}{N} \operatorname{Tr}(\Sigma^2 \Sigma_\kappa^{-2}) \cdot \left(\operatorname{Tr}\left(\Sigma \mathbb{E}_{\mathcal{D}_W}[B^{\mathtt{df}}]\right) - 2(1-\alpha) \operatorname{Tr}\left(\Sigma_\kappa^{-1} \Sigma^2 \mathbb{E}_{\mathcal{D}_W}[B^{\mathtt{df}}]\right)\right) \\ &= (1-\alpha) \frac{1}{N} \operatorname{Tr}(\Sigma^2 \Sigma_\kappa^{-2}) \cdot \left(\operatorname{Tr}\left(\Lambda \mathbb{E}_{\mathcal{D}_W}[V^T (\beta_1 - \beta_2)(\beta_1 - \beta_2)^T V]\right)\right) \\ &- (1-\alpha) \frac{1}{N} \operatorname{Tr}(\Sigma^2 \Sigma_\kappa^{-2}) \cdot \left(2(1-\alpha) \operatorname{Tr}\left((\Lambda + \kappa I)^{-1} \Lambda^2 \mathbb{E}_{\mathcal{D}_W}[V^T (\beta_1 - \beta_2)(\beta_1 - \beta_2)^T V]\right)\right) \\ &= (1-\alpha) \frac{1}{N} \operatorname{Tr}(\Sigma^2 \Sigma_\kappa^{-2}) \cdot \left(\sum_{i=1}^P i^{-1-\gamma} \langle \beta_1 - \beta_2, v_i \rangle^2 - 2(1-\alpha) \cdot \sum_{i=1}^P \frac{i^{-2-2\gamma}}{(i^{-1-\gamma} + \kappa)} \langle \beta_1 - \beta_2, v_i \rangle^2 \right) \\ &= 2(1-\alpha)(1-\rho) \frac{1}{N} \operatorname{Tr}(\Sigma^2 \Sigma_\kappa^{-2}) \cdot \left(\sum_{i=1}^P i^{-\delta-1-\gamma} - 2(1-\alpha) \cdot \sum_{i=1}^P \frac{i^{-\delta-2-2\gamma}}{(i^{-1-\gamma} + \kappa)} \right) \\ &=_{(A)} 2(1-\alpha)(1-\rho) \frac{1}{N} \left(\sum_{i=1}^P \frac{i^{-2-2\gamma}}{(i^{-1-\gamma} + \kappa)^2} \right) \cdot \left(\sum_{i=1}^P i^{-\delta-1-\gamma} - 2(1-\alpha) \cdot \sum_{i=1}^P \frac{i^{-\delta-2-2\gamma}}{(i^{-1-\gamma} + \kappa)} \right). \end{aligned}$$

where (A) uses Lemma 32. ☐

The proofs of these sublemmas use the following fact.

Lemma 32. *Suppose that the power-law scaling assumptions hold with exponents $\gamma, \delta > 0$ and correlation coefficient $\rho \in [0, 1)$, and suppose that $P = \infty$. Assume the notation from Lemma 6. Then it holds that:*

$$\operatorname{Tr}\left(\Sigma^2 (\Sigma + \kappa I)^{-2}\right) = \sum_{i=1}^P \frac{i^{-2-2\gamma}}{(i^{-1-\gamma} + \kappa)^2}.$$

Proof. We see that:

$$
\begin{aligned}
(1-\alpha)\frac{1}{N}\operatorname{Tr}(\Sigma^2\Sigma_\kappa^{-2}) &= (1-\alpha)\frac{1}{N}\operatorname{Tr}(V\Lambda^2(\Lambda+\kappa I)^{-2}V^T) \\
&= (1-\alpha)\frac{1}{N}\operatorname{Tr}(\Lambda^2(\Lambda+\kappa I)^{-2}) \\
&= \sum_{i=1}^{P}\frac{i^{-2-2\gamma}}{(i^{-1-\gamma}+\kappa)^2}.
\end{aligned}
$$

□

Now, we are ready to prove Lemma 26.

Proof of Lemma 26. By Lemma 32, we know:

$$
Q = 1-\frac{1}{N}\operatorname{Tr}(\Sigma^2(\Sigma+\kappa I)^{-2}) = 1-\frac{1}{N}\sum_{i=1}^{P}\frac{i^{-2-2\gamma}}{(i^{-1-\gamma}+\kappa)^2}.
$$

Moreover, we have that:

$$
\begin{aligned}
Q\cdot\mathbb{E}_{\mathcal{D}_W}[L_1^{\mathsf{det}}] &=_{(A)} \mathbb{E}_{\mathcal{D}_W}[T_1+T_2+T_3+T_4+T_5] \\
&=_{(B)} \kappa^2\sum_{i=1}^{P}\frac{i^{-\delta-1-\gamma}}{(i^{-1-\gamma}+\kappa)^2} + 2(1-\alpha)^2(1-\rho)\sum_{i=1}^{P}\frac{i^{-\delta-3(1+\gamma)}}{(i^{-1-\gamma}+\kappa)^2} \\
&+ 2\kappa(1-\rho)(1-\alpha)\sum_{i=1}^{P}\frac{i^{-\delta-2(1+\gamma)}}{(i^{-1-\gamma}+\kappa)^2} \\
&- 2\kappa(1-\rho)(1-\alpha)\frac{1}{N}\left(\sum_{i=1}^{P}\frac{i^{-2-2\gamma}}{(i^{-1-\gamma}+\kappa)^2}\right)\left(\sum_{i=1}^{P}\frac{i^{-\delta-1-\gamma}}{i^{-1-\gamma}+\kappa}\right) \\
&+ 2(1-\alpha)(1-\rho)\frac{1}{N}\left(\sum_{i=1}^{P}\frac{i^{-2-2\gamma}}{(i^{-1-\gamma}+\kappa)^2}\right)\cdot\left(\sum_{i=1}^{P}i^{-\delta-1-\gamma} - 2(1-\alpha)\cdot\sum_{i=1}^{P}\frac{i^{-\delta-2-2\gamma}}{(i^{-1-\gamma}+\kappa)}\right).
\end{aligned}
$$

where (A) follows from Lemma 6, and (B) follows from Lemmas 27-31.

By Claim 13, we know that:

$$
L^*(\rho) = 2(1-\rho)\sum_{i=1}^{P}i^{-\delta-1-\gamma} = 2(1-\rho)\sum_{i=1}^{P}\frac{i^{-\delta-3(1+\gamma)}}{(i^{-1-\gamma})^2}.
$$

This means that:

$$
\begin{aligned}
&L^*(\rho) - 2(1-\rho)\sum_{i=1}^{P}\frac{i^{-\delta-3(1+\gamma)}}{(i^{-1-\gamma}+\kappa)^2} \\
&= 2(1-\rho)\sum_{i=1}^{P}\left(\frac{i^{-\delta-3(1+\gamma)}}{(i^{-1-\gamma})^2} - \frac{i^{-\delta-3(1+\gamma)}}{(i^{-1-\gamma}+\kappa)^2}\right) \\
&= 2(1-\rho)\sum_{i=1}^{P}\left(\frac{i^{-\delta-3(1+\gamma)}\cdot((i^{-1-\gamma}+\kappa)^2-(i^{-1-\gamma})^2)}{(i^{-1-\gamma})^2\cdot(i^{-1-\gamma}+\kappa)^2}\right) \\
&= 2\kappa^2(1-\rho)\sum_{i=1}^{P}\left(\frac{i^{-\delta-3(1+\gamma)}}{(i^{-1-\gamma})^2\cdot(i^{-1-\gamma}+\kappa)^2}\right) + 4\kappa(1-\rho)\sum_{i=1}^{P}\left(\frac{i^{-\delta-3(1+\gamma)}\cdot i^{-1-\gamma}}{(i^{-1-\gamma})^2\cdot(i^{-1-\gamma}+\kappa)^2}\right) \\
&= 2\kappa^2(1-\rho)\sum_{i=1}^{P}\left(\frac{i^{-\delta-1-\gamma}}{(i^{-1-\gamma}+\kappa)^2}\right) + 4\kappa(1-\rho)\sum_{i=1}^{P}\left(\frac{i^{-\delta-2(1+\gamma)}}{(i^{-1-\gamma}+\kappa)^2}\right)
\end{aligned}
$$

Applying this and some other algebraic manipulations, we obtain that:

$$
\begin{aligned}
Q \cdot L_1^{\mathsf{det}} &= \kappa^2(1-2(1-\alpha)^2(1-\rho)) \sum_{i=1}^{P} \frac{i^{-\delta-1-\gamma}}{(i^{-1-\gamma}+\kappa)^2} + (1-\alpha)^2 L^*(\rho) \\
&+ 2\kappa(1-\rho)(1-\alpha)(1-2(1-\alpha)) \sum_{i=1}^{P} \frac{i^{-\delta-2(1+\gamma)}}{(i^{-1-\gamma}+\kappa)^2} \\
&- 2(1-\alpha)(1-\rho)\frac{1}{N}\left(\sum_{i=1}^{P} \frac{i^{-2-2\gamma}}{(i^{-1-\gamma}+\kappa)^2}\right)\left(\sum_{i=1}^{P} i^{-\delta-1-\gamma} - \sum_{i=1}^{P} \frac{i^{-\delta-2-2\gamma}}{i^{-1-\gamma}+\kappa}\right) \\
&+ 2(1-\alpha)(1-\rho)\frac{1}{N}\left(\sum_{i=1}^{P} \frac{i^{-2-2\gamma}}{(i^{-1-\gamma}+\kappa)^2}\right)\cdot\left(\sum_{i=1}^{P} i^{-\delta-1-\gamma} - 2(1-\alpha)\cdot\sum_{i=1}^{P} \frac{i^{-\delta-2-2\gamma}}{(i^{-1-\gamma}+\kappa)}\right) \\
&= \kappa^2(1-2(1-\alpha)^2(1-\rho)) \sum_{i=1}^{P} \frac{i^{-\delta-\gamma}}{(i^{-1-\gamma}+\kappa)^2} + (1-\alpha)^2 L^*(\rho) \\
&+ 2\kappa(1-\rho)(1-\alpha)(1-2(1-\alpha)) \sum_{i=1}^{P} \frac{i^{-\delta-2(1+\gamma)}}{(i^{-1-\gamma}+\kappa)^2} \\
&+ 2(1-\alpha)(1-\rho)\frac{1}{N}\left(\sum_{i=1}^{P} \frac{i^{-2-2\gamma}}{(i^{-1-\gamma}+\kappa)^2}\right)\cdot(1-2(1-\alpha)) \sum_{i=1}^{P} \frac{i^{-\delta-2-2\gamma}}{i^{-1-\gamma}+\kappa}.
\end{aligned}
$$

□

4.G. Proof of Theorem 7. We now prove Theorem 7. In the proof, we again use the notation $F \approx F'$ to denote $F = \Theta(F')$. The main ingredient is Lemma 26, coupled with the auxiliary calculations in Appendix 4.K. Strictly speaking, we need to apply Lemma 26 to a fixed $\Gamma \in (0, \infty) > 0$ (which captures the limit of P/N as specified in Assumption 1)) and then take a limit as $\Gamma \to \infty$. It is easy to verify that this does not affect the calculations below.

Proof. The proof boils down to three steps: (1) obtaining an exact expression, (2) obtaining an up-to-constants asymptotic expression in terms of κ and Q, and (3) substituting in κ and Q.

Step 1: Exact expression. We apply Lemma 26 to see that:

$$
\begin{aligned}
Q \cdot L_1^{\mathsf{det}} &= \kappa^2(1-2(1-\alpha)^2(1-\rho)) \sum_{i=1}^{P} \frac{i^{-\delta-1-\gamma}}{(i^{-1-\gamma}+\kappa)^2} + (1-\alpha)^2 L^*(\rho) \\
&+ 2\kappa(1-\rho)(1-\alpha)(1-2(1-\alpha)) \sum_{i=1}^{P} \frac{i^{-\delta-2(1+\gamma)}}{(i^{-1-\gamma}+\kappa)^2} \\
&+ 2(1-\alpha)(1-\rho)\frac{1}{N}\left(\sum_{i=1}^{P} \frac{i^{-2-2\gamma}}{(i^{-1-\gamma}+\kappa)^2}\right)\cdot(1-2(1-\alpha)) \sum_{i=1}^{P} \frac{i^{-\delta-2-2\gamma}}{i^{-1-\gamma}+\kappa},
\end{aligned}
$$

where $Q = 1 - \frac{1}{N}\sum_{i=1}^{P} \frac{i^{-2-2\gamma}}{(i^{-1-\gamma}+\kappa)^2}$, where $L^*(\rho) = \mathbb{E}_{\mathcal{D}_W}[(\beta_1 - \beta_2)^T \Sigma (\beta_1 - \beta_2)]$, and where $\kappa = \kappa(\Sigma, N, \lambda)$ as defined in Definition 1.

Step 2: Asymptotic expression in terms of κ and Q. We show that

$$
Q \cdot L_1^{\mathsf{det}} \approx \kappa^{\frac{\nu}{1+\gamma}} + (1-\alpha)^2(1-\rho) + (1-\alpha)(1-\rho)\frac{\kappa^{-\frac{1}{1+\gamma}}}{N}.
$$

We analyze this expression term-by-term and repeatedly apply Lemma 33. We see that:

$$
\kappa^2(1-2(1-\alpha)^2(1-\rho)) \sum_{i=1}^{P} \frac{i^{-\delta-1-\gamma}}{(i^{-1-\gamma}+\kappa)^2} \approx_{(A)} \kappa^{\frac{\nu}{1+\gamma}}(1-2(1-\alpha)^2(1-\rho)) \approx_{(B)} \kappa^{\frac{\nu}{1+\gamma}},
$$

where (A) uses Lemma 33 and (B) uses that $\alpha \geq 0.5$. Moreover, we observe that:

$$
(1-\alpha)^2 L^*(\rho) \approx_{(C)} (1-\alpha)^2(1-\rho),
$$

where (C) uses Claim 13. Moreover, we see that:

$$\begin{aligned}
2\kappa(1-\rho)(1-\alpha)(1-2(1-\alpha))\sum_{i=1}^{P}\frac{i^{-\delta-2(1+\gamma)}}{(i^{-1-\gamma}+\kappa)^2} &\approx_{(D)} (1-\alpha)(1-\rho)(1-2(1-\alpha))\max\left(\kappa,\kappa^{\frac{\delta+\gamma}{1+\gamma}}\right)\\
&=_{(E)} O\left((1-\alpha)\sqrt{1-\rho}\max\left(\kappa,\kappa^{\frac{\delta+\gamma}{2(1+\gamma)}}\right)\right)\\
&= O\left(\sqrt{(1-\alpha)^2(1-\rho)\cdot\kappa^{\frac{\min(2(1+\gamma),\gamma+\delta)}{1+\gamma}}}\right)\\
&=_{(F)} O\left(\kappa^{\frac{\min(2(1+\gamma),\gamma+\delta)}{1+\gamma}}+(1-\alpha)^2(1-\rho)\right)\\
&= O\left(\kappa^{\frac{\nu}{1+\gamma}}+(1-\alpha)^2(1-\rho)\right)
\end{aligned}$$

where (D) uses Lemma 33, (E) uses that $1-\rho\leq 1$ and that $\kappa=O(1)$ (which follows from Lemma 35 and the assumption that $\lambda\in(0,1)$) and (F) follows from AM-GM. Finally, observe that:

$$\begin{aligned}
&2(1-\alpha)(1-\rho)\frac{1}{N}\left(\sum_{i=1}^{P}\frac{i^{-2-2\gamma}}{(i^{-1-\gamma}+\kappa)^2}\right)\cdot(1-2(1-\alpha))\sum_{i=1}^{P}\frac{i^{-\delta-2-2\gamma}}{i^{-1-\gamma}+\kappa}\\
&\approx (1-2(1-\alpha))\cdot(1-\alpha)(1-\rho)\frac{1}{N}\left(\sum_{i=1}^{P}\frac{i^{-2-2\gamma}}{(i^{-1-\gamma}+\kappa)^2}\right)\sum_{i=1}^{P}\frac{i^{-\delta-2-2\gamma}}{i^{-1-\gamma}+\kappa}\\
&\approx_{(G)} (1-2(1-\alpha))\cdot(1-\alpha)(1-\rho)\frac{\kappa^{-\frac{1}{1+\gamma}}}{N}
\end{aligned}$$

where (G) uses Lemma 33 twice.

Putting this all together, we see that:

$$Q\cdot L_1^{\mathsf{det}}\approx\kappa^{\frac{\nu}{1+\gamma}}+(1-\alpha)^2(1-\rho)+(1-2(1-\alpha))\cdot(1-\alpha)(1-\rho)\frac{\kappa^{-\frac{1}{1+\gamma}}}{N}.$$

We split into two cases based on α. When $\alpha\geq 0.75$, we observe that

$$(1-2(1-\alpha))\cdot(1-\alpha)(1-\rho)\frac{\kappa^{-\frac{1}{1+\gamma}}}{N}\approx(1-\alpha)(1-\rho)\frac{\kappa^{-\frac{1}{1+\gamma}}}{N},$$

and when $\alpha\in[0.5,0.75]$, we observe that

$$(1-2(1-\alpha))\cdot(1-\alpha)(1-\rho)\frac{\kappa^{-\frac{1}{1+\gamma}}}{N}=O\left((1-\alpha)(1-\rho)\frac{\kappa^{-\frac{1}{1+\gamma}}}{N}\right)$$

and

$$(1-\alpha)^2(1-\rho)\approx_{(H)}(1-\alpha)(1-\rho)\frac{\kappa^{-\frac{1}{1+\gamma}}}{N}$$

where (H) follows from the fact that $\kappa=\Omega(N^{-1-\gamma})$ by Lemma 35. Altogether, this implies that:

$$Q\cdot L_1^{\mathsf{det}}\approx\kappa^{\frac{\nu}{1+\gamma}}+(1-\alpha)^2(1-\rho)+(1-\alpha)(1-\rho)\frac{\kappa^{-\frac{1}{1+\gamma}}}{N},$$

as desired.

Step 2: Substitute in κ and Q. Finally, we apply Lemma 34 to see that:

$$Q^{-1}=\left(1-\frac{1}{N}\sum_{i=1}^{P}\frac{i^{-2-2\gamma}}{(i^{-1-\gamma}+\kappa)^2}\right)^{-1}=\Theta(1).$$

We apply Lemma 35 to see that

$$\kappa=\kappa(\Sigma,N,\Sigma)=\max(N^{-1-\gamma},\lambda).$$

Plugging this into the expression derived in Step 2, we obtain the desired expression. □

4.H. Proof of Corollary 8. We prove Corollary 8using Theorem 7.

Proof. We apply Theorem 7to see that:

$$\mathbb{E}_{\mathcal{D}_W}[L_1^{\mathtt{det}}] = \Theta\left(\underbrace{\max(\lambda^{\frac{\nu}{1+\gamma}}, N^{-\nu})}_{\text{finite-data error}} + \underbrace{(1-\alpha)^2 \cdot (1-\rho)}_{\text{mixture error}} + \underbrace{(1-\alpha)\left(\frac{\min(\lambda^{-\frac{1}{1+\gamma}}, N)}{N}\right)(1-\rho)}_{\text{overfitting error}}\right).$$

We split into three cases: $N \leq (1-\alpha)^{-\frac{1}{\nu}}(1-\rho)^{-\frac{1}{\nu}}$, $(1-\alpha)^{-\frac{1}{\nu}}(1-\rho)^{-\frac{1}{\nu}} \leq N \leq (1-\alpha)^{-\frac{2+\nu}{\nu}}(1-\rho)^{-\frac{1}{\nu}}$, and $N \geq (1-\alpha)^{-\frac{2+\nu}{\nu}}(1-\rho)^{-\frac{1}{\nu}}$.

Case 1: $N \leq (1-\alpha)^{-\frac{1}{\nu}}(1-\rho)^{-\frac{1}{\nu}}$**.** We observe that the finite-data error dominates regardless of λ. This is because the condition implies that

$$\max(\lambda^{\frac{\nu}{1+\gamma}}, N^{-\nu}) \geq (1-\alpha)(1-\rho),$$

which dominates both the mixture error and the overfitting error.

Case 2: $(1-\alpha)^{-\frac{1}{\nu}}(1-\rho)^{-\frac{1}{\nu}} \leq N \leq (1-\alpha)^{-\frac{2+\nu}{\nu}}(1-\rho)^{-\frac{1}{\nu}}$**.** We show that the finite error term and overfitting error dominate. Let $\tilde{N} = \min(\lambda^{-\frac{1}{1+\gamma}}, N)$. We can bound the sum of the finite-data error and the overfitting error as:

$$\max(\lambda^{\frac{\nu}{1+\gamma}}, N^{-\nu}) + (1-\alpha)\left(\frac{\min(\lambda^{-\frac{1}{1+\gamma}}, N)}{N}\right)(1-\rho) = \tilde{N}^{-\nu} + (1-\alpha)(1-\rho)\frac{\tilde{N}}{N}.$$

Taking a derivative (and verifying the second order condition), we see that this expression is minimized when:

$$\nu \cdot \tilde{N}^{-\nu-1} = \frac{(1-\alpha)(1-\rho)}{N}$$

which solves to:

$$\tilde{N} = \Theta\left(\left(\frac{(1-\alpha)(1-\rho)}{N}\right)^{-\frac{1}{1+\nu}}\right).$$

The lower bound on N guarantees that:

$$\tilde{N} = \Theta\left(\left(\frac{(1-\alpha)(1-\rho)}{N}\right)^{-\frac{1}{1+\nu}}\right) = O\left(\left((1-\alpha)^{1+\frac{1}{\nu}}(1-\rho)^{1+\frac{1}{\nu}}\right)^{-\frac{1}{1+\nu}}\right) = O\left((1-\alpha)^{-\frac{1}{\nu}}(1-\rho)^{-\frac{1}{\nu}}\right) = O(N)$$

which ensures that $\tilde{N}$ can be achieved by some choice of λ. In particular, we can take $\lambda = \Theta\left(\left(\frac{(1-\alpha)(1-\rho)}{N}\right)^{\frac{1+\gamma}{\nu+1}}\right)$.

The resulting sum of the finite error and the overfitting error is:

$$\max(\lambda^{\frac{\nu}{1+\gamma}}, N^{-\nu}) + (1-\alpha)\left(\frac{\min(\lambda^{-\frac{1}{1+\gamma}}, N)}{N}\right) = \Theta\left(\left(\frac{(1-\alpha)(1-\rho)}{N}\right)^{\frac{\nu}{\nu+1}}\right).$$

The upper bound on N guarantees that this dominates the mixture error:

$$\Theta\left(\left(\frac{(1-\alpha)(1-\rho)}{N}\right)^{\frac{\nu}{\nu+1}}\right) = \Omega\left(\left((1-\alpha)^{1+\frac{2+\nu}{\nu}}(1-\rho)^{1+\frac{1}{\nu}}\right)^{\frac{\nu}{\nu+1}}\right) = \Omega((1-\alpha)^2(1-\rho))$$

as desired.

Case 3: $N \geq (1-\alpha)^{-\frac{2+\nu}{\nu}}(1-\rho)^{-\frac{1}{\nu}}$**.** We show that the mixture and the overfitting error terms dominate. First, we observe that the sum of the mixture error and the finite-data error is:

$$(1-\alpha)^2(1-\rho) + (1-\alpha)\left(\frac{\min(\lambda^{-\frac{1}{1+\gamma}}, N)}{N}\right)(1-\rho) = \Theta\left((1-\alpha)(1-\rho)\left(1-\alpha+\frac{\min(\lambda^{-\frac{1}{1+\gamma}}, N)}{N}\right)\right).$$

This is minimized by taking $\lambda = \Theta((N(1-\alpha))^{-1-\gamma})$, which yields $\Theta((1-\alpha)^2(1-\rho))$.

The upper bound on N and the setting of λ guarantees that this term dominates the finite-data error:

$$\max(\lambda^{\frac{\nu}{1+\gamma}}, N^{-\nu}) = O((N(1-\alpha))^{-\nu}) \leq O\left((1-\alpha)^{-\nu}(1-\alpha)^{2+\nu}(1-\rho)\right) = O((1-\alpha)^2(1-\rho),$$

as desired.

□

4.I. Proof of Theorem 9. We prove Theorem 9. Like the proof of Theorem 7, strictly speaking, we need to apply Lemma 26 to a fixed $\Gamma \in (0, \infty) > 0$ (which captures the limit of P/N as specified in Assumption 1) and then take a limit as $\Gamma \to \infty$. It is easy to verify that this does not affect the calculations below.

Proof of Theorem 9. Like the proof of Theorem 7, the proof boils down to three steps: (1) obtaining an exact expression, (2) obtaining an up-to-constants asymptotic expression in terms of κ, and (3) substituting in κ.

Step 1: Exact expression. We first apply Lemma 26 to obtain the precise loss:

$$\begin{aligned} Q \cdot \mathbb{E}_{\mathcal{D}_W}[L_1^*(\beta_1, \beta_2, \mathcal{D}_F, \lambda_E, N, \alpha_E)] &= \kappa^2(1 - 2(1-\alpha)^2(1-\rho)) \sum_{i=1}^{P} \frac{i^{-\delta-1-\gamma}}{(i^{-1-\gamma}+\kappa)^2} + (1-\alpha)^2 L^*(\rho) \\ &+ 2\kappa(1-\rho)(1-\alpha)(1-2(1-\alpha)) \sum_{i=1}^{P} \frac{i^{-\delta-2(1+\gamma)}}{(i^{-1-\gamma}+\kappa)^2} \\ &+ 2(1-\alpha)(1-\rho)\frac{1}{N}\left(\sum_{i=1}^{P} \frac{i^{-2-2\gamma}}{(i^{-1-\gamma}+\kappa)^2}\right) \cdot (1-2(1-\alpha)) \sum_{i=1}^{P} \frac{i^{-\delta-2-2\gamma}}{i^{-1-\gamma}+\kappa}, \end{aligned}$$

where $Q = 1 - \frac{1}{N}\sum_{i=1}^{P} \frac{i^{-2-2\gamma}}{(i^{-1-\gamma}+\kappa)^2}$ and where $\kappa = \kappa(\Sigma, N, \lambda)$ as defined in Definition 1. This can be written as:

$$\begin{aligned} &\mathbb{E}_{\mathcal{D}_W}[L_1^*(\beta_1, \beta_2, \mathcal{D}_F, \lambda_E, N, \alpha_E)] - (1-\alpha)^2 L^*(\rho) \\ &= Q^{-1} \cdot \kappa^2(1 - 2(1-\alpha)^2(1-\rho)) \sum_{i=1}^{P} \frac{i^{-\delta-1-\gamma}}{(i^{-1-\gamma}+\kappa)^2} \\ &+ Q^{-1} \cdot 2\kappa(1-\rho)(1-\alpha)(1-2(1-\alpha)) \sum_{i=1}^{P} \frac{i^{-\delta-2(1+\gamma)}}{(i^{-1-\gamma}+\kappa)^2} \\ &+ Q^{-1} \cdot 2(1-\alpha)(1-\rho)\frac{1}{N}\left(\sum_{i=1}^{P} \frac{i^{-2-2\gamma}}{(i^{-1-\gamma}+\kappa)^2}\right) \cdot (1-2(1-\alpha)) \sum_{i=1}^{P} \frac{i^{-\delta-2-2\gamma}}{i^{-1-\gamma}+\kappa} \\ &+ \frac{1-Q}{Q}(1-\alpha)^2 L^*(\rho). \end{aligned}$$

Step 2: Asymptotic expression in terms of κ. We use the notation $F \approx F'$ to denote that $F = \Theta(F')$. We obtain:

$$\begin{aligned} &\mathbb{E}_{\mathcal{D}_W}[L_1^*(\beta_1, \beta_2, \mathcal{D}_F, \lambda_E, N, \alpha_E)] - (1-\alpha)^2 L^*(\rho) \\ &\approx_{(A)} \kappa^2(1 - 2(1-\alpha)^2(1-\rho)) \sum_{i=1}^{P} \frac{i^{-\delta-1-\gamma}}{(i^{-1-\gamma}+\kappa)^2} \\ &+ \kappa(1-\rho)(1-\alpha)(1-2(1-\alpha)) \sum_{i=1}^{P} \frac{i^{-\delta-2(1+\gamma)}}{(i^{-1-\gamma}+\kappa)^2} \\ &+ (1-\alpha)(1-\rho)\frac{1}{N}\left(\sum_{i=1}^{P} \frac{i^{-2-2\gamma}}{(i^{-1-\gamma}+\kappa)^2}\right) \cdot (1-2(1-\alpha)) \sum_{i=1}^{P} \frac{i^{-\delta-2-2\gamma}}{i^{-1-\gamma}+\kappa} \\ &+ (1-Q)(1-\alpha)^2 L^*(\rho) \\ &\approx_{(B)} \kappa^2 \sum_{i=1}^{P} \frac{i^{-\delta-1-\gamma}}{(i^{-1-\gamma}+\kappa)^2} + \kappa(1-\rho)(1-\alpha) \sum_{i=1}^{P} \frac{i^{-\delta-2(1+\gamma)}}{(i^{-1-\gamma}+\kappa)^2} \\ &+ (1-\alpha)(1-\rho)\frac{1}{N}\left(\sum_{i=1}^{P} \frac{i^{-2-2\gamma}}{(i^{-1-\gamma}+\kappa)^2}\right) \sum_{i=1}^{P} \frac{i^{-\delta-2-2\gamma}}{i^{-1-\gamma}+\kappa} \\ &+ (1-\alpha)^2 L^*(\rho) \cdot \frac{1}{N}\left(\sum_{i=1}^{P} \frac{i^{-2-2\gamma}}{(i^{-1-\gamma}+\kappa)^2}\right). \end{aligned}$$

where (A) uses that Q^{-1} is a constant by Lemma 34 and (B) uses that $\alpha \geq 0.75$ and the definition of Q. Now, using the bounds from Lemma 33, and the bound from Claim 13, we obtain:

$$\begin{aligned}
&\mathbb{E}_{\mathcal{D}_W}[L_1^*(\beta_1, \beta_2, \mathcal{D}_F, \lambda_E, N, \alpha_E)] - (1-\alpha)^2 L^*(\rho) \\
&\approx \kappa^{\frac{\min(2(1+\gamma), \gamma+\delta)}{1+\gamma}} + (1-\rho)(1-\alpha)\max\left(\kappa, \kappa^{\frac{\gamma+\delta}{1+\gamma}}\right) + (1-\alpha)(1-\rho)\frac{\kappa^{-\frac{1}{1+\gamma}}}{N} + \frac{\kappa^{-\frac{1}{1+\gamma}}}{N}(1-\alpha)^2(1-\rho) \\
&\approx \kappa^{\frac{\nu}{1+\gamma}} + (1-\rho)(1-\alpha)\kappa^{\frac{\nu'}{1+\gamma}} + (1-\alpha)(1-\rho)\frac{\kappa^{-\frac{1}{1+\gamma}}}{N}.
\end{aligned}$$

Step 3: Substituting in κ. Finally, we apply Lemma 35 to see that

$$\kappa = \kappa(\Sigma, N, \Sigma) = \max(N^{-1-\gamma}, \lambda).$$

Plugging this into the expression derived in Step 2, we obtain the desired expression. □

4.J. Proof of Corollary 10. We prove Corollary 10using Theorem 9.

Proof. We apply Theorem 7to see that:

$$\begin{aligned}
&\mathbb{E}_{\mathcal{D}_W}[L_1^{\mathsf{det}} - L_1(\beta(\alpha, 0))] \\
&= \Theta\left(\underbrace{\max(\lambda^{\frac{\nu}{1+\gamma}}, N^{-\nu})}_{\text{finite-data error}} + \underbrace{(1-\rho)(1-\alpha)\max(\lambda^{\frac{\nu'}{1+\gamma}}, N^{-\nu'})}_{\text{mixture finite-data error}} + \underbrace{(1-\alpha)\left(\frac{\min(\lambda^{-\frac{1}{1+\gamma}}, N)}{N}\right)(1-\rho)}_{\text{overfitting error}}\right).
\end{aligned}$$

We split into three cases: $N \leq (1-\alpha)^{-\frac{1}{\nu}}(1-\rho)^{-\frac{1}{\nu}}$, $(1-\alpha)^{-\frac{1}{\nu}}(1-\rho)^{-\frac{1}{\nu}} \leq N \leq (1-\alpha)^{-\frac{\nu'+1}{\nu-\nu'}}(1-\rho)^{-\frac{\nu'+1}{\nu-\nu'}}$, and $N \geq (1-\alpha)^{-\frac{\nu'+1}{\nu-\nu'}}(1-\rho)^{-\frac{\nu'+1}{\nu-\nu'}}$.

Case 1: $N \leq (1-\alpha)^{-\frac{1}{\nu}}(1-\rho)^{-\frac{1}{\nu}}$. We observe that the finite-data error dominates regardless of λ. This is because the condition implies that

$$\max(\lambda^{\frac{\nu}{1+\gamma}}, N^{-\nu}) \geq (1-\alpha)(1-\rho),$$

which dominates both the mixture finite-data error and the overfitting error.

Case 2: $(1-\alpha)^{-\frac{1}{\nu}}(1-\rho)^{-\frac{1}{\nu}} \leq N \leq (1-\alpha)^{-\frac{\nu'+1}{\nu-\nu'}}(1-\rho)^{-\frac{\nu'+1}{\nu-\nu'}}$. We show that the finite error term and overfitting error dominate. Let $\tilde{N} = \min(\lambda^{-\frac{1}{1+\gamma}}, N)$. We can bound the sum of the finite-data error and the overfitting error as:

$$\max(\lambda^{\frac{\nu}{1+\gamma}}, N^{-\nu}) + (1-\alpha)\left(\frac{\min(\lambda^{-\frac{1}{1+\gamma}}, N)}{N}\right)(1-\rho) = \tilde{N}^{-\nu} + (1-\alpha)(1-\rho)\frac{\tilde{N}}{N}.$$

Taking a derivative (and verifying the second order condition), we see that this expression is minimized when:

$$\nu \cdot \tilde{N}^{-\nu-1} = \frac{(1-\alpha)(1-\rho)}{N}$$

which solves to:

$$\tilde{N} = \Theta\left(\left(\frac{(1-\alpha)(1-\rho)}{N}\right)^{-\frac{1}{1+\nu}}\right).$$

The lower bound on N guarantees that:

$$\tilde{N} = \Theta\left(\left(\frac{(1-\alpha)(1-\rho)}{N}\right)^{-\frac{1}{1+\nu}}\right) = O\left(\left((1-\alpha)^{1+\frac{1}{\nu}}(1-\rho)^{1+\frac{1}{\nu}}\right)^{-\frac{1}{1+\nu}}\right) = O\left((1-\alpha)^{-\frac{1}{\nu}}(1-\rho)^{-\frac{1}{\nu}}\right) = O(N)$$

which ensures that $\tilde{N}$ can be achieved by some choice of λ. In particular, we can take $\lambda = \Theta\left(\left(\frac{(1-\alpha)(1-\rho)}{N}\right)^{\frac{1+\gamma}{\nu+1}}\right)$.

The resulting sum of the finite error and the overfitting error is:

$$\max(\lambda^{\frac{\nu}{1+\gamma}}, N^{-\nu}) + (1-\alpha)\left(\frac{\min(\lambda^{-\frac{1}{1+\gamma}}, N)}{N}\right) = \Theta\left(\left(\frac{(1-\alpha)(1-\rho)}{N}\right)^{\frac{\nu}{\nu+1}}\right).$$

The upper bound on N and the choice of λ guarantees that this dominates the mixture finite-data error, as shown below:

$$
\begin{aligned}
&(1-\rho)(1-\alpha)\max(\lambda^{\frac{\nu'}{1+\gamma}}, N^{-\nu'}) \\
&= \Theta\left((1-\rho)(1-\alpha)\left(\frac{(1-\alpha)(1-\rho)}{N}\right)^{\frac{\nu'}{\nu+1}}\right) \\
&= \Theta\left(\left(\frac{(1-\alpha)(1-\rho)}{N}\right)^{\frac{\nu}{\nu+1}}(1-\alpha)(1-\rho)\left(\frac{(1-\alpha)(1-\rho)}{N}\right)^{\frac{\nu'-\nu}{\nu+1}}\right) \\
&= \Theta\left(\left(\frac{(1-\alpha)(1-\rho)}{N}\right)^{\frac{\nu}{\nu+1}}(1-\alpha)^{\frac{\nu'+1}{\nu+1}}(1-\rho)^{\frac{\nu'+1}{\nu+1}}N^{\frac{\nu-\nu'}{\nu+1}}\right) \\
&= O\left(\left(\frac{(1-\alpha)(1-\rho)}{N}\right)^{\frac{\nu}{\nu+1}}(1-\alpha)^{\frac{\nu'+1}{\nu+1}}(1-\rho)^{\frac{\nu'+1}{\nu+1}}(1-\alpha)^{-\frac{\nu'+1}{\nu+1}}(1-\rho)^{-\frac{\nu'+1}{\nu+1}}\right) \\
&= O\left(\left(\frac{(1-\alpha)(1-\rho)}{N}\right)^{\frac{\nu}{\nu+1}}\right)
\end{aligned}
$$

as desired.

Case 3: $N \geq (1-\alpha)^{-\frac{\nu'+1}{\nu-\nu'}}(1-\rho)^{-\frac{\nu'+1}{\nu-\nu'}}$**.** We show that the mixture finite-data error and the overfitting error terms dominate. First, we observe that the sum of the mixture error and the finite-data error is:

$$
\begin{aligned}
&(1-\rho)(1-\alpha)\max(\lambda^{\frac{\nu'}{1+\gamma}}, N^{-\nu'}) + (1-\alpha)\left(\frac{\min(\lambda^{-\frac{1}{1+\gamma}}, N)}{N}\right)(1-\rho) \\
&= \Theta\left((1-\alpha)(1-\rho)\left(\lambda^{\frac{\nu'}{1+\gamma}} + \frac{\min(\lambda^{-\frac{1}{1+\gamma}}, N)}{N}\right)\right)
\end{aligned}
$$

This is minimized by taking $\lambda = \Theta(N^{-\frac{1+\gamma}{\nu'+1}})$, which yields $\Theta((1-\alpha)(1-\rho)N^{-\frac{\nu'}{\nu'+1}})$.

The upper bound on N and the setting of λ guarantees that this term dominates the finite-data error:

$$
\begin{aligned}
\max(\lambda^{\frac{\nu}{1+\gamma}}, N^{-\nu}) &= \Theta(N^{-\frac{\nu}{\nu'+1}}) \\
&\leq \Theta\left((1-\alpha)(1-\rho)N^{-\frac{\nu'}{\nu'+1}}(1-\alpha)^{-1}(1-\rho)^{-1}N^{-\frac{\nu-\nu'}{\nu'+1}}\right) \\
&= O\left((1-\alpha)(1-\rho)N^{-\frac{\nu'}{\nu'+1}}(1-\alpha)^{-1}(1-\rho)^{-1}(1-\alpha)(1-\rho)\right) \\
&= O\left((1-\alpha)(1-\rho)N^{-\frac{\nu'}{\nu'+1}}\right)
\end{aligned}
$$

as desired.

□

4.K. Auxiliary calculations under power scaling assumptions. We show the following auxiliary calculations which we use when analyzing the terms in Lemma 6under the power scaling assumptions. Throughout this section, we again use the notation $F \approx F'$ to denote that $F = \Theta(F')$.

Lemma 33. *Suppose that the power-law scaling assumptions hold with exponents $\gamma, \delta > 0$ and correlation coefficient $\rho \in [0, 1)$,*

and suppose that $P = \infty$. Let $\kappa = \kappa(\lambda, N, \Sigma)$ be defined according to Definition 1. Then the following holds:

$$\sum_{i=1}^{P} \frac{i^{-\delta-1-\gamma}}{(i^{-1-\gamma}+\kappa)^2} \approx \kappa^{-2} \kappa^{\frac{\min(2(1+\gamma),\gamma+\delta)}{1+\gamma}}$$

$$\sum_{i=1}^{P} \frac{i^{-\delta-3(1+\gamma)}}{(i^{-1-\gamma}+\kappa)^2} \approx 1$$

$$\sum_{i=1}^{P} \frac{i^{-\delta-2-2\gamma}}{i^{-1-\gamma}+\kappa} \approx 1$$

$$\sum_{i=1}^{P} \frac{i^{-\delta-2(1+\gamma)}}{(i^{-1-\gamma}+\kappa)^2} \approx \max(1, \kappa^{\frac{\delta-1}{1+\gamma}})$$

$$\sum_{i=1}^{P} \frac{i^{-\delta-1-\gamma}}{i^{-1-\gamma}+\kappa} \approx \max(1, \kappa^{\frac{\delta-1}{1+\gamma}})$$

$$\sum_{i=1}^{P} \frac{i^{-2-2\gamma}}{(i^{-1-\gamma}+\kappa)^2} \approx \kappa^{-\frac{1}{1+\gamma}}$$

$$\sum_{i=1}^{P} \frac{i^{-1-\gamma}}{i^{-1-\gamma}+\kappa} \approx \kappa^{-\frac{1}{1+\gamma}}$$

$$\sum_{i=1}^{P} \frac{i^{-1-\gamma}}{(i^{-1-\gamma}+\kappa)^2} \approx \kappa^{-2} \kappa^{\frac{\gamma}{1+\gamma}}$$

Proof. To prove the first statement, observe that:

$$\begin{aligned}
\sum_{i=1}^{P} \frac{i^{-\delta-1-\gamma}}{(i^{-1-\gamma}+\kappa)^2} &= \sum_{i \le \kappa^{-\frac{1}{1+\gamma}}} \frac{i^{-\delta-1-\gamma}}{(i^{-1-\gamma}+\kappa)^2} + \sum_{i \ge \kappa^{-\frac{1}{1+\gamma}}} \frac{i^{-\delta-1-\gamma}}{(i^{-1-\gamma}+\kappa)^2} \\
&\approx \sum_{i \le \kappa^{-\frac{1}{1+\gamma}}} i^{1+\gamma-\delta} + \kappa^{-2} \sum_{i \ge \kappa^{-\frac{1}{1+\gamma}}} i^{-\delta-1-\gamma} \\
&\approx \max(1, \kappa^{-\frac{2+\gamma-\delta}{1+\gamma}}) + \kappa^{-2} \kappa^{\frac{\delta+\gamma}{1+\gamma}} \\
&= \kappa^{-2} \max(\kappa^2, \kappa^{\frac{\gamma+\delta}{1+\gamma}}) + \kappa^{-2} \kappa^{\frac{\delta+\gamma}{1+\gamma}} \\
&\approx \kappa^{-2} \max(\kappa^2, \kappa^{\frac{\gamma+\delta}{1+\gamma}}) \\
&\approx \kappa^{-2} \kappa^{\frac{\min(2(1+\gamma),\gamma+\delta)}{1+\gamma}}.
\end{aligned}$$

To prove the second statement, we use Lemma 35 and the assumption that $\lambda \in (0,1)$ to see $\kappa = \Theta(\max(\lambda, N^{-1-\gamma})) = O(1)$. This means that

$$\sum_{i=1}^{P} \frac{i^{-\delta-3(1+\gamma)}}{(i^{-1-\gamma}+\kappa)^2} = \Omega\left(\sum_{i=1}^{P} i^{-\delta-3(1+\gamma)}\right) = \Omega(1).$$

Moreover, we see that:

$$\sum_{i=1}^{P} \frac{i^{-\delta-3(1+\gamma)}}{(i^{-1-\gamma}+\kappa)^2} = O\left(\sum_{i=1}^{P} \frac{i^{-\delta-3(1+\gamma)}}{(i^{-1-\gamma})^2}\right) = O\left(\sum_{i=1}^{P} i^{-\delta-1-\gamma)}\right) = \Omega(1).$$

To prove the third statement, we use Lemma 35 and the assumption that $\lambda \in (0,1)$ to see $\kappa = \Theta(\max(\lambda, N^{-1-\gamma})) = O(1)$. This means that

$$\sum_{i=1}^{P} \frac{i^{-\delta-2-2\gamma}}{i^{-1-\gamma}+\kappa} = \Omega\left(\sum_{i=1}^{P} i^{-\delta-2(1+\gamma)}\right) = \Omega(1).$$

Moreover, we see that:

$$\sum_{i=1}^{P} \frac{i^{-\delta-2(1+\gamma)}}{i^{-1-\gamma}+\kappa} = O\left(\sum_{i=1}^{P} \frac{i^{-\delta-2(1+\gamma)}}{i^{-1-\gamma}}\right) = O\left(\sum_{i=1}^{P} i^{-\delta-1-\gamma}\right) = O(1).$$

To prove the fourth statement, observe that:

$$
\begin{aligned}
\sum_{i=1}^{P} \frac{i^{-\delta-2-2\gamma}}{(i^{-1-\gamma}+\kappa)^2} &\approx \sum_{i \le \kappa^{-\frac{1}{1+\gamma}}} \frac{i^{-\delta-2-2\gamma}}{(i^{-1-\gamma}+\kappa)^2} + \sum_{i \ge \kappa^{-\frac{1}{1+\gamma}}} \frac{i^{-\delta-2-2\gamma}}{(i^{-1-\gamma}+\kappa)^2} \\
&\approx \sum_{i \le \kappa^{-\frac{1}{1+\gamma}}} i^{-\delta} + \kappa^{-2} \sum_{i \ge \kappa^{-\frac{1}{1+\gamma}}} i^{-\delta-2-2\gamma} \\
&\approx \max(1, \kappa^{-\frac{1-\delta}{1+\gamma}}) + \kappa^{-2} \kappa^{\frac{\delta+1+2\gamma}{1+\gamma}} \\
&\approx \max(1, \kappa^{\frac{\delta-1}{1+\gamma}}).
\end{aligned}
$$

To prove the fifth statement, observe that:

$$
\begin{aligned}
\sum_{i=1}^{P} \frac{i^{-\delta-1-\gamma}}{i^{-1-\gamma}+\kappa} &= \sum_{i \le \kappa^{-\frac{1}{1+\gamma}}} \frac{i^{-\delta-1-\gamma}}{i^{-1-\gamma}+\kappa} + \sum_{i \ge \kappa^{-\frac{1}{1+\gamma}}} \frac{i^{-\delta-1-\gamma}}{i^{-1-\gamma}+\kappa} \\
&\approx \sum_{i \le \kappa^{-\frac{1}{1+\gamma}}} i^{-\delta} + \kappa^{-1} \sum_{i \ge \kappa^{-\frac{1}{1+\gamma}}} i^{-\delta-1-\gamma} \\
&\approx \max(1, \kappa^{-\frac{1-\delta}{1+\gamma}}) + \kappa^{-1} \kappa^{\frac{\delta+\gamma}{1+\gamma}} \\
&\approx \max(1, \kappa^{\frac{\delta-1}{1+\gamma}}).
\end{aligned}
$$

To prove the sixth statement, observe that:

$$
\begin{aligned}
\sum_{i=1}^{P} \frac{i^{-2-2\gamma}}{(i^{-1-\gamma}+\kappa)^2} &= \sum_{i \le \kappa^{-\frac{1}{1+\gamma}}} \frac{i^{-2-2\gamma}}{(i^{-1-\gamma}+\kappa)^2} + \sum_{i \ge \kappa^{-\frac{1}{1+\gamma}}} \frac{i^{-2-2\gamma}}{(i^{-1-\gamma}+\kappa)^2} \\
&\approx \sum_{i \le \kappa^{-\frac{1}{1+\gamma}}} 1 + \kappa^{-2} \sum_{i \ge \kappa^{-\frac{1}{1+\gamma}}} i^{-2-2\gamma} \\
&\approx \kappa^{-\frac{1}{1+\gamma}} + \kappa^{-2} \kappa^{\frac{1+2\gamma}{1+\gamma}} \\
&\approx \kappa^{-\frac{1}{1+\gamma}}.
\end{aligned}
$$

To prove the seventh statement, observe that:

$$
\begin{aligned}
\sum_{i=1}^{P} \frac{i^{-1-\gamma}}{i^{-1-\gamma}+\kappa} &= \sum_{i \le \kappa^{-\frac{1}{1+\gamma}}} \frac{i^{-1-\gamma}}{i^{-1-\gamma}+\kappa} + \sum_{i \ge \kappa^{-\frac{1}{1+\gamma}}} \frac{i^{-1-\gamma}}{i^{-1-\gamma}+\kappa} \\
&\approx \sum_{i \le \kappa^{-\frac{1}{1+\gamma}}} 1 + \kappa^{-1} \sum_{i \ge \kappa^{-\frac{1}{1+\gamma}}} i^{-1-\gamma} \\
&\approx \kappa^{-\frac{1}{1+\gamma}} + \kappa^{-1} \kappa^{\frac{\gamma}{1+\gamma}} \\
&\approx \kappa^{-\frac{1}{1+\gamma}}.
\end{aligned}
$$

To prove the eighth statement, observe that:

$$
\begin{aligned}
\sum_{i=1}^{P} \frac{i^{-1-\gamma}}{(i^{-1-\gamma}+\kappa)^2} &= \sum_{i \le \kappa^{-\frac{1}{1+\gamma}}} \frac{i^{-1-\gamma}}{(i^{-1-\gamma}+\kappa)^2} + \sum_{i \ge \kappa^{-\frac{1}{1+\gamma}}} \frac{i^{-\delta-1-\gamma}}{(i^{-1-\gamma}+\kappa)^2} \\
&\approx \sum_{i \le \kappa^{-\frac{1}{1+\gamma}}} i^{1+\gamma} + \kappa^{-2} \sum_{i \ge \kappa^{-\frac{1}{1+\gamma}}} i^{-1-\gamma} \\
&\approx \max(1, \kappa^{-\frac{2+\gamma}{1+\gamma}}) + \kappa^{-2} \kappa^{\frac{\gamma}{1+\gamma}} \\
&= \kappa^{-2} \max(\kappa^2, \kappa^{\frac{\gamma}{1+\gamma}}) + \kappa^{-2} \kappa^{\frac{\gamma}{1+\gamma}} \\
&\approx \kappa^{-2} \max(\kappa^2, \kappa^{\frac{\gamma}{1+\gamma}}) \\
&\approx \kappa^{-2} \kappa^{\frac{\gamma}{1+\gamma}}
\end{aligned}
$$

$\square$

Lemma 34. *Suppose that the power-law scaling assumptions hold with exponents $\gamma, \delta > 0$ and correlation coefficient $\rho \in [0, 1)$, and suppose that $P = \infty$. Assume the notation from Lemma 6, and similarly let*

$$Q := 1 - \frac{1}{N} \operatorname{Tr}(\Sigma^2 \Sigma_\kappa^{-2}).$$

Then it holds that $Q^{-1} = \Theta(1)$.

Proof. Let $\Sigma = V \Lambda V^T$ be the eigendecomposition of Σ, where Λ is a diagonal matrix consisting of the eigenvalues. By Definition 1, we see that:

$$\frac{\lambda}{\kappa} + \frac{1}{N} \operatorname{Tr}(\Sigma \Sigma_\kappa^{-1}) = 1.$$

This implies that:

$$\begin{aligned} Q &= 1 - \frac{1}{N} \operatorname{Tr}(\Sigma \Sigma_\kappa^{-1}) + \frac{1}{N} \left(\operatorname{Tr}(\Sigma \Sigma_\kappa^{-1}) - \operatorname{Tr}(\Sigma^2 \Sigma_\kappa^{-2}) \right) \\ &= \frac{\lambda}{\kappa} + \frac{1}{N} \left(\operatorname{Tr}(\Sigma \Sigma_\kappa^{-1}) - \operatorname{Tr}(\Sigma^2 \Sigma_\kappa^{-2}) \right). \end{aligned}$$

Observe that:

$$\begin{aligned} \operatorname{Tr}(\Sigma \Sigma_\kappa^{-1}) - \operatorname{Tr}(\Sigma^2 \Sigma_\kappa^{-2}) &= \operatorname{Tr}(\Lambda(\Lambda + \kappa I)^{-1}) - \operatorname{Tr}(\Lambda^2 (\Lambda + \kappa I)^{-2}) \\ &= \sum_{i=1}^{P} \left(\frac{i^{-1-\gamma}}{i^{-1-\gamma} + \kappa} - \frac{i^{-2-\gamma}}{(i^{-1-\gamma} + \kappa)^2} \right) \\ &= \kappa \sum_{i=1}^{P} \frac{i^{-1-\gamma}}{(i^{-1-\gamma} + \kappa)^2}. \end{aligned}$$

This means that:

$$\begin{aligned} Q &= \frac{\lambda}{\kappa} + \frac{\kappa}{N} \sum_{i=1}^{P} \frac{i^{-1-\gamma}}{(i^{-1-\gamma} + \kappa)^2} \\ &\approx_{(A)} \frac{\lambda}{\kappa} + \Theta(\left(\frac{\kappa}{N} \kappa^{-2} \kappa^{\frac{\gamma}{1+\gamma}} \right)) \\ &= \frac{\lambda}{\kappa} + \Theta\left(\frac{\kappa^{-\frac{1}{1+\gamma}}}{N} \right). \end{aligned}$$

where (A) uses Lemma 33.

Case 1: $\kappa = \Theta(\lambda)$. In this case, we see that

$$Q = \frac{\lambda}{\kappa} + \Theta\left(\frac{\kappa^{-\frac{1}{1+\gamma}}}{N} \right) = \Theta(1).$$

This means that $Q^{-1} = \Theta(1)$.

Case 2: $\kappa = \Theta(N^{-1-\gamma})$. In this case, we see that

$$Q = \frac{\lambda}{\kappa} + \Theta\left(\frac{\kappa^{-\frac{1}{1+\gamma}}}{N} \right) = \Omega\left(\frac{\kappa^{-\frac{1}{1+\gamma}}}{N} \right) = \Omega(1).$$

This means that $Q^{-1} = \Theta(1)$.

$\square$

Lemma 35. *Suppose that the power-law scaling assumptions hold with exponents $\gamma, \delta > 0$ and correlation coefficient $\rho \in [0, 1)$, and suppose that $P = \infty$. Then it holds that $\kappa(\lambda, M, \Sigma) = \Theta(\max(\lambda, M^{-1-\gamma}))$.*

Proof. Let $\Sigma = V\Lambda V^T$ be the eigendecomposition of Σ, where Λ is a diagonal matrix consisting of the eigenvalues. Observe that:

$$\begin{aligned}\mathrm{Tr}((\Sigma+\kappa I)^{-1}\Sigma) &= \mathrm{Tr}(\Lambda(\Lambda+\kappa I)^{-1}) \\ &= \sum_{i=1}^{P} \frac{i^{-1-\gamma}}{i^{-1-\gamma}+\kappa} \\ &\approx_{(A)} \kappa^{-\frac{1}{1+\gamma}}.\end{aligned}$$

where (A) follows from Lemma 33. Using Definition 1, we see that for $\kappa = \kappa(\lambda, M, \Sigma)$, it holds that:

$$\frac{\lambda}{\kappa} + \frac{1}{M}\Theta(\kappa^{-1-\gamma}) = 1.$$

This implies that $\kappa = \Theta(\max(\lambda, M^{-1-\gamma}))$ as desired. □

5. Machinery from random matrix theory

In this section, we introduce machinery from random matrix theory that serves as the backbone for our analysis of multi-objective scaling laws in Appendix 4. In Appendix 5.A, we give a recap of known Marčenko-Pastur properties. In Appendix 5.B, we use these known properties to derive random matrix theory results which are tailored to our analysis.

5.A. Recap of Marčenko-Pastur properties. We introduce Marčenko-Pastur properties, following the treatment in (3). Informally speaking, Marčenko-Pastur laws show that a random matrix $(\hat{\Sigma}+\lambda I)^{-1}$ (where $\hat{\Sigma}$ is a sample covariance) behaves similarly to a deterministic matrix of the form $(\hat{\Sigma}+\kappa I)^{-1}$, where $\kappa = \kappa(\lambda, M, \Sigma)$ is an *effective regularizer.*

The effective regularizer $\kappa(\lambda, M, \Sigma)$ is defined as follows.

Definition 1 (Effective regularizer)**.** *For $\lambda \geq 0$, $M \geq 1$, and a P-dimensional positive semidefinite matrix Σ with eigenvalues λ_i for $1 \leq i \leq P$, the value $\kappa(\lambda, M, \Sigma)$ is the unique value $\kappa \geq 0$ such that:*

$$\frac{\lambda}{\kappa} + \frac{1}{N}\sum_{i=1}^{P}\frac{\lambda_i}{\lambda_i+\kappa} = 1.$$

We are now ready to state the key random matrix theory results proven in (3). Following (3), the asymptotic equivalence notation $u \sim v$ means that u/v tends to 1 as N and P go to ∞.

Lemma 36 (Restatement of Proposition 1 in (3))**.** *Assume that $\mathcal{D}_F$ satisfies the Marčenko-Pastur property (Assumption 1). Let $\hat{\Sigma} = \frac{1}{M}\sum_{i=1}^{M} X_i X_i^T$ be the sample covariance matrix from M i.i.d. samples from $X_1, \ldots, X_M \sim \mathcal{D}_F$. Let $\kappa = \kappa(\lambda, N, \Sigma)$. Suppose that A and B have bounded operator norm. Then it holds that:*

$$\begin{aligned}\lambda\,\mathrm{Tr}\left((\hat{\Sigma}+\lambda I)^{-1}A\right) &\sim \kappa\,\mathrm{Tr}\left((\Sigma+\kappa I)^{-1}A\right) \\ \lambda^2\,\mathrm{Tr}\left((\hat{\Sigma}+\lambda I)^{-1}A(\hat{\Sigma}+\lambda I)^{-1}B\right) &\sim \kappa^2\,\mathrm{Tr}\left((\Sigma+\kappa I)^{-1}A(\Sigma+\kappa I)^{-1}B\right) \\ &\quad + \kappa^2\frac{\frac{1}{N}\mathrm{Tr}\left(A\Sigma(\Sigma+\kappa I)^{-2}\right)}{1-\frac{1}{N}\mathrm{Tr}\left(\Sigma^2(\Sigma+\kappa I)^{-2}\right)}\mathrm{Tr}\left((\Sigma+\kappa I)^{-1}\Sigma(\Sigma+\kappa I)^{-1}B\right).\end{aligned}$$

We note that the requirement that B has bounded operator norm in Lemma 36 is what forces us to require that $\|\beta_1\|$ and $\|\beta_2\|$ are bounded. However, (2) showed that the norm can be unbounded in several real-world settings, and thus instead opt to assume a local Marčenko-Pastur law and derive scaling laws based on this assumption. We suspect it may be possible to derive our scaling law with an appropriate analogue of the local Marčenko-Pastur law, which would also have the added benefit of allowing one to relax other requirements in Assumption 1such as gaussianity. We view such an extension as an interesting direction for future work.

5.B. Useful random matrix theory facts. We derive several corollaries of Lemma 36 tailored to random matrices that arise in our analysis of multi-objective scaling laws.

Lemma 37. *Assume that $\mathcal{D}_F$ satisfies the Marčenko-Pastur property (Assumption 1). Let Z be a positive definite matrix such that Z^{-1} has bounded operator norm, and let A be a matrix with bounded operator norm. Let $\hat{\Sigma} = \frac{1}{M}\sum_{i=1}^{M} X_i X_i^T$ be the sample covariance matrix from M i.i.d. samples from $X_1, \ldots, X_M \sim \mathcal{D}_F$. Then it holds that:*

$$\lambda \cdot \mathrm{Tr}((\hat{\Sigma}+\lambda Z)^{-1}A) \sim \kappa \cdot \mathrm{Tr}((\Sigma+\kappa Z)^{-1}A). \qquad [11]$$

If A also has bounded trace and Z has bounded operator norm, then it holds that:

$$\mathrm{Tr}(\hat{\Sigma}(\hat{\Sigma}+\lambda Z)^{-1}A) \sim \mathrm{Tr}(\Sigma \cdot (\Sigma+\kappa Z)^{-1}A) \qquad [12]$$

where $\kappa = \kappa(\lambda, M, Z^{-1/2}\Sigma Z^{-1/2})$.

Proof. For Eq. (11), observe that:

$$\begin{aligned}\lambda \cdot \mathrm{Tr}((\hat{\Sigma}+\lambda Z)^{-1}A) &= \lambda \cdot \mathrm{Tr}(Z^{-1/2}(Z^{-1/2}\hat{\Sigma}Z^{-1/2}+\lambda I)^{-1}Z^{-1/2}A) \\ &= \lambda \cdot \mathrm{Tr}((Z^{-1/2}\hat{\Sigma}Z^{-1/2}+\lambda I)^{-1}Z^{-1/2}AZ^{-1/2}) \\ &\sim_{(A)} \kappa \cdot \mathrm{Tr}((Z^{-1/2}\Sigma Z^{-1/2}+\kappa I)^{-1}Z^{-1/2}AZ^{-1/2}) \\ &= \kappa \cdot \mathrm{Tr}(Z^{-1/2}(Z^{-1/2}\Sigma Z^{-1/2}+\kappa I)^{-1}Z^{-1/2}A) \\ &= \kappa \cdot \mathrm{Tr}((\Sigma+\kappa Z)^{-1}A).\end{aligned}$$

where (A) applies Lemma 36 (using the fact that since A and Z^{-1} have bounded operator norm, it holds that $Z^{-1/2}AZ^{-1/2}$ has bounded operator norm).

For Eq. (12), observe that:

$$\begin{aligned}\mathrm{Tr}(\hat{\Sigma}(\hat{\Sigma}+\lambda Z)^{-1}A) &=_{(A)} \mathrm{Tr}\left(\left(I-\lambda Z^{1/2}\left(Z^{-1/2}\hat{\Sigma}Z^{-1/2}+\lambda I\right)^{-1}Z^{-1/2}\right)A\right) \\ &=_{(B)} \mathrm{Tr}(A) - \lambda \cdot \mathrm{Tr}\left(\left(Z^{-1/2}\hat{\Sigma}Z^{-1/2}+\lambda I\right)^{-1}Z^{-1/2}AZ^{1/2}\right) \\ &\sim_{(C)} \mathrm{Tr}(A) - \kappa \cdot \mathrm{Tr}\left(\left(Z^{-1/2}\Sigma Z^{-1/2}+\kappa I\right)^{-1}Z^{-1/2}AZ^{1/2}\right) \\ &=_{(D)} \mathrm{Tr}\left(\left(I-\kappa Z^{1/2}\left(Z^{-1/2}\Sigma Z^{-1/2}+\kappa I\right)^{-1}Z^{-1/2}\right)A\right) \\ &=_{(E)} \mathrm{Tr}(\Sigma(\Sigma+\kappa Z)^{-1}A)\end{aligned}$$

where (A) and (E) follows from Claim 41, (B) and (D) use the fact that $\mathrm{Tr}(A)$ is bounded, and (C) follows from Lemma 36 (using the fact that since A, Z, and Z^{-1} have bounded operator norm, it holds that $Z^{-1/2}AZ^{1/2}$ has bounded operator norm). □

Lemma 38. *Assume that $\mathcal{D}_F$ satisfies the Marčenko-Pastur property (Assumption 1). Let Z be any positive definite matrix such that Z and Z^{-1} have bounded operator norm, and let A and B have bounded operator norm. Let $\hat{\Sigma}=\frac{1}{M}\sum_{i=1}^{M}X_iX_i^T$ be the sample covariance matrix from M i.i.d. samples from $X_1,\ldots,X_M \sim \mathcal{D}_F$. Then it holds that:*

$$\begin{aligned}&\lambda^2\,\mathrm{Tr}((\hat{\Sigma}+\lambda Z)^{-1}A(\hat{\Sigma}+\lambda Z)^{-1}B) \\ &= \lambda^2\,\mathrm{Tr}(Z^{-1/2}(Z^{-1/2}\hat{\Sigma}Z^{-1/2}+\lambda I)^{-1}Z^{-1/2}AZ^{-1/2}(Z^{-1/2}\hat{\Sigma}Z^{-1/2}+\lambda I)^{-1}B) \\ &\sim \kappa^2\,\mathrm{Tr}((\Sigma+\kappa Z)^{-1}A(\Sigma+\kappa Z)^{-1}B) \\ &+ \kappa^2\frac{\frac{1}{M}\mathrm{Tr}((\Sigma+\kappa Z)^{-1}\Sigma(\Sigma+\kappa Z)^{-1}A)}{1-\frac{1}{M}\mathrm{Tr}((\Sigma+\kappa Z)^{-1}\Sigma(\Sigma+\kappa Z)^{-1}\Sigma)}\,\mathrm{Tr}((\Sigma+\kappa Z)^{-1}\Sigma(\Sigma+\kappa Z)^{-1}B)\end{aligned}$$

where $\kappa=\kappa(\lambda,M,Z^{-1/2}\Sigma Z^{-1/2})$.

Proof. Let $q=\frac{\frac{1}{M}\mathrm{Tr}(Z^{-1/2}\Sigma Z^{-1/2}(Z^{-1/2}\Sigma Z^{-1/2}+\kappa I)^{-2}Z^{-1/2}AZ^{-1/2})}{1-\frac{1}{M}\mathrm{Tr}(Z^{-1/2}\Sigma Z^{-1/2}(Z^{-1/2}\Sigma Z^{-1/2}+\kappa I)^{-2}Z^{-1/2}\Sigma Z^{-1/2})}$.

Observe that:

$$\begin{aligned}&\lambda^2\,\mathrm{Tr}((\hat{\Sigma}+\lambda Z)^{-1}A(\hat{\Sigma}+\lambda Z)^{-1}B) \\ &\lambda^2\,\mathrm{Tr}\left(Z^{-1/2}\left(Z^{-1/2}\hat{\Sigma}Z^{-1/2}+\lambda I\right)^{-1}Z^{-1/2}AZ^{-1/2}\left(Z^{-1/2}\hat{\Sigma}Z^{-1/2}+\lambda I\right)^{-1}Z^{-1/2}B\right) \\ &= \lambda^2\,\mathrm{Tr}\left(\left(Z^{-1/2}\hat{\Sigma}Z^{-1/2}+\lambda I\right)^{-1}Z^{-1/2}AZ^{-1/2}\left(Z^{-1/2}\hat{\Sigma}Z^{-1/2}+\lambda I\right)^{-1}Z^{-1/2}BZ^{-1/2}\right) \\ &\sim_{(A)} \kappa^2\,\mathrm{Tr}\left(\left(Z^{-1/2}\Sigma Z^{-1/2}+\kappa I\right)^{-1}Z^{-1/2}AZ^{-1/2}\left(Z^{-1/2}\Sigma Z^{-1/2}+\kappa I\right)^{-1}Z^{-1/2}BZ^{-1/2}\right) \\ &+ \kappa^2 q\,\mathrm{Tr}\left(\left(Z^{-1/2}\Sigma Z^{-1/2}+\kappa I\right)^{-1}Z^{-1/2}\Sigma Z^{-1/2}\left(Z^{-1/2}\Sigma Z^{-1/2}+\kappa I\right)^{-1}Z^{-1/2}BZ^{-1/2}\right) \\ &= \kappa^2\,\mathrm{Tr}\left(Z^{-1/2}\left(Z^{-1/2}\Sigma Z^{-1/2}+\kappa I\right)^{-1}Z^{-1/2}AZ^{-1/2}\left(Z^{-1/2}\Sigma Z^{-1/2}+\kappa I\right)^{-1}Z^{-1/2}B\right) \\ &+ \kappa^2 q\,\mathrm{Tr}\left(Z^{-1/2}\left(Z^{-1/2}\Sigma Z^{-1/2}+\kappa I\right)^{-1}Z^{-1/2}\Sigma Z^{-1/2}\left(Z^{-1/2}\Sigma Z^{-1/2}+\kappa I\right)^{-1}Z^{-1/2}B\right) \\ &= \kappa^2\,\mathrm{Tr}\left((\Sigma+\kappa Z)^{-1}A(\Sigma+\kappa Z)^{-1}B\right)+q\kappa^2\,\mathrm{Tr}\left((\Sigma+\kappa Z)^{-1}\Sigma(\Sigma+\kappa Z)^{-1}B\right),\end{aligned}$$

where (A) follows from Lemma 36 (using the fact that since A, B, Z, and Z^{-1} have bounded operator norm, it holds that $Z^{-1/2}AZ^{1/2}$, Σ, and $Z^{-1/2}BZ^{1/2}$ have bounded operator norm).

We can simplify q as follows:

$$
\begin{aligned}
q &= \frac{\frac{1}{M}\operatorname{Tr}(Z^{-1/2}\Sigma Z^{-1/2}(Z^{-1/2}\Sigma Z^{-1/2}+\kappa I)^{-2}Z^{-1/2}AZ^{-1/2})}{1-\frac{1}{M}\operatorname{Tr}(Z^{-1/2}\Sigma Z^{-1/2}(Z^{-1/2}\Sigma Z^{-1/2}+\kappa I)^{-2}Z^{-1/2}\Sigma Z^{-1/2})} \\
&= \frac{\frac{1}{M}\operatorname{Tr}((Z^{-1/2}\Sigma Z^{-1/2}+\kappa I)^{-1}Z^{-1/2}\Sigma Z^{-1/2}(Z^{-1/2}\Sigma Z^{-1/2}+\kappa I)^{-1}Z^{-1/2}AZ^{-1/2})}{1-\frac{1}{M}\operatorname{Tr}((Z^{-1/2}\Sigma Z^{-1/2}+\kappa I)^{-1}Z^{-1/2}\Sigma Z^{-1/2}(Z^{-1/2}\Sigma Z^{-1/2}+\kappa I)^{-1}Z^{-1/2}\Sigma Z^{-1/2})} \\
&= \frac{\frac{1}{M}\operatorname{Tr}(Z^{-1/2}(Z^{-1/2}\Sigma Z^{-1/2}+\kappa I)^{-1}Z^{-1/2}\Sigma Z^{-1/2}(Z^{-1/2}\Sigma Z^{-1/2}+\kappa I)^{-1}Z^{-1/2}A)}{1-\frac{1}{M}\operatorname{Tr}(Z^{-1/2}(Z^{-1/2}\Sigma Z^{-1/2}+\kappa I)^{-1}Z^{-1/2}\Sigma Z^{-1/2}(Z^{-1/2}\Sigma Z^{-1/2}+\kappa I)^{-1}Z^{-1/2}\Sigma)} \\
&= \frac{\frac{1}{M}\operatorname{Tr}((\Sigma+\kappa Z)^{-1}\Sigma(\Sigma+\kappa Z)^{-1}A)}{1-\frac{1}{M}\operatorname{Tr}((\Sigma+\kappa Z)^{-1}\Sigma(\Sigma+\kappa Z)^{-1}\Sigma)}.
\end{aligned}
$$

□

Lemma 39. *Assume that $\mathcal{D}_F$ satisfies the Marčenko-Pastur property (Assumption 1). Let Z be any positive definite matrix such that Z and Z^{-1} have bounded operator norm. Let A and B have bounded operator norm, and suppose also that $\operatorname{Tr}(AB)$ is bounded. Let $\hat{\Sigma} = \frac{1}{M}\sum_{i=1}^{M} X_i X_i^T$ be the sample covariance matrix from M i.i.d. samples from $X_1, \ldots, X_M \sim \mathcal{D}_F$. Then it holds that:*

$$\operatorname{Tr}(\hat{\Sigma}(\hat{\Sigma}+\lambda Z)^{-1}A(\hat{\Sigma}+\lambda Z)^{-1}\hat{\Sigma}B) \sim \operatorname{Tr}(\Sigma(\Sigma+\kappa Z)^{-1}A(\Sigma+\kappa Z)^{-1}\Sigma B) + E, \tag*{[13]}$$

where:

$$E := \frac{\frac{1}{M}\operatorname{Tr}((\Sigma+\kappa Z)^{-1}\Sigma(\Sigma+\kappa Z)^{-1}A)}{1-\frac{1}{M}\operatorname{Tr}((\Sigma+\kappa Z)^{-1}\Sigma(\Sigma+\kappa Z)^{-1}\Sigma)} \cdot \kappa^2 \operatorname{Tr}\left((\Sigma+\kappa Z)^{-1}\Sigma(\Sigma+\kappa Z)^{-1}ZBZ\right),$$

and $\kappa = \kappa(\lambda, M, Z^{-1/2}\Sigma Z^{-1/2})$.

Proof. Observe that:

$$
\begin{aligned}
&\operatorname{Tr}(\hat{\Sigma}(\hat{\Sigma}+\lambda Z)^{-1}A(\hat{\Sigma}+\lambda Z)^{-1}\hat{\Sigma}B) \\
&= \operatorname{Tr}(\hat{\Sigma}(\hat{\Sigma}+\lambda Z)^{-1}A\left(\hat{\Sigma}(\hat{\Sigma}+\lambda Z)^{-1}\right)^T B) \\
&=_{(A)} \operatorname{Tr}\left(\left(I-\lambda Z^{1/2}\left(Z^{-1/2}\hat{\Sigma}Z^{-1/2}+\lambda I\right)^{-1}Z^{-1/2}\right)A\left(I-\lambda Z^{1/2}\left(Z^{-1/2}\hat{\Sigma}Z^{-1/2}+\lambda I\right)^{-1}Z^{-1/2}\right)^T B\right) \\
&=_{(B)} \operatorname{Tr}(AB) - \lambda\operatorname{Tr}\left(A\left(Z^{1/2}\left(Z^{-1/2}\hat{\Sigma}Z^{-1/2}+\lambda I\right)^{-1}Z^{-1/2}\right)^T B\right) \\
&- \lambda\operatorname{Tr}\left(Z^{1/2}\left(Z^{-1/2}\hat{\Sigma}Z^{-1/2}+\lambda I\right)^{-1}Z^{-1/2}AB\right) \\
&+ \lambda^2\operatorname{Tr}\left(Z^{1/2}\left(Z^{-1/2}\hat{\Sigma}Z^{-1/2}+\lambda I\right)^{-1}Z^{-1/2}A\left(Z^{1/2}\left(Z^{-1/2}\hat{\Sigma}Z^{-1/2}+\lambda I\right)^{-1}Z^{-1/2}\right)^T B\right) \\
&= \operatorname{Tr}(AB) - \lambda\operatorname{Tr}\left(AZ^{-1/2}\left(Z^{-1/2}\hat{\Sigma}Z^{-1/2}+\lambda I\right)^{-1}Z^{1/2}B\right) \\
&- \lambda\operatorname{Tr}\left(Z^{1/2}\left(Z^{-1/2}\hat{\Sigma}Z^{-1/2}+\lambda I\right)^{-1}Z^{-1/2}AB\right) \\
&+ \lambda^2\operatorname{Tr}\left(Z^{1/2}\left(Z^{-1/2}\hat{\Sigma}Z^{-1/2}+\lambda I\right)^{-1}Z^{-1/2}AZ^{-1/2}\left(Z^{-1/2}\hat{\Sigma}Z^{-1/2}+\lambda I\right)^{-1}Z^{1/2}B\right) \\
&= \operatorname{Tr}(AB) - \underbrace{\lambda\operatorname{Tr}\left(\left(\hat{\Sigma}+\lambda Z\right)^{-1}ZBA\right)}_{(1)} - \underbrace{\lambda\operatorname{Tr}\left(\left(\hat{\Sigma}+\lambda Z\right)^{-1}ABZ\right)}_{(2)} \\
&+ \underbrace{\lambda^2\operatorname{Tr}\left(\left(\hat{\Sigma}+\lambda Z\right)^{-1}A\left(\hat{\Sigma}+\lambda Z\right)^{-1}ZBZ\right)}_{(3)}
\end{aligned}
$$

where (A) follows from Claim 41, (B) uses that $\operatorname{Tr}(AB)$ is bounded,

For term (1) and term (2), we apply Lemma 37 to see that:

$$
\begin{aligned}
\lambda\operatorname{Tr}\left(\left(\hat{\Sigma}+\lambda Z\right)^{-1}ZBA\right) &\sim \kappa\lambda\operatorname{Tr}\left((\Sigma+\kappa Z)^{-1}ZBA\right) \\
\lambda\operatorname{Tr}\left(\left(\hat{\Sigma}+\lambda Z\right)^{-1}ABZ\right) &\sim \kappa\operatorname{Tr}\left((\Sigma+\kappa Z)^{-1}ABZ\right).
\end{aligned}
$$

For term (3), we apply Lemma 38 to see that

$$\begin{aligned}
&\lambda^2 \operatorname{Tr}\left(\left(\hat{\Sigma}+\lambda Z\right)^{-1} A \left(\hat{\Sigma}+\lambda Z\right)^{-1} ZBZ\right) \\
&\sim \kappa^2 \operatorname{Tr}\left((\Sigma+\kappa Z)^{-1} A \left(\Sigma+\kappa Z\right)^{-1} ZBZ\right) \\
&+ \kappa^2 \frac{\frac{1}{M}\operatorname{Tr}((\Sigma+\kappa Z)^{-1}\Sigma(\Sigma+\kappa Z)^{-1}A)}{1-\frac{1}{M}\operatorname{Tr}((\Sigma+\kappa Z)^{-1}\Sigma(\Sigma+\kappa Z)^{-1}\Sigma)} \operatorname{Tr}\left((\Sigma+\kappa Z)^{-1}\Sigma(\Sigma+\kappa Z)^{-1}ZBZ\right) \\
&\sim \kappa^2 \operatorname{Tr}\left((\Sigma+\kappa Z)^{-1} A \left(\Sigma+\kappa Z\right)^{-1} ZBZ\right) + E
\end{aligned}$$

This means that:

$$\begin{aligned}
\operatorname{Tr}\left(\hat{\Sigma}(\hat{\Sigma}+\lambda Z)^{-1}A(\hat{\Sigma}+\lambda Z)^{-1}\hat{\Sigma}\right) &\sim \operatorname{Tr}(AB) - \kappa \operatorname{Tr}\left((\Sigma+\kappa Z)^{-1} ZBA\right) - \kappa \operatorname{Tr}\left((\Sigma+\kappa Z)^{-1} ABZ\right) \\
&+ \kappa^2 \operatorname{Tr}\left((\Sigma+\kappa Z)^{-1} A \left(\Sigma+\kappa Z\right)^{-1} ZBZ\right) + E \\
&=_{(C)} \operatorname{Tr}(\Sigma(\Sigma+\kappa Z)^{-1}A(\Sigma+\kappa Z)^{-1}\Sigma B) + E,
\end{aligned}$$

where (C) uses an analogous analysis to the beginning of the proof. □

Lemma 40. *Assume that $\mathcal{D}_F$ satisfies the Marčenko-Pastur property (Assumption 1). Let Z be any positive definite matrix such that Z and Z^{-1} have bounded operator norm, and let A and B have bounded operator norm. Let $\hat{\Sigma} = \frac{1}{M}\sum_{i=1}^{M} X_i X_i^T$ be the sample covariance matrix from M i.i.d. samples from $X_1, \ldots, X_M \sim \mathcal{D}_F$. Then it holds that:*

$$\lambda \operatorname{Tr}\left((\hat{\Sigma}+\lambda Z)^{-1}A(\hat{\Sigma}+\lambda Z)^{-1}\hat{\Sigma}B\right) \sim \kappa \operatorname{Tr}\left((\Sigma+\kappa Z)^{-1}A(\Sigma+\kappa Z)^{-1}\Sigma B\right) - E, \qquad [14]$$

where:

$$E := \frac{\frac{1}{M}\operatorname{Tr}((\Sigma+\kappa Z)^{-1}\Sigma(\Sigma+\kappa Z)^{-1}A)}{1-\frac{1}{M}\operatorname{Tr}((\Sigma+\kappa Z)^{-1}\Sigma(\Sigma+\kappa Z)^{-1}\Sigma)} \cdot \kappa^2 \operatorname{Tr}\left((\Sigma+\kappa Z)^{-1}\Sigma\left(\Sigma+\kappa Z\right)^{-1} ZB\right)$$

and $\kappa = \kappa(\lambda, N, Z^{-1/2}\Sigma Z^{-1/2})$.

Proof. Observe that:

$$\begin{aligned}
&\lambda \operatorname{Tr}\left((\hat{\Sigma}+\lambda Z)^{-1}A(\hat{\Sigma}+\lambda Z)^{-1}\hat{\Sigma}B\right) \\
&=_{(A)} \lambda \operatorname{Tr}\left(Z^{-1/2}(Z^{-1/2}\hat{\Sigma}Z^{-1/2}+\lambda I)^{-1}Z^{-1/2}A\left(I-\lambda Z^{-1/2}\left(Z^{-1/2}\hat{\Sigma}Z^{-1/2}+\lambda I\right)^{-1}Z^{1/2}\right)B\right) \\
&= \lambda \operatorname{Tr}\left(Z^{-1/2}(Z^{-1/2}\hat{\Sigma}Z^{-1/2}+\lambda I)^{-1}Z^{-1/2}AB\right) \\
&- \lambda^2 \operatorname{Tr}\left(Z^{-1/2}\left(Z^{-1/2}\hat{\Sigma}Z^{-1/2}+\lambda I\right)^{-1}Z^{-1/2}AZ^{-1/2}(Z^{-1/2}\hat{\Sigma}Z^{-1/2}+\lambda I)^{-1}Z^{1/2}B\right) \\
&= \underbrace{\lambda \operatorname{Tr}\left((\hat{\Sigma}+\lambda Z)^{-1}AB\right)}_{(1)} - \underbrace{\lambda^2 \operatorname{Tr}\left(\left(\hat{\Sigma}+\lambda Z\right)^{-1}A(\hat{\Sigma}+\lambda Z)^{-1}ZB\right)}_{(2)}
\end{aligned}$$

where (A) follows from Claim 41.

For term (1), we apply Lemma 37 see that:

$$\lambda \operatorname{Tr}\left((\hat{\Sigma}+\lambda Z)^{-1}AB\right) \sim \kappa \operatorname{Tr}\left((\Sigma+\kappa Z)^{-1}AB\right).$$

For term (2), we apply Lemma 38 to see that

$$\begin{aligned}
&\lambda^2 \operatorname{Tr}\left(\left(\hat{\Sigma}+\lambda Z\right)^{-1}A(\hat{\Sigma}+\lambda Z)^{-1}ZB\right) \\
&\sim \kappa^2 \operatorname{Tr}\left((\Sigma+\kappa Z)^{-1}A\left(\Sigma+\kappa Z\right)^{-1}ZB\right) \\
&+ \kappa^2 \frac{\frac{1}{M}\operatorname{Tr}((\Sigma+\kappa Z)^{-1}\Sigma(\Sigma+\kappa Z)^{-1}A)}{1-\frac{1}{M}\operatorname{Tr}((\Sigma+\kappa Z)^{-1}\Sigma(\Sigma+\kappa Z)^{-1}\Sigma)} \operatorname{Tr}\left((\Sigma+\kappa Z)^{-1}\Sigma(\Sigma+\kappa Z)^{-1}ZB\right) \\
&\sim \kappa^2 \operatorname{Tr}\left((\Sigma+\kappa Z)^{-1}A\left(\Sigma+\kappa Z\right)^{-1}ZB\right) + E.
\end{aligned}$$

This means that:

$$\begin{aligned}
&\lambda \operatorname{Tr}\left((\hat{\Sigma}+\lambda Z)^{-1}A(\hat{\Sigma}+\lambda Z)^{-1}\hat{\Sigma}B\right) \\
&\sim \kappa \operatorname{Tr}\left((\Sigma+\kappa Z)^{-1}AB\right) + \kappa^2 \operatorname{Tr}\left((\Sigma+\kappa Z)^{-1}A\left(\Sigma+\kappa Z\right)^{-1}ZB\right) - E \\
&= \kappa \operatorname{Tr}\left(Z^{-1/2}(Z^{-1/2}\Sigma Z^{-1/2}+\kappa I)^{-1}Z^{-1/2}A\left(I-\kappa Z^{1/2}\left(Z^{-1/2}\Sigma Z^{-1/2}+\kappa I\right)^{-1}Z^{-1/2}\right)B\right) - E \\
&=_{(A)} \kappa \operatorname{Tr}\left((\Sigma+\kappa Z)^{-1}A(\Sigma+\kappa Z)^{-1}\Sigma B\right) - E,
\end{aligned}$$

where (A) uses an analogous analysis to the beginning of the proof. □

The proofs of these results relied on the following basic matrix fact.

Claim 41. *Let A be any matrix and let B be any symmetric positive definite matrix. Then it holds that:*

$$A(A+\lambda B)^{-1} = I - \lambda B^{1/2}\left(B^{-1/2}AB^{-1/2}+\lambda I\right)^{-1}B^{-1/2}.$$

Proof. Observe that:

$$\begin{aligned}
&A(A+\lambda B)^{-1}\\
&= AB^{-1/2}\left(B^{-1/2}AB^{-1/2}+\lambda I\right)^{-1}B^{-1/2}\\
&= B^{1/2}\left(B^{-1/2}AB^{-1/2}\right)\left(B^{-1/2}AB^{-1/2}+\lambda I\right)^{-1}B^{-1/2}\\
&= B^{1/2}\left(B^{-1/2}AB^{-1/2}+\lambda I\right)\left(B^{-1/2}AB^{-1/2}+\lambda I\right)^{-1}B^{-1/2} - B^{1/2}\lambda\left(B^{-1/2}AB^{-1/2}+\lambda I\right)^{-1}B^{-1/2}\\
&= I - \lambda B^{1/2}\left(B^{-1/2}AB^{-1/2}+\lambda I\right)^{-1}B^{-1/2}.
\end{aligned}$$

□

6. Extension: Market-entry threshold with richer form for L_2^*

In this section, we modify the safety requirement to take into account the impact of dataset size N and regularization parameter λ, and we extend our model and analysis of the market-entry threshold accordingly. We show that the characterization in Theorem 1directly applies to this setting, and we also show relaxed versions of Theorem 4and Theorem 5. Altogether, these extended results illustrate that our qualitative insights from the main body hold more generally.

We define a modified approximation of the safety violation $\tilde{L}_2(\beta_1, \beta_2, \mathcal{D}_F, \lambda, N, \alpha)$. This modified approximation is defined analogously to $L_1^*(\beta_1, \beta_2, \mathcal{D}_F, \lambda, N, \alpha)$. To formalize this, we define a deterministic equivalent L_2^{det} for the safety violation to be

$$L_2^{\text{det}}(\beta_1, \beta_2, \mathcal{D}_F, \lambda, N, \alpha) := L_1^{\text{det}}(\beta_2, \beta_1, \mathcal{D}_F, \lambda, N, 1-\alpha). \quad [15]$$

It follows from Lemma 6that $L_2(\hat{\beta}(\alpha, \lambda, X)) \sim L_2^{\text{det}}(\beta_1, \beta_2, \mathcal{D}_F, \lambda, N, \alpha)$: here, we use the fact that $L_2(\hat{\beta}(\alpha, \lambda, X))$ is distributed identically to $L_1(\hat{\beta}(1-\alpha, \lambda, X))$. Now, using this deterministic equivalent, we define $\tilde{L}_2(\beta_1, \beta_2, \mathcal{D}_F, \lambda, N, \alpha) = L_2^{\text{det}}(\beta_1, \beta_2, \mathcal{D}_F, \lambda, N, \alpha)$.

Using this formulation of $\tilde{L}_2$, we define a modified market entry threshold where we replace all instances of original approximation L_2^* with the modified approximation $\tilde{L}_2$. In particular, a company C faces reputational damage if:

$$\mathbb{E}_{(\beta_1,\beta_2)\sim\mathcal{D}_W}\tilde{L}_2(\beta_1, \beta_2, \mathcal{D}_F, \alpha_C) \geq \tau_C.$$

The company selects $\alpha \in [0.5, 1]$ and $\lambda \in (0, 1)$ to maximize their performance subject to their safety constraint, as formalized by the following optimization program:[‡]

$$(\tilde{\alpha}_C, \tilde{\lambda}_C) = \text{argmin}_{\alpha\in[0.5,1],\lambda\in(0,1)}\,\mathbb{E}_{\mathcal{D}_W}[L_1^*(\beta_1, \beta_2, \mathcal{D}_F, \lambda, N_C, \alpha)] \text{ s.t. } \mathbb{E}_{\mathcal{D}_W}[\tilde{L}_2(\beta_1, \beta_2, \mathcal{D}_F, \alpha)] \leq \tau_C.$$

We define the modified market-entry threshold as follows.

Definition 2. *The modified market-entry threshold $\tilde{N}_E^*(N_I, \tau_I, \tau_E, \mathcal{D}_W, \mathcal{D}_F)$ is the minimum value of $N_E \in \mathbb{Z}_{\geq 1}$ such that $\mathbb{E}_{\mathcal{D}_W}[L_1^*(\beta_1, \beta_2, \mathcal{D}_F, \tilde{\lambda}_E, N_E, \tilde{\alpha}_E)] \leq \mathbb{E}_{\mathcal{D}_W}[L_1^*(\beta_1, \beta_2, \mathcal{D}_F, \tilde{\lambda}_I, N_I, \tilde{\alpha}_I)]$.*

In this section, we analyze the modified market entry threshold $\tilde{N}_E^*(N_I, \tau_I, \tau_E, \mathcal{D}_W, \mathcal{D}_F)$. We show an extension of Theorem 1(Appendix 6.A). We then derive a simplified version of the deterministic equivalent L_2^{det} (Appendix 6.B). Finally, we show a weakened extension of Theorem 4(Appendix 6.C) and a weakened extension of Theorem 5(Appendix 6.D). These weakened extensions derive upper bounds (rather than tight bounds) on the modified market entry threshold, and also assume that $\delta \leq 1$.

6.A. Extension of Theorem 1. We study the market entry $\tilde{N}_E^*$ threshold in the environment of Theorem 1where the incumbent has infinite data and the new company faces no safety constraint. We show that the modified market entry threshold takes the same form as the market entry threshold in Theorem 1.

Theorem 42 (Extension of Theorem 1)**.** *Suppose that the power-law scaling assumptions hold with exponents $\gamma, \delta > 0$ and correlation coefficient $\rho \in [0, 1)$, and suppose that $P = \infty$. Suppose that the incumbent company has infinite data (i.e., $N_I = \infty$), and that the entrant faces no constraint on their safety (i.e., $\tau_E = \infty$). Suppose that the safety constraint τ_I satisfies (1). Then, it holds that:*

$$\tilde{N}_E^*(\infty, \tau_I, \infty, \mathcal{D}_W, \mathcal{D}_F) = \Theta\left(\left(\sqrt{L^*(\rho)} - \sqrt{\min(\tau_I, L^*(\rho))}\right)^{-2/\nu}\right),$$

where $L^*(\rho) = \mathbb{E}_{\mathcal{D}_W}[(\beta_1-\beta_2)^T\Sigma(\beta_1-\beta_2)] = \Theta(1-\rho)$, and where $\nu := \min(2(1+\gamma), \delta+\gamma)$.

‡ Here, there might not exist $\alpha \in [0.5, 1]$ and $\lambda \in (0, 1)$ which satisfy the safety constraint, if N_C is too small.

Theorem 42 shows that the qualitative insights from Theorem 1—including that the new company can enter with finite data—readily extend to this setting.

To prove Theorem 42, we build on the notation and analysis from Appendix 2. It suffices to show that each company C will select $\alpha_C = \tilde{\alpha}_C$ and $\lambda_C = \tilde{\lambda}_C$. This follows trivially for the entrant $C = E$ since they face no safety constraint, and there is no different between the two settings. The key ingredient of the proof is to compute $\tilde{\alpha}_I$ and $\tilde{\lambda}_I$ for the incumbent (i.e., an analogue of Lemma 12 in Appendix 2).

To do this, we first upper bound the following function of the safety loss and performance loss for general parameters λ and α.

Lemma 43. *For any α and λ, it holds that:*

$$\sqrt{\mathbb{E}_{\mathcal{D}_W}[L_1(\beta(\alpha,\lambda))]} + \sqrt{\mathbb{E}_{\mathcal{D}_W}[L_2(\beta(\alpha,\lambda))]} \geq \sqrt{\mathbb{E}_{\mathcal{D}_W}[(\beta_1-\beta_2)^T\Sigma(\beta_1-\beta_2)^T]}.$$

Proof. Note that:

$$\begin{aligned}
T &:= \sqrt{\mathbb{E}_{\mathcal{D}_W}[L_1(\beta(\alpha,\lambda))]} + \sqrt{\mathbb{E}_{\mathcal{D}_W}[L_2(\beta(\alpha,\lambda))]} \\
&= \sqrt{(\beta_1-\beta(\alpha,\lambda))^T\Sigma(\beta_1-\beta(\alpha,\lambda))} + \sqrt{(\beta_2-\beta(\alpha,\lambda))^T\Sigma(\beta_2-\beta(\alpha,\lambda))} \\
&= \sqrt{(\lambda\beta_1+(1-\alpha)\Sigma(\beta_1-\beta_2))^T\Sigma(\Sigma+\lambda I)^{-2}(\lambda\beta_1+(1-\alpha)\Sigma(\beta_1-\beta_2))} \\
&+ \sqrt{(\lambda\beta_2+\alpha\Sigma(\beta_2-\beta_1))^T\Sigma(\Sigma+\lambda I)^{-2}(\lambda\beta_2+\alpha\Sigma(\beta_2-\beta_1))} \\
&= \sqrt{(\lambda\beta_1+(1-\alpha)\Sigma(\beta_1-\beta_2))^T\Sigma(\Sigma+\lambda I)^{-2}(\lambda\beta_1+(1-\alpha)\Sigma(\beta_1-\beta_2))} \\
&+ \sqrt{(-\lambda\beta_2+\alpha\Sigma(\beta_1-\beta_2))^T\Sigma(\Sigma+\lambda I)^{-2}(-\lambda\beta_2+\alpha\Sigma(\beta_1-\beta_2))}.
\end{aligned}$$

Now note that for any PSD matrix Σ' and any distribution, note that the following triangle inequality holds:

$$\sqrt{\mathbb{E}[(X_1+X_2)^T\Sigma'(X_1+X_2)]} \leq \sqrt{\mathbb{E}[X_1^T\Sigma' X_1]} + \sqrt{\mathbb{E}[X_2^T\Sigma' X_2]}.$$

We apply this for $X_1 = \lambda\beta_1+(1-\alpha)\Sigma(\beta_1-\beta_2)$, $X_2 = -\lambda\beta_2+\alpha\Sigma(\beta_1-\beta_2)$, and distribution $\mathcal{D}_W$. This means that we can lower bound:

$$\begin{aligned}
T &\geq \sqrt{\mathbb{E}_{\mathcal{D}_W}[((\Sigma+\lambda I)(\beta_1-\beta_2))^T\Sigma(\Sigma+\lambda I)^{-2}((\Sigma+\lambda I)(\beta_1-\beta_2))]} \\
&= \sqrt{\mathbb{E}_{\mathcal{D}_W}[(\beta_1-\beta_2))^T\Sigma(\beta_1-\beta_2))]}
\end{aligned}$$

as desired. ☐

Now, we are ready to compute $\tilde{\alpha}_I$ and $\tilde{\lambda}_I$ for the incumbent.

Lemma 44. *Let $L^*(\rho) = \mathbb{E}_{\mathcal{D}_W}[(\beta_1-\beta_2)^T\Sigma(\beta_1-\beta_2)^T]$. Suppose that $N_I = \infty$, and suppose that the safety constraint τ_I satisfies (1). Then it holds that $\alpha_I = \sqrt{\frac{\min(\tau_I, L^*(\rho))}{L^*(\rho)}}$, and $\lambda_I = 0$ is optimal for the incumbent. Moreover, it holds that:*

$$\mathbb{E}_{\mathcal{D}_W}[L_1^*(\beta_1,\beta_2,\mathcal{D}_F,\tilde{\lambda}_I,\infty,\tilde{\alpha}_I)] = \left(\sqrt{L^*(\rho)} - \sqrt{\min(L^*(\rho),\tau_I)}\right)^2.$$

Proof. First, we apply Lemma 46 with $N = \infty$ to see that:

$$\mathbb{E}_{\mathcal{D}_W}[L_1^*(\beta_1,\beta_2,\mathcal{D}_F,\lambda,\infty,\alpha)] = \mathbb{E}_{\mathcal{D}_W}[L_1(\beta(\alpha,\lambda))]$$

and

$$\mathbb{E}_{\mathcal{D}_W}[L_2^*(\beta_1,\beta_2,\mathcal{D}_F,\lambda,\infty,\alpha)] = \mathbb{E}_{\mathcal{D}_W}[L_2(\beta(\alpha,\lambda))].$$

Let $\alpha^* = \sqrt{\frac{\min(\tau_I, L^*(\rho))}{L^*(\rho)}}$. By the assumption in the lemma statement, we know that:

$$\alpha^* \geq \sqrt{\frac{\mathbb{E}_{\mathcal{D}_W}[L_2^*(\beta_1,\beta_2,\mathcal{D}_F,0.5)]}{L^*(\rho)}} = 0.5.$$

Observe that:

$$\begin{aligned}
&\sqrt{\mathbb{E}_{\mathcal{D}_W}[L_1(\beta(\alpha^*,0))]} + \sqrt{\min(\tau_I, L^*(\rho))} \\
&= \sqrt{\mathbb{E}_{\mathcal{D}_W}[L_1(\beta(\alpha^*,0))]} + \sqrt{\mathbb{E}_{\mathcal{D}_W}[L_2(\beta(\alpha^*,0))]} \\
&= \sqrt{(1-\alpha^*)^2\mathbb{E}_{\mathcal{D}_W}[(\beta_1-\beta_2)^T\Sigma(\beta_1-\beta_2)^T]} + \sqrt{(\alpha^*)^2\mathbb{E}_{\mathcal{D}_W}[(\beta_1-\beta_2)^T\Sigma(\beta_1-\beta_2)^T]} \\
&= \sqrt{\mathbb{E}_{\mathcal{D}_W}[(\beta_1-\beta_2)^T\Sigma(\beta_1-\beta_2)^T]}
\end{aligned}$$

We show that $(\tilde{\alpha}_I, \tilde{\lambda}_I) = (\alpha^*, 0)$. Assume for sake of contradiction that $(\alpha, \lambda) \neq (\alpha^*, 0)$ satisfies the safety constraint $\mathbb{E}_{\mathcal{D}_W}[\tilde{L}_2(\beta_1, \beta_2, \mathcal{D}_F, \alpha)] \leq \min(\tau_I, L^*(\rho))$ and achieves strictly better performance loss:

$$\mathbb{E}_{\mathcal{D}_W}[L_1^*(\beta_1, \beta_2, \mathcal{D}_F, \lambda, \infty, \alpha)] < \mathbb{E}_{\mathcal{D}_W}[L_1^*(\beta_1, \beta_2, \mathcal{D}_F, 0, \infty, \alpha^*)].$$

Then it would hold that:

$$\begin{aligned}\sqrt{\mathbb{E}_{\mathcal{D}_W}[L_1(\beta(\alpha, \lambda))]} + \sqrt{\mathbb{E}_{\mathcal{D}_W}[L_2(\beta(\alpha, \lambda))]} &< \sqrt{\mathbb{E}_{\mathcal{D}_W}[L_1(\beta(\alpha^*, 0))]} + \sqrt{\min(\tau_I, L^*(\rho))} \\ &= \sqrt{\mathbb{E}_{\mathcal{D}_W}[(\beta_1 - \beta_2)^T \Sigma (\beta_1 - \beta_2)^T]},\end{aligned}$$

which contradicts Lemma 43.

To analyze the loss, note that:

$$\begin{aligned}&\mathbb{E}_{\mathcal{D}_W}[L_1^*(\beta_1, \beta_2, \mathcal{D}_F, \tilde{\lambda}_I, \infty, \tilde{\alpha}_I)] \\ &\qquad = \mathbb{E}_{\mathcal{D}_W}[L_1(\beta(\tilde{\alpha}_I, \tilde{\lambda}_I))] \\ &\qquad = (1 - \tilde{\alpha}_I)^2 L^*(\rho) \\ &\qquad = (\sqrt{L^*(\rho)} - \sqrt{\min(L^*(\rho), \tau_I)})^2\end{aligned}$$

□

We now prove Theorem 42.

Proof of Theorem 42. We analyze $(\tilde{\alpha}_C, \tilde{\lambda}_C)$ first for the incumbent $C = I$ and then for the entrant $C = E$.

Analysis of the incumbent $C = I$. By Lemma 44, we see that:

$$\mathbb{E}_{\mathcal{D}_W}[L_1^*(\beta_1, \beta_2, \mathcal{D}_F, \tilde{\lambda}_I, \infty, \tilde{\alpha}_I)] = \left(\sqrt{L^*(\rho)} - \sqrt{\min(\tau_I, L^*(\rho))}\right)^2.$$

Analysis of the entrant $C = E$. This analysis follows identically to the analogous case in the proof of Theorem 1, and we repeat the proof for completeness. Since the entrant faces no safety constraint, the entrant can choose any $\alpha \in [0.5, 1]$. We apply Corollary 8to see that:

$$\mathbb{E}_{\mathcal{D}_W}[L_1^*(\beta_1, \beta_2, \mathcal{D}_F, \lambda_E, N, \alpha_E)] = \inf_{\alpha \in [0.5,1]} \inf_{\lambda > 0} \mathbb{E}_{\mathcal{D}_W}[L_1^*(\beta_1, \beta_2, \mathcal{D}_F, \lambda, N, \alpha)] = \Theta\left(N^{-\nu}\right),$$

which means that:

$$N_E^*(\infty, \tau_I, \infty, \mathcal{D}_W, \mathcal{D}_F) = \Theta\left(\left(\sqrt{L^*(\rho)} - \sqrt{\min(\tau_I, L^*(\rho))}\right)^{-2/\nu}\right)$$

as desired. We can further apply Claim 13 to see that $L^*(\rho) = \Theta(1 - \rho)$. □

6.B. Bounds on the excess loss for safety. We bound the excess loss $\alpha^2 L^*(\rho) - \mathbb{E}_{\mathcal{D}_W}[L_2^{\text{det}}]$. We assume that $\alpha \geq 0.5$ and we further assume that $\delta \leq 1$.

Lemma 45. *Suppose that power scaling holds for the eigenvalues and alignment coefficients with scaling $\gamma > 0$ and $\delta \in (0, 1]$, and correlation coefficient $\rho \in [0, 1)$, and suppose that $P = \infty$. Suppose that $\alpha \geq 0.5$, $\lambda \in (0, 1)$, and $N \geq 1$. Let $L_2^{det} := L_2^{det}(\beta_1, \beta_2, \mathcal{D}_F, \lambda, N, \alpha)$ be defined according to* Eq. (15)*. Let $L^*(\rho) = \mathbb{E}_{\mathcal{D}_W}[(\beta_1 - \beta_2)^T \Sigma (\beta_1 - \beta_2)]$. Then it holds that:*

$$\alpha^2 L^*(\rho) - \mathbb{E}_{\mathcal{D}_W}[L_2^{det}] = O\left(\max(\lambda^{\frac{\nu}{1+\gamma}}, N^{-\nu})\right)$$

and

$$\mathbb{E}_{\mathcal{D}_W}[L_2^{det}] - \alpha^2 L^*(\rho) = O\left(\max(\lambda^{\frac{\nu}{1+\gamma}}, N^{-\nu}) + (1 - \alpha)(1 - \rho)\frac{\min(\lambda^{-\frac{1}{1+\gamma}}, N)}{N}\right),$$

where $\nu = \min(2(1 + \gamma), \delta + \gamma) = \delta + \gamma$.

To prove Lemma 45, we first simplify the deterministic equivalent $L_2^{\text{det}}(\beta_1, \beta_2, \mathcal{D}_F, \lambda, N, \alpha)$ using the power-law scaling assumptions.

Lemma 46. *Suppose that power scaling holds for the eigenvalues and alignment coefficients with scaling $\gamma, \delta > 0$ and correlation coefficient $\rho \in [0, 1)$, and suppose that $P = \infty$. Suppose that $\lambda \in (0, 1)$, and $N \geq 1$. Let $L_2^{\mathrm{det}} := L_2^{\mathrm{det}}(\beta_1, \beta_2, \mathcal{D}_F, \lambda, N, \alpha)$ be defined according to Eq. (15). Let $\kappa = \kappa(\lambda, N, \Sigma)$ from Definition 1. Let $L^*(\rho) = \mathbb{E}_{\mathcal{D}_W}[(\beta_1 - \beta_2)^T \Sigma (\beta_1 - \beta_2)]$. Then it holds that:*

$$\begin{aligned}\mathbb{E}_{\mathcal{D}_W}[L_2^{\mathrm{det}}] - L^*(\rho) = {} & Q^{-1} \cdot \kappa^2 \sum_{i=1}^{P} \frac{i^{-\delta-1-\gamma}}{(i^{-1-\gamma} + \kappa)^2} + Q^{-1} 2\kappa\alpha(1-\alpha)(1-\rho) \sum_{i=1}^{P} \frac{i^{-\delta-2(1+\gamma)}}{(i^{-1-\gamma}+\kappa)^2} \\ & + Q^{-1} 2\alpha(1-\alpha)(1-\rho) \frac{1}{N} \left(\sum_{i=1}^{P} \frac{i^{-2-2\gamma}}{(i^{-1-\gamma}+\kappa)^2} \right) \cdot \sum_{i=1}^{P} \frac{i^{-\delta-2-2\gamma}}{i^{-1-\gamma}+\kappa} \\ & - 2\alpha^2 \kappa (1-\rho) \sum_{i=1}^{P} \frac{i^{-\delta-1-\gamma}}{i^{-1-\gamma}+\kappa},\end{aligned}$$

where $Q = 1 - \frac{1}{N} \sum_{i=1}^{P} \frac{i^{-2-2\gamma}}{(i^{-1-\gamma}+\kappa)^2}$.

Proof. First, we apply Lemma 26, coupled with the fact that $L_2^{\mathrm{det}}(\beta_1, \beta_2, \mathcal{D}_F, \lambda, N, \alpha) := L_1^{\mathrm{det}}(\beta_2, \beta_1, \mathcal{D}_F, \lambda, N, 1-\alpha)$, to see that:

$$\begin{aligned}Q \cdot \mathbb{E}_{\mathcal{D}_W}[L_2^{\mathrm{det}}] = {} & \kappa^2 (1 - 2\alpha^2(1-\rho)) \sum_{i=1}^{P} \frac{i^{-\delta-1-\gamma}}{(i^{-1-\gamma}+\kappa)^2} + \alpha^2 L^*(\rho) \\ & + 2\kappa(1-\rho)\alpha(1-2\alpha) \sum_{i=1}^{P} \frac{i^{-\delta-2(1+\gamma)}}{(i^{-1-\gamma}+\kappa)^2} \\ & + 2\alpha(1-\rho) \frac{1}{N} \left(\sum_{i=1}^{P} \frac{i^{-2-2\gamma}}{(i^{-1-\gamma}+\kappa)^2} \right) \cdot (1-2\alpha) \sum_{i=1}^{P} \frac{i^{-\delta-2-2\gamma}}{i^{-1-\gamma}+\kappa},\end{aligned}$$

where $Q = 1 - \frac{1}{N} \sum_{i=1}^{P} \frac{i^{-2-2\gamma}}{(i^{-1-\gamma}+\kappa)^2}$. Using that $(Q^{-1} - 1)\alpha^2 L^*(\rho) = Q^{-1} \frac{1}{N} \left(\sum_{i=1}^{P} \frac{i^{-2-2\gamma}}{(i^{-1-\gamma}+\kappa)^2} \right) 2\alpha^2(1-\rho) \left(\sum_{i=1}^{P} i^{-\delta-1-\gamma} \right)$, this means that:

$$\begin{aligned}\mathbb{E}_{\mathcal{D}_W}[L_2^{\mathrm{det}}] - \alpha^2 L^*(\rho) = {} & Q^{-1} \frac{1}{N} \left(\sum_{i=1}^{P} \frac{i^{-2-2\gamma}}{(i^{-1-\gamma}+\kappa)^2} \right) 2\alpha^2(1-\rho) \left(\sum_{i=1}^{P} i^{-\delta-1-\gamma} \right) \\ & + Q^{-1} \cdot \kappa^2 (1 - 2\alpha^2(1-\rho)) \sum_{i=1}^{P} \frac{i^{-\delta-1-\gamma}}{(i^{-1-\gamma}+\kappa)^2} \\ & + Q^{-1} 2\kappa(1-\rho)\alpha(1-2\alpha) \sum_{i=1}^{P} \frac{i^{-\delta-2(1+\gamma)}}{(i^{-1-\gamma}+\kappa)^2} \\ & + Q^{-1} 2\alpha(1-\rho) \frac{1}{N} \left(\sum_{i=1}^{P} \frac{i^{-2-2\gamma}}{(i^{-1-\gamma}+\kappa)^2} \right) \cdot (1-2\alpha) \sum_{i=1}^{P} \frac{i^{-\delta-2-2\gamma}}{i^{-1-\gamma}+\kappa}\end{aligned}$$

By expanding some of these terms, we see that:

$$\begin{aligned}\mathbb{E}_{\mathcal{D}_W}[L_2^{\mathrm{det}}] - \alpha^2 L^*(\rho) = {} & Q^{-1} 2\alpha^2 (1-\rho) \frac{1}{N} \left(\sum_{i=1}^{P} \frac{i^{-2-2\gamma}}{(i^{-1-\gamma}+\kappa)^2} \right) \cdot \sum_{i=1}^{P} i^{-\delta-1-\gamma} \\ & + Q^{-1} \cdot \kappa^2 \sum_{i=1}^{P} \frac{i^{-\delta-1-\gamma}}{(i^{-1-\gamma}+\kappa)^2} - Q^{-1} 2\alpha^2 (1-\rho) \cdot \kappa^2 \sum_{i=1}^{P} \frac{i^{-\delta-1-\gamma}}{(i^{-1-\gamma}+\kappa)^2} \\ & + Q^{-1} 2\kappa(1-\rho)\alpha(1-\alpha) \sum_{i=1}^{P} \frac{i^{-\delta-2(1+\gamma)}}{(i^{-1-\gamma}+\kappa)^2} - Q^{-1} 2\kappa(1-\rho)\alpha^2 \sum_{i=1}^{P} \frac{i^{-\delta-2(1+\gamma)}}{(i^{-1-\gamma}+\kappa)^2} \\ & + Q^{-1} 2\alpha(1-\alpha)(1-\rho) \frac{1}{N} \left(\sum_{i=1}^{P} \frac{i^{-2-2\gamma}}{(i^{-1-\gamma}+\kappa)^2} \right) \cdot \sum_{i=1}^{P} \frac{i^{-\delta-2-2\gamma}}{i^{-1-\gamma}+\kappa} \\ & - Q^{-1} 2\alpha^2 (1-\rho) \frac{1}{N} \left(\sum_{i=1}^{P} \frac{i^{-2-2\gamma}}{(i^{-1-\gamma}+\kappa)^2} \right) \cdot \sum_{i=1}^{P} \frac{i^{-\delta-2-2\gamma}}{i^{-1-\gamma}+\kappa}.\end{aligned}$$

When we collect terms, we obtain:

$$
\begin{aligned}
\mathbb{E}_{\mathcal{D}_W}[L_2^{\mathtt{det}}] - \alpha^2 L^*(\rho) &= Q^{-1} \cdot \kappa^2 \sum_{i=1}^{P} \frac{i^{-\delta-1-\gamma}}{(i^{-1-\gamma}+\kappa)^2} + Q^{-1} 2\kappa(1-\rho)\alpha(1-\alpha) \sum_{i=1}^{P} \frac{i^{-\delta-2(1+\gamma)}}{(i^{-1-\gamma}+\kappa)^2} \\
&+ Q^{-1} 2\alpha(1-\alpha)(1-\rho)\frac{1}{N}\left(\sum_{i=1}^{P} \frac{i^{-2-2\gamma}}{(i^{-1-\gamma}+\kappa)^2}\right) \cdot \sum_{i=1}^{P} \frac{i^{-\delta-2-2\gamma}}{i^{-1-\gamma}+\kappa} \\
&- Q^{-1} 2\kappa(1-\rho)\alpha^2 \left(\sum_{i=1}^{P} \frac{i^{-\delta-2(1+\gamma)}}{(i^{-1-\gamma}+\kappa)^2} + \sum_{i=1}^{P} \frac{\kappa \cdot i^{-\delta-1-\gamma}}{(i^{-1-\gamma}+\kappa)^2}\right) \\
&+ Q^{-1} 2\alpha^2(1-\rho)\frac{1}{N}\left(\sum_{i=1}^{P} \frac{i^{-2-2\gamma}}{(i^{-1-\gamma}+\kappa)^2}\right) \cdot \left(\sum_{i=1}^{P} i^{-\delta-1-\gamma} - \sum_{i=1}^{P} \frac{i^{-\delta-2-2\gamma}}{i^{-1-\gamma}+\kappa}\right) \\
&= Q^{-1} \cdot \kappa^2 \sum_{i=1}^{P} \frac{i^{-\delta-1-\gamma}}{(i^{-1-\gamma}+\kappa)^2} + Q^{-1} 2\kappa(1-\rho)\alpha(1-\alpha) \sum_{i=1}^{P} \frac{i^{-\delta-2(1+\gamma)}}{(i^{-1-\gamma}+\kappa)^2} \\
&+ Q^{-1} 2\alpha(1-\alpha)(1-\rho)\frac{1}{N}\left(\sum_{i=1}^{P} \frac{i^{-2-2\gamma}}{(i^{-1-\gamma}+\kappa)^2}\right) \cdot \sum_{i=1}^{P} \frac{i^{-\delta-2-2\gamma}}{i^{-1-\gamma}+\kappa} \\
&- Q^{-1} 2\kappa(1-\rho)\alpha^2 \left(\sum_{i=1}^{P} \frac{i^{-\delta-1-\gamma}}{(i^{-1-\gamma}+\kappa)}\right) \\
&+ Q^{-1} 2\kappa\alpha^2(1-\rho)\frac{1}{N}\left(\sum_{i=1}^{P} \frac{i^{-2-2\gamma}}{(i^{-1-\gamma}+\kappa)^2}\right) \cdot \frac{i^{-\delta-1-\gamma}}{i^{-1-\gamma}+\kappa}.
\end{aligned}
$$

595 Combining the last two terms gives us the desired statement. □

596 Now, we are ready to prove Lemma 45.

Proof. For the first bound, we observe that:

$$
\begin{aligned}
&\alpha^2 L^*(\rho) - \mathbb{E}_{\mathcal{D}_W}[L_2^{\mathtt{det}}] \\
&\leq_{(A)} 2\alpha^2\kappa(1-\rho)\sum_{i=1}^{P} \frac{i^{-\delta-1-\gamma}}{i^{-1-\gamma}+\kappa} \\
&=_{(B)} O\left(\alpha^2(1-\rho)\kappa^{\frac{\min(1+\gamma,\delta+\gamma)}{1+\gamma}}\right) \\
&=_{(C)} O\left(\kappa^{\frac{\nu}{1+\gamma}}\right) \\
&=_{(D)} O\left(\max(\lambda^{\frac{\nu}{1+\gamma}}, N^{-\nu})\right)
\end{aligned}
$$

597 where (A) uses Lemma 46, (B) uses Lemma 33, (C) uses that $\delta \leq 1$ and $\rho \in [0, 1)$, and (D) uses Lemma 35.

For the second bound, we observe that:

$$
\begin{aligned}
&\mathbb{E}_{\mathcal{D}_W}[L_2^{\texttt{det}}] - \alpha^2 L^*(\rho) \\
&\leq_{(A)} Q^{-1} \cdot \kappa^2 \sum_{i=1}^{P} \frac{i^{-\delta-1-\gamma}}{(i^{-1-\gamma}+\kappa)^2} \\
&+ Q^{-1} 2\kappa\alpha(1-\alpha)(1-\rho) \sum_{i=1}^{P} \frac{i^{-\delta-2(1+\gamma)}}{(i^{-1-\gamma}+\kappa)^2} \\
&+ Q^{-1} 2\alpha(1-\alpha)(1-\rho)\frac{1}{N} \left(\sum_{i=1}^{P} \frac{i^{-2-2\gamma}}{(i^{-1-\gamma}+\kappa)^2}\right) \cdot \sum_{i=1}^{P} \frac{i^{-\delta-2-2\gamma}}{i^{-1-\gamma}+\kappa} \\
&=_{(B)} O\left(\kappa^{\frac{\min(2(1+\gamma),\gamma+\delta)}{1+\gamma}} + \alpha(1-\alpha)(1-\rho)\kappa^{\frac{\min(1+\gamma,\gamma+\delta)}{1+\gamma}} + \alpha(1-\alpha)(1-\rho)\frac{\kappa^{-\frac{1}{1+\gamma}}}{N}\right) \\
&=_{(C)} O\left(\kappa^{\frac{\gamma+\delta}{1+\gamma}} + (1-\alpha)(1-\rho)\kappa^{\frac{\gamma+\delta}{1+\gamma}} + (1-\alpha)(1-\rho)\frac{\kappa^{-\frac{1}{1+\gamma}}}{N}\right) \\
&= O\left(\kappa^{\frac{\gamma+\delta}{1+\gamma}} + (1-\alpha)(1-\rho)\frac{\kappa^{-\frac{1}{1+\gamma}}}{N}\right) \\
&=_{(D)} O\left(\max(\lambda^{\frac{\nu}{1+\gamma}}, N^{-\nu}) + (1-\alpha)(1-\rho)\frac{\min(\lambda^{-\frac{1}{1+\gamma}}, N)}{N}\right)
\end{aligned}
$$

where (A) uses Lemma 46, (B) uses Lemma 33 and Lemma 34, (C) uses that $\delta \leq 1$ and $\alpha \geq 0.5$, and (D) uses Lemma 35. □

6.C. Extension of Theorem 4. We next study the market entry $\tilde{N}_E^*$ threshold in the environment of Theorem 4where the incumbent has *finite data* and the new company faces no safety constraint. We place the further assumption that $\delta \leq 1$. We compute the following upper bound on the modified market entry threshold.

Theorem 47 (Extension of Theorem 4). *Suppose that the power-law scaling assumptions hold with exponents $\gamma, \delta > 0$ and correlation coefficient $\rho \in [0, 1)$, and suppose that $P = \infty$. Assume that $\tau_E = \infty$. Suppose that the safety constraint τ_I satisfies (1). Then we have that $\tilde{N}_E^* = \tilde{N}_E^*(N_I, \tau_I, \infty, \mathcal{D}_W, \mathcal{D}_F)$ satisfies:*

$$
\tilde{N}_E^* := \begin{cases} O(N_I) & \text{if } N_I \leq \tilde{G}_I^{-\frac{1}{2\nu}}(1-\rho)^{-\frac{1}{2\nu}} \\ O\left(N_I^{\frac{1}{\nu+1}} \cdot \tilde{G}_I^{-\frac{1}{2(\nu+1)}}(1-\rho)^{-\frac{1}{2(\nu+1)}}\right) & \text{if } \tilde{G}_I^{-\frac{1}{2\nu}}(1-\rho)^{-\frac{1}{2\nu}} \leq N_I \leq \tilde{G}_I^{-\frac{1}{2}-\frac{1}{\nu}}(1-\rho)^{\frac{1}{2}} \\ O\left(\tilde{G}_I^{-\frac{1}{\nu}}\right) & \text{if } N_I \geq \tilde{G}_I^{-\frac{1}{2}-\frac{1}{\nu}}(1-\rho)^{\frac{1}{2}}, \end{cases}
$$

where $L^*(\rho) = \mathbb{E}_{\mathcal{D}_W}[(\beta_1 - \beta_2)^T \Sigma (\beta_1 - \beta_2)] = \Theta(1-\rho)$, where $\alpha^* = \sqrt{\frac{\min(\tau_I, L^*(\rho))}{L^*(\rho)}}$, where $\tilde{\alpha} := \sqrt{(1-\alpha^*) + (\alpha^*)^2}$, where $\tilde{G}_I = (1-\tilde{\alpha})^2(1-\rho)$, and where $\nu = \min(2(1+\gamma), \gamma+\delta) = \gamma + \delta$.

Theorem 47 shows that the key qualitative finding from Theorem 4—that the new company can enter with $N_E = o(N_I)$ data as long as the incumbent's dataset size is sufficiently large—readily extends to this setting. We note that the bound in Theorem 47 and the bound in Theorem 4take slightly different forms: the term $G_I = (\sqrt{L^*(\rho)} - \sqrt{\min(L^*(\rho), \tau_I)})^2 = \Theta((1-\alpha^*)^2(1-\rho))$ is replaced by $\tilde{G}_I = (1-\tilde{\alpha})^2(1-\rho)$. We expect some of these differences arise because the bound in Theorem 47 is not tight, rather than fundamental distinctions between the two settings. Proving a tight bound on the modified market entry threshold is an interesting direction for future work.

To prove this, we compute a lower bound on the incumbent's loss $\mathbb{E}_{\mathcal{D}_W}[L_1^*(\beta_1, \beta_2, \mathcal{D}_F, \tilde{\lambda}_I, N_I, \tilde{\alpha}_I)]$.

Lemma 48. *Suppose that the power-law scaling assumptions hold with exponents $\gamma, \delta > 0$ and correlation coefficient $\rho \in [0, 1)$, and suppose that $P = \infty$. Assume that $\tau_E = \infty$. Suppose that the safety constraint τ_I satisfies (1). Then we have that:*

$$
\begin{aligned}
&\mathbb{E}_{\mathcal{D}_W}[L_1^*(\beta_1, \beta_2, \mathcal{D}_F, \tilde{\lambda}_I, N_I, \tilde{\alpha}_I)] \\
&= \begin{cases} \Omega\left(N_I^{-\nu}\right) & \text{if } N_I \leq \tilde{G}_I^{-\frac{1}{2\nu}}(1-\rho)^{-\frac{1}{2\nu}} \\ \Omega\left(N_I^{-\frac{\nu}{\nu+1}} \cdot \tilde{G}_I^{\frac{\nu}{2(\nu+1)}}(1-\rho)^{\frac{\nu}{2(\nu+1)}}\right) & \text{if } \tilde{G}_I^{-\frac{1}{2\nu}}(1-\rho)^{-\frac{1}{2\nu}} \leq N_I \leq \tilde{G}_I^{-\frac{1}{2}-\frac{1}{\nu}}(1-\rho)^{\frac{1}{2}} \\ \Omega\left(\tilde{G}_I\right) & \text{if } N_I \geq \tilde{G}_I^{-\frac{1}{2}-\frac{1}{\nu}}(1-\rho)^{\frac{1}{2}}. \end{cases}
\end{aligned}
$$

where $L^*(\rho) = \mathbb{E}_{\mathcal{D}_W}[(\beta_1-\beta_2)^T\Sigma(\beta_1-\beta_2)] = \Theta(1-\rho)$*, where* $\alpha^* = \sqrt{\frac{\min(\tau_I, L^*(\rho))}{L^*(\rho)}}$*, where* $\tilde{\alpha} := \sqrt{(1-\alpha^*)+(\alpha^*)^2}$*, where* $\tilde{G}_I = (1-\tilde{\alpha})^2(1-\rho)$ *and where* $\nu = \min(2(1+\gamma), \gamma+\delta) = \gamma+\delta$.

Proof. By Corollary 8and Lemma 35, we know that:

$$\mathbb{E}_{\mathcal{D}_W}[L_1^*(\beta_1,\beta_2,\mathcal{D}_F,\tilde{\lambda}_I,N_I,\tilde{\alpha}_I)] = \Omega(\kappa^{\frac{\nu}{1+\gamma}}) = \Omega(\max(\lambda^{\frac{\nu}{1+\gamma}}, N_I^{-\nu})).$$

Let $C_{\delta,\gamma}$ be an implicit constant[§] such that:

$$\mathbb{E}_{\mathcal{D}_W}[L_1^*(\beta_1,\beta_2,\mathcal{D}_F,\tilde{\lambda}_I,N_I,\tilde{\alpha}_I)] \geq C_{\delta,\gamma}\max(\lambda^{\frac{\nu}{1+\gamma}}, N_I^{-\nu}) \quad [16]$$

By Lemma 45, there also exists an implicit constant $C'_{\delta,\gamma}$ such that:

$$\alpha^2 L^*(\rho) - \mathbb{E}_{\mathcal{D}_W}[L_2^{\text{det}}(\beta_1,\beta_2,\mathcal{D}_F,\lambda,N_I,\alpha)] \leq C'_{\delta,\gamma}\max(\lambda^{\frac{\nu}{1+\gamma}}, N_I^{-\nu}). \quad [17]$$

We now split into two cases: (1) $\frac{C'_{\delta,\gamma}}{C_{\delta,\gamma}}\mathbb{E}_{\mathcal{D}_W}[L_1^*(\beta_1,\beta_2,\mathcal{D}_F,\tilde{\lambda}_I,N_I,\tilde{\alpha}_I)] \geq (1-\alpha^*)L^*(\rho)$, and (2) $\frac{C'_{\delta,\gamma}}{C_{\delta,\gamma}}\mathbb{E}_{\mathcal{D}_W}[L_1^*(\beta_1,\beta_2,\mathcal{D}_F,\tilde{\lambda}_I,N_I,\tilde{\alpha}_I)] \leq (1-\alpha^*)L^*(\rho)$.

Case 1: $\frac{C'_{\delta,\gamma}}{C_{\delta,\gamma}}\mathbb{E}_{\mathcal{D}_W}[L_1^*(\beta_1,\beta_2,\mathcal{D}_F,\tilde{\lambda}_I,N_I,\tilde{\alpha}_I)] \geq (1-\alpha^*)L^*(\rho)$**.** It follows from Eq. (16) that:

$$\mathbb{E}_{\mathcal{D}_W}[L_1^*(\beta_1,\beta_2,\mathcal{D}_F,\tilde{\lambda}_I,N_I,\tilde{\alpha}_I)] \geq C_{\delta,\gamma}\max(\lambda^{\frac{\nu}{1+\gamma}}, N_I^{-\nu}) \geq C_{\delta,\gamma}N_I^{-\nu}.$$

Using the condition for this case, this implies that:

$$\begin{aligned} N_I &\leq \left(\frac{1}{C_{\delta,\gamma}}\mathbb{E}_{\mathcal{D}_W}[L_1^*(\beta_1,\beta_2,\mathcal{D}_F,\tilde{\lambda}_I,N_I,\tilde{\alpha}_I)]\right)^{-\frac{1}{\nu}} \\ &\leq \left(\frac{1}{C'_{\delta,\gamma}}(1-\alpha^*)L^*(\rho)\right)^{-\frac{1}{\nu}} \\ &= O\left(((1-\tilde{\alpha})(1-\rho))^{-\frac{1}{\nu}}\right) \\ &= O\left(\tilde{G}_I^{-\frac{1}{2\nu}}(1-\rho)^{-\frac{1}{2\nu}}\right). \end{aligned}$$

This proves that N_I is up to constants within the first branch of the expression in the lemma statement. Since the bound in the lemma statement only changes by constants (that depend on δ and γ) between the first branch and second branch, this proves the desired expression for this case.

Case 2: $\frac{C'_{\delta,\gamma}}{C_{\delta,\gamma}}\mathbb{E}_{\mathcal{D}_W}[L_1^*(\beta_1,\beta_2,\mathcal{D}_F,\tilde{\lambda}_I,N_I,\tilde{\alpha}_I)] \leq (1-\alpha^*)L^*(\rho)$**.** Note that $\alpha^* = \sqrt{\frac{\min(\tau_I, L^*(\rho))}{L^*(\rho)}}$ is the mixture parameter that achieves the safety constraint in the infinite-data ridgeless setting. The incumbent's safety constraint means that:

$$\mathbb{E}_{\mathcal{D}_W}[L_2^{\text{det}}(\beta_1,\beta_2,\mathcal{D}_F,\tilde{\lambda}_I,N_I,\tilde{\alpha}_I)] \leq (\alpha^*)^2 L^*(\rho).$$

By Eq. (17), this implies that

$$(\tilde{\alpha}_I)^2 L^*(\rho) \leq C'_{\delta,\gamma}\cdot\max(\lambda^{\frac{\delta+\gamma}{1+\gamma}}, N_I^{-\delta-\gamma}) + (\alpha^*)^2 L^*(\rho).$$

Now, applying Eq. (16) and the assumption for this case, we see that:

$$\begin{aligned} (\tilde{\alpha}_I)^2 L^*(\rho) &\leq \frac{C'_{\delta,\gamma}}{C_{\delta,\gamma}}\cdot\mathbb{E}_{\mathcal{D}_W}[L_1^*(\beta_1,\beta_2,\mathcal{D}_F,\tilde{\lambda}_I,N_I,\tilde{\alpha}_I)] + (\alpha^*)^2 L^*(\rho) \\ &\leq (1-\alpha^*)L^*(\rho) + (\alpha^*)^2 L^*(\rho). \end{aligned}$$

This implies that:

$$\tilde{\alpha}_I \leq \sqrt{(1-\alpha^*)+(\alpha^*)^2}.$$

[§]We need to introduce an implicit constant because of $O()$ is permitted to hide constants that depend on δ and γ.

Let $\tilde{\alpha} := \sqrt{(1-\alpha^*) + (\alpha^*)^2}$. Plugging this into Corollary 8, we see that:

$$
\begin{aligned}
&\mathbb{E}_{\mathcal{D}_W}[L_1^*(\beta_1, \beta_2, \mathcal{D}_F, \tilde{\lambda}_I, N_I, \tilde{\alpha}_I)] \\
&\geq \inf_{\alpha \in [0.5, \tilde{\alpha}]} \inf_{\lambda > 0} \mathbb{E}_{\mathcal{D}_W}[L_1^*(\beta_1, \beta_2, \mathcal{D}_F, \lambda, N_I, \alpha)] \\
&= \Theta\left(\inf_{\lambda > 0} \mathbb{E}_{\mathcal{D}_W}\left[L_1^*\left(\beta_1, \beta_2, \Sigma, \lambda, N_I, \tilde{\alpha}\right)\right]\right) \\
&= \begin{cases} \Theta\left(N_I^{-\nu}\right) & \text{if } N_I \leq (1-\tilde{\alpha})^{-\frac{1}{\nu}}(1-\rho)^{-\frac{1}{\nu}} \\ \Theta\left(\left(\frac{N_I}{(1-\tilde{\alpha})(1-\rho)}\right)^{-\frac{\nu}{\nu+1}}\right) & \text{if } (1-\tilde{\alpha})^{-\frac{1}{\nu}}(1-\rho)^{-\frac{1}{\nu}} \leq N_I \leq (1-\tilde{\alpha})^{-\frac{2+\nu}{\nu}}(1-\rho)^{-\frac{1}{\nu}} \\ \Theta((1-\tilde{\alpha})^2(1-\rho)) & \text{if } N_I \geq (1-\tilde{\alpha})^{-\frac{2+\nu}{\nu}}(1-\rho)^{-\frac{1}{\nu}}, \end{cases} \\
&= \begin{cases} \Theta\left(N_I^{-\nu}\right) & \text{if } N_I \leq \tilde{G}_I^{-\frac{1}{2\nu}}(1-\rho)^{-\frac{1}{2\nu}} \\ \Theta\left(N_I^{-\frac{\nu}{\nu+1}} \cdot \tilde{G}_I^{\frac{\nu}{2(\nu+1)}}(1-\rho)^{\frac{\nu}{2(\nu+1)}}\right) & \text{if } \tilde{G}_I^{-\frac{1}{2\nu}}(1-\rho)^{-\frac{1}{2\nu}} \leq N_I \leq \tilde{G}_I^{-\frac{1}{2}-\frac{1}{\nu}}(1-\rho)^{\frac{1}{2}} \\ \Theta\left(\tilde{G}_I\right) & \text{if } N_I \geq \tilde{G}_I^{-\frac{1}{2}-\frac{1}{\nu}}(1-\rho)^{\frac{1}{2}}. \end{cases}
\end{aligned}
$$

The statement follows in this case. □

We are now ready to prove Theorem 47.

Proof of Theorem 47. We analyze $(\tilde{\alpha}_C, \tilde{\lambda}_C)$ first for the incumbent $C = I$ and then for the entrant $C = E$. Like in the theorem statement, let $L^*(\rho) = \mathbb{E}_{\mathcal{D}_W}[(\beta_1 - \beta_2)^T \Sigma(\beta_1 - \beta_2)] = \Theta(1-\rho)$ (Claim 13) and $G_I := (\sqrt{L^*(\rho)} - \sqrt{\min(\tau_I, L^*(\rho))})^2$, and $\nu = \min(2(1+\gamma), \delta + \gamma)$.

Analysis of the incumbent $C = I$. We apply Lemma 48 to see that:

$$
\begin{aligned}
&\mathbb{E}_{\mathcal{D}_W}[L_1^*(\beta_1, \beta_2, \mathcal{D}_F, \tilde{\lambda}_I, N_I, \tilde{\alpha}_I)] \\
&= \begin{cases} \Omega\left(N_I^{-\nu}\right) & \text{if } N_I \leq \tilde{G}_I^{-\frac{1}{2\nu}}(1-\rho)^{-\frac{1}{2\nu}} \\ \Omega\left(N_I^{-\frac{\nu}{\nu+1}} \cdot \tilde{G}_I^{\frac{\nu}{2(\nu+1)}}(1-\rho)^{\frac{\nu}{2(\nu+1)}}\right) & \text{if } \tilde{G}_I^{-\frac{1}{2\nu}}(1-\rho)^{-\frac{1}{2\nu}} \leq N_I \leq \tilde{G}_I^{-\frac{1}{2}-\frac{1}{\nu}}(1-\rho)^{\frac{1}{2}} \\ \Omega\left(\tilde{G}_I\right) & \text{if } N_I \geq \tilde{G}_I^{-\frac{1}{2}-\frac{1}{\nu}}(1-\rho)^{\frac{1}{2}}. \end{cases}.
\end{aligned}
$$

Analysis of the entrant $C = E$. Since the entrant faces no safety constraint, the entrant can choose any $\alpha \in [0.5, 1]$. We apply Corollary 7to see that:

$$
\mathbb{E}_{\mathcal{D}_W}[L_1^*(\beta_1, \beta_2, \mathcal{D}_F, \tilde{\lambda}_E, N, \tilde{\alpha}_E)] = \inf_{\alpha \in [0.5, 1]} \inf_{\lambda > 0} \mathbb{E}_{\mathcal{D}_W}[L_1^*(\beta_1, \beta_2, \mathcal{D}_F, \lambda, N, \alpha)] = \Theta\left(N^{-\nu}\right),
$$

which means that:

$$
N_E^*(N_I, \tau_I, \infty, \mathcal{D}_W, \mathcal{D}_F) = \begin{cases} O\left(N_I\right) & \text{if } N_I \leq \tilde{G}_I^{-\frac{1}{2\nu}}(1-\rho)^{-\frac{1}{2\nu}} \\ O\left(N_I^{\frac{1}{\nu+1}} \cdot \tilde{G}_I^{-\frac{1}{2(\nu+1)}}(1-\rho)^{-\frac{1}{2(\nu+1)}}\right) & \text{if } \tilde{G}_I^{-\frac{1}{2\nu}}(1-\rho)^{-\frac{1}{2\nu}} \leq N_I \leq \tilde{G}_I^{-\frac{1}{2}-\frac{1}{\nu}}(1-\rho)^{\frac{1}{2}} \\ O\left(\tilde{G}_I^{-\frac{1}{\nu}}\right) & \text{if } N_I \geq \tilde{G}_I^{-\frac{1}{2}-\frac{1}{\nu}}(1-\rho)^{\frac{1}{2}} \end{cases}
$$

as desired.

□

6.D. Extension of Theorem 5. We next study the market entry $\tilde{N}_E^*$ threshold in the environment of Theorem 5where the incumbent has infinite data and the new company faces a *nontrivial safety constraint*. We place the further assumption that $\delta \leq 1$. We compute the following upper bound on the modified market entry threshold.

Theorem 49 (Extension of Theorem 5)**.** *Suppose that the power-law scaling assumptions hold with exponents $\gamma, \delta > 0$ and correlation coefficient $\rho \in [0, 1)$, and suppose that $P = \infty$. Suppose that the safety constraints τ_I and τ_E satisfy (2). Then it holds that $\tilde{N}_E^* = \tilde{N}_E^*(\infty, \tau_I, \tau_E, \mathcal{D}_W, \mathcal{D}_F)$ satisfies:*

$$
\tilde{N}_E^* := O\left(\max\left(\tilde{D}^{-\frac{1}{\nu}}, \tilde{D}^{-\frac{\nu+1}{\nu}}\left(G_E^{\frac{1}{2}}(1-\rho)^{\frac{1}{2}} + \frac{1}{2}G_I - \frac{1}{2}G_E\right)\right)\right),
$$

where $L^*(\rho) = \mathbb{E}_{\mathcal{D}_W}[(\beta_1 - \beta_2)^T \Sigma(\beta_1 - \beta)] = \Theta(1-\rho)$, where $\nu = \min(2(1+\gamma), \delta+\gamma) = \delta+\gamma$, where $G_I := \left(\sqrt{L^*(\rho)} - \sqrt{\min(\tau_I, L^*(\rho))}\right)^2$ and $G_E := \left(\sqrt{L^*(\rho)} - \sqrt{\min(\tau_E, L^*(\rho))}\right)^2$, and where:

$$
\tilde{D} := \alpha_E^* \cdot (G_I - G_E) - \frac{(G_I - G_E)^2}{4 \cdot L^*(\rho)}.
$$

Theorem 49 shows that the key qualitative finding from Theorem 5—that the new company can enter with finite data, as long as they face a strictly weaker safety constraint than the incumbent company—readily extends to this setting. We note that the bound in Theorem 49 and the bound in Theorem 5take slightly different forms. Some of these differences are superficial: while the bound in Theorem 49 contains two—rather than three—regimes, the third regime in Theorem 5does not exist in the case where $\delta \le 1$. Other differences are more substantial: for example, the bound in Theorem 49 scales with $\tilde{D}$ while the bound in Theorem 5scales with D. However, we expect some of this difference arises because the bound in Theorem 49 is not tight, rather than fundamental distinctions between the two settings. Proving a tight bound on the modified market entry threshold is an interesting direction for future work.

We compute an upper bound on the number of data points N_E that the new company needs to achieve at most loss $\left(\sqrt{L^*(\rho)} - \sqrt{\min(\tau_I, L^*(\rho))}\right)^2$ on performance.

Lemma 50. *Suppose that the power-law scaling assumptions hold with exponents $\gamma, \delta > 0$ and correlation coefficient $\rho \in [0, 1)$, and suppose that $P = \infty$. Suppose that the safety constraints τ_I and τ_E satisfy (1). For sufficiently large constant $C_{\delta,\gamma}$, if*

$$N_E \ge C_{\delta,\gamma} \cdot \max\left(\tilde{D}^{-\frac{1}{\nu}}, \tilde{D}^{-\frac{\nu+1}{\nu}}\left(G_E^{\frac{1}{2}}(1-\rho)^{\frac{1}{2}} + \frac{1}{2}G_I - \frac{1}{2}G_E\right)\right),$$

then it holds that:

$$\mathbb{E}_{\mathcal{D}_W}[L_1^*(\beta_1, \beta_2, \mathcal{D}_F, \tilde{\lambda}_E, N_E, \tilde{\alpha}_E)] \le G_I,$$

where $L^*(\rho) = \mathbb{E}_{\mathcal{D}_W}[(\beta_1 - \beta_2)^T \Sigma(\beta_1 - \beta)] = \Theta(1-\rho)$, where $\nu = \min(2(1+\gamma), \delta+\gamma) = \delta+\gamma$, where $G_I := \left(\sqrt{L^*(\rho)} - \sqrt{\min(\tau_I, L^*(\rho))}\right)^2$ and $G_E := \left(\sqrt{L^*(\rho)} - \sqrt{\min(\tau_E, L^*(\rho))}\right)^2$, and where:

$$\tilde{D} := \alpha_E^* \cdot (G_I - G_E) - \frac{(G_I - G_E)^2}{4 \cdot L^*(\rho)}.$$

Proof. It suffices to construct $\tilde{\alpha}$ and $\tilde{\lambda}$ such that

$$\mathbb{E}_{\mathcal{D}_W}[\tilde{L}_2(\beta_1, \beta_2, \mathcal{D}_F, \tilde{\lambda}, N_E, \tilde{\alpha})] \le \tau_E$$

and

$$\mathbb{E}_{\mathcal{D}_W}[L_1^*(\beta_1, \beta_2, \mathcal{D}_F, \tilde{\lambda}, N_E, \tilde{\alpha})] \le G_I$$

for $N_E = \Omega\left(\max\left(\tilde{D}^{-\frac{1}{\nu}}, \tilde{D}^{-\frac{\nu+1}{\nu}}\left(G_E^{\frac{1}{2}}(1-\rho)^{\frac{1}{2}} + \frac{1}{2}G_I - \frac{1}{2}G_E\right)\right)\right)$.

To define $\tilde{\alpha}$ and $\tilde{\lambda}$, it is convenient to work with the following intermediate quantities. Let $\alpha_E^* = \left(\sqrt{L^*(\rho)} - \sqrt{\min(\tau_E, L^*(\rho))}\right)^2$ and let $\alpha_I^* = \left(\sqrt{L^*(\rho)} - \sqrt{\min(\tau_I, L^*(\rho))}\right)^2$. We define an error function:

$$f(N_E, \alpha, \lambda) := \max(\lambda^{\frac{\nu}{1+\gamma}}, N_E^{-\nu}) + (1-\alpha)(1-\rho)\frac{\min(\lambda^{-\frac{1}{1+\gamma}}, N_E)}{N_E}$$

We define:

$$\tilde{\alpha} := \alpha_E^* + \frac{1}{2}(1-\alpha_E^*)^2 - \frac{1}{2}(1-\alpha_I^*)^2 = \alpha_I^* + \frac{\alpha_E^* - \alpha_I^*}{2}.$$

and

$$\tilde{\lambda} := \inf_{\lambda \in (0,1)} f(N_E, \tilde{\alpha}, \lambda).$$

At these values of $\tilde{\alpha}$ and $\tilde{\lambda}$ and under the condition on N_E, observe that:

$$\begin{aligned} f(N_E, \tilde{\alpha}, \tilde{\lambda}) &= \Theta\left(\max\left(N_E^{-\nu}, \left(\frac{N_E}{(1-\tilde{\alpha})(1-\rho)}\right)^{-\frac{\nu}{\nu+1}}\right)\right) \\ &= \Theta\left(\max\left(N_E^{-\nu}, \left(\frac{N_E}{G_E^{\frac{1}{2}}(1-\rho)^{\frac{1}{2}} + \frac{1}{2}G_I + \frac{1}{2}G_E}\right)^{-\frac{\nu}{\nu+1}}\right)\right) \\ &= O\left(\tilde{D}\right), \end{aligned}$$

where the implicit constant can be reduced by increasing the implicit constant on N_E.

The remainder of the analysis boils down to showing that $\mathbb{E}_{\mathcal{D}_W}[\tilde{L}_2(\beta_1, \beta_2, \mathcal{D}_F, \tilde{\lambda}, N_E, \tilde{\alpha})] \le \tau_E$ and $\mathbb{E}_{\mathcal{D}_W}[L_1^*(\beta_1, \beta_2, \mathcal{D}_F, \tilde{\lambda}, N_E, \tilde{\alpha})] \le G_I$. To show this, we first derive an error function and bound these losses in terms of the error function.

Bounding $\mathbb{E}_{\mathcal{D}_W}[\tilde{L}_2(\beta_1, \beta_2, \mathcal{D}_F, \tilde{\lambda}, N_E, \tilde{\alpha})] \le \tau_E$. Observe that:

$$
\begin{aligned}
&\mathbb{E}_{\mathcal{D}_W}[\tilde{L}_2(\beta_1, \beta_2, \mathcal{D}_F, \tilde{\lambda}, N_E, \tilde{\alpha})] \\
&=_{(A)} \tilde{\alpha}^2 L^*(\rho) + O\left(\max(\lambda^{\frac{\nu}{1+\gamma}}, N_E^{-\nu}) + (1-\alpha)(1-\rho)\frac{\min(\lambda^{-\frac{1}{1+\gamma}}, N_E)}{N_E}\right) \\
&= (\alpha_E^* + \frac{1}{2}(1-\alpha_E^*)^2 - \frac{1}{2}(1-\alpha_I^*)^2)L^*(\rho) + O\left(f(N_E, \tilde{\alpha})\right) \\
&\le \left((\alpha_E^*)^2 L^*(\rho) + \frac{((1-\alpha_I^*)^2 - (1-\alpha_E^*)^2)^2}{4} - \alpha_E^*((1-\alpha_I^*)^2 - (1-\alpha_E^*)^2)\right) L^*(\rho) + \tilde{D} \\
&= \tau_E + \frac{(G_I - G_E)^2}{4 \cdot L^*(\rho)} - \alpha_E^*(G_I - G_E)\alpha_E^* \cdot (G_I - G_E) - \frac{(G_I - G_E)^2}{4 \cdot L^*(\rho)} \\
&= \tau_E
\end{aligned}
$$

where (A) follows from Lemma 45. This gives us the desired bound.

Bounding $\mathbb{E}_{\mathcal{D}_W}[L_1^*(\beta_1, \beta_2, \mathcal{D}_F, \tilde{\lambda}, N_E, \tilde{\alpha})]$. Observe that:

$$
\begin{aligned}
&\mathbb{E}_{\mathcal{D}_W}[L_1^*(\beta_1, \beta_2, \mathcal{D}_F, \tilde{\lambda}, N_E, \tilde{\alpha})] \\
&=_{(A)} (1-\tilde{\alpha})^2 L^*(\rho) + O\left(\max(\lambda^{\frac{\nu}{1+\gamma}}, N_E^{-\nu}) + (1-\alpha)(1-\rho)\frac{\min(\lambda^{-\frac{1}{1+\gamma}}, N_E)}{N_E}\right) \\
&\le (1 - \alpha_E^* - \frac{1}{2}(1-\alpha_E^*)^2 + \frac{1}{2}(1-\alpha_I^*)^2)^2 L^*(\rho) + O\left(f(N_E, \tilde{\alpha})\right) \\
&\le \left((1-\alpha_E^*)^2 + \frac{((1-\alpha_I^*)^2 - (1-\alpha_E^*)^2)^2}{4} - (1-\alpha_E^*)((1-\alpha_I^*)^2 - (1-\alpha_E^*)^2)\right) L^*(\rho) + \tilde{D} \\
&\le G_E + (G_I - G_E)(1-\alpha_E^*) + \frac{(G_I - G_E)^2}{4L^*(\rho)} + \alpha_E^* \cdot (G_I - G_E) - \frac{(G_I - G_E)^2}{4 \cdot L^*(\rho)} \\
&= G_I.
\end{aligned}
$$

where (A) uses Theorem 9, coupled with the fact that $\delta \le 1$ (which means that $\nu' = \nu$, so the mixture finite-data error is subsumed by the finite-data error) and coupled with Lemma 35. This gives us the desired bound.

□

We are now ready to prove Theorem 49.

Proof of Theorem 49. We analyze $(\tilde{\alpha}_C, \tilde{\lambda}_C)$ first for the incumbent $C = I$ and then for the entrant $C = E$. Like in the theorem statement, let $L^*(\rho) = \mathbb{E}_{\mathcal{D}_W}[(\beta_1 - \beta_2)^T \Sigma(\beta_1 - \beta)] = \Theta(1-\rho)$, let $\nu = \min(2(1+\gamma), \delta + \gamma) = \delta + \gamma$, let $G_I := \left(\sqrt{L^*(\rho)} - \sqrt{\min(\tau_I, L^*(\rho))}\right)^2$ and $G_E := \left(\sqrt{L^*(\rho)} - \sqrt{\min(\tau_E, L^*(\rho))}\right)^2$, and let:

$$
\tilde{D} := \alpha_E^* \cdot (G_I - G_E) - \frac{(G_I - G_E)^2}{4 \cdot L^*(\rho)}.
$$

Analysis of the incumbent $C = I$. To compute $\tilde{\alpha}_I$ and $\tilde{\lambda}_I$, we apply Lemma 44. The assumption $\tau_I \ge \mathbb{E}_{\mathcal{D}_W}[L_2(\beta_1, \beta_2, \Sigma, 0.5)]$ in the lemma statement can be rewritten as $\tau_I \ge 0.25 L^*(\rho)$, which guarantees the assumptions in Lemma 44 are satisfied. By Lemma 44, we see that:

$$
\mathbb{E}_{\mathcal{D}_W}[L_1^*(\beta_1, \beta_2, \mathcal{D}_F, \tilde{\lambda}_I, \infty, \tilde{\alpha}_I)] = \left(\sqrt{L^*(\rho)} - \sqrt{\min(\tau_I, L^*(\rho))}\right)^2 = G_I.
$$

Analysis of the entrant $C = E$. We apply Lemma 50 to see for sufficiently large constant $C_{\delta,\gamma}$, if

$$
N_E \ge C_{\delta,\gamma} \cdot \max\left(\tilde{D}^{-\frac{1}{\nu}}, \tilde{D}^{-\frac{\nu+1}{\nu}}\left(G_E^{\frac{1}{2}}(1-\rho)^{\frac{1}{2}} + \frac{1}{2}G_I - \frac{1}{2}G_E\right)\right),
$$

then it holds that:

$$
\mathbb{E}_{\mathcal{D}_W}[L_1^*(\beta_1, \beta_2, \mathcal{D}_F, \tilde{\lambda}_E, N_E, \tilde{\alpha}_E)] \le G_I = \mathbb{E}_{\mathcal{D}_W}[L_1^*(\beta_1, \beta_2, \mathcal{D}_F, \tilde{\lambda}_I, \infty, \tilde{\alpha}_I)].
$$

This means that:

$$
\tilde{N}_E^* = O\left(\max\left(\tilde{D}^{-\frac{1}{\nu}}, \tilde{D}^{-\frac{\nu+1}{\nu}}\left(G_E^{\frac{1}{2}}(1-\rho)^{\frac{1}{2}} + \frac{1}{2}G_I - \frac{1}{2}G_E\right)\right)\right)
$$

as desired. □

References

1. H Cui, B Loureiro, F Krzakala, L Zdeborová, Generalization error rates in kernel regression: The crossover from the noiseless to noisy regime in *Advances in Neural Information Processing Systems 34: Annual Conference on Neural Information Processing Systems 2021, NeurIPS 2021, December 6-14, 2021, virtual.* pp. 10131–10143 (2021).
2. A Wei, W Hu, J Steinhardt, More than a toy: Random matrix models predict how real-world neural representations generalize in *International Conference on Machine Learning, ICML 2022, 17-23 July 2022, Baltimore, Maryland, USA*, Proceedings of Machine Learning Research. (PMLR), Vol. 162, pp. 23549–23588 (2022).
3. FR Bach, High-dimensional analysis of double descent for linear regression with random projections. *CoRR* **abs/2303.01372** (2023).
4. N Mallinar, et al., Benign, tempered, or catastrophic: Toward a refined taxonomy of overfitting in *Advances in Neural Information Processing Systems 35: Annual Conference on Neural Information Processing Systems 2022, NeurIPS 2022, New Orleans, LA, USA, November 28 - December 9, 2022*, eds. S Koyejo, et al. (2022).
